# Myxarylin: Total *In Vitro* Biosynthesis, Expansion of Substrate Scope, and Bioengineered Thioamidated Biarylitides

**DOI:** 10.1021/jacs.5c17257

**Published:** 2026-02-13

**Authors:** Asfandyar Sikandar, Lana Vianey, Kai Schließmann, Qiyao Shen, C. Logan Mackay, F. P. Jake Haeckl, Vlada B. Urlacher, James H. Naismith, Rolf Müller

**Affiliations:** † 443745 Helmholtz Institute for Pharmaceutical Research Saarland (HIPS), Helmholtz Center for Infection Research (HZI), Saarbrücken, 66123, Germany; ‡ The Rosalind Franklin Institute, Harwell Campus, Didcot OX11 0QX, U.K.; § Institute of Biochemistry, 9170 Heinrich-Heine University Düsseldorf, Düsseldorf 40225, Germany; ∥ 240364 University of Oxford, Division of Structural Biology, Oxford OX3 7BN, U.K.; ⊥ Department of Pharmacy, Pharmaceutical Biotechnology, Saarland University, Saarbrücken 66123, Germany

## Abstract

Biarylitides are a new class of ribosomally synthesized and post-translationally modified peptides (RiPPs) featuring the smallest reported precursor peptide and cytochrome P450-mediated cross-links. Here, we report the complete *in vitro* reconstitution of the myxobacterial biarylitide, myxarylin. We demonstrate that cross-linking is the first step and acts as a gatekeeper for downstream processing. The cytochrome P450 enzyme P450_BytO_ from the myxarylin biosynthetic gene cluster exhibits remarkable substrate tolerance, allowing biosynthesis of new-to-nature thioamidated biarylitides through an unprecedented modular precursor peptide engineering approach. Surprisingly, changes in the precursor peptide sequence resulted in a shift in the installation of the P450_BytO_-mediated modification from the expected C- to the N-terminus. Leader peptide removal follows cross-linking and is likely carried out by a prolyl oligopeptidase (POP), a member of the serine protease family. The last step of the pathway involves N-terminal methylation, which also prevents premature degradation of the pathway intermediates by the POP. The crystal structure of the methyltransferase in complex with SAH and myxarylin allowed us to rationalize its substrate selectivity and guide protein engineering to expand its substrate scope.

## Graphical abstract

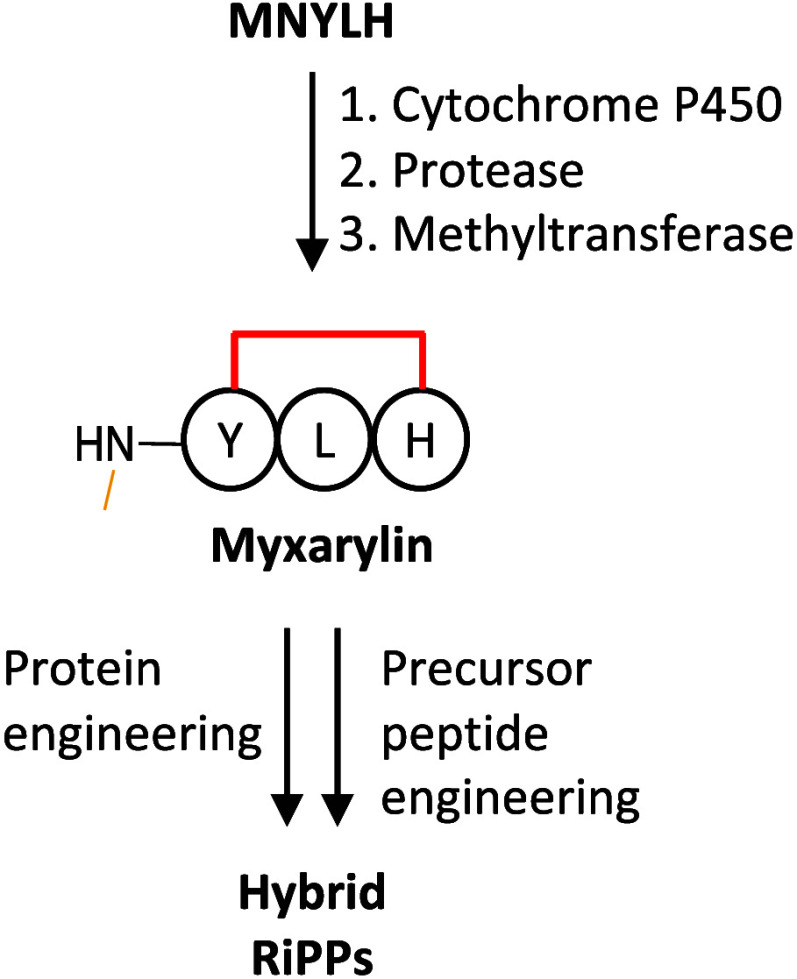

## Introduction

Ribosomally synthesized and post-translationally modified peptides (RiPPs) represent a structurally and functionally diverse group of natural products (NP).^1^ The array of characterized posttranslational modifications (PTMs) in RiPP systems is constantly increasing and includes cyclization, heterocyclization and various side-chain modifications giving rise to structurally complex NPs with diverse bioactivities.^1–4^ Recently, PTMs involving aromatic amino acid side-chain cross-linking have been particularly noteworthy, with numerous RiPP characterized featuring residues such as tyrosine, tryptophan, and histidine cross-linked to each other through their aromatic rings or to an aliphatic carbon of another amino acids (Figure 1a).^5^ Biarylitides are cyclic tripeptides containing a biaryl linkage between aromatic residues as their class-defining feature.^6^ The founding member of this group, biarylitides YYH and YFH, were isolated from *Planomonospora* genus, featuring a carbon–carbon (C–C) cross-link between residues Y and H.^7^ The identification of their biosynthetic gene clusters (BGCs) initiated the discovery of other biarylitides including gristide 834, Δ*N*-1_linked_, products of SlyP and ShyB (named after the respective P450 enzymes), and myxarylin (Figures 1b–d and S1).^8–12^

**Figure 1:**
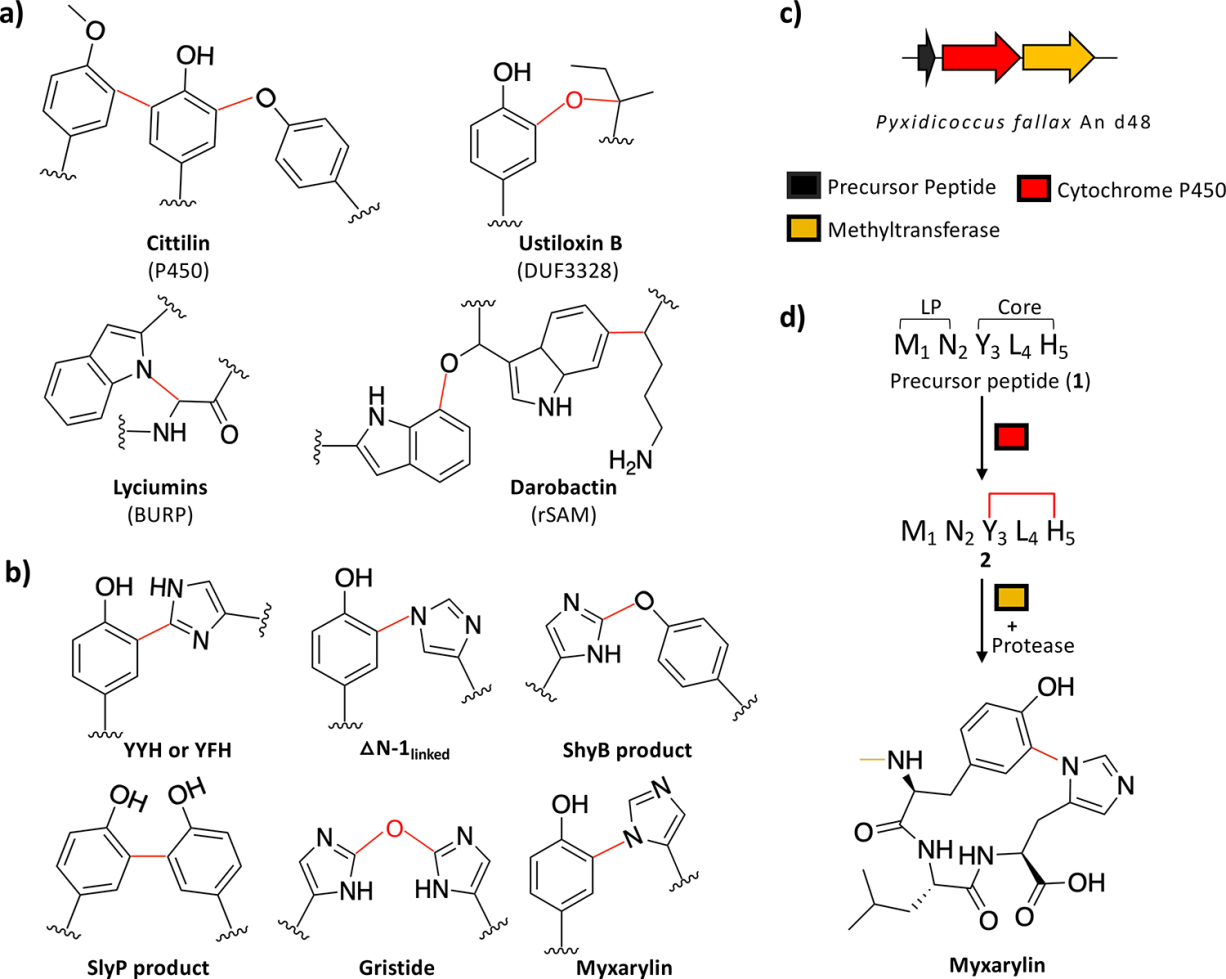
(a) Examples of cross-links found in RiPPs, along with the corresponding natural products and the enzyme classes responsible for installing these cross-links (red), are shown. (b) The type of aryl–aryl linkages reported for biarylitides.^6^ The associated BGCs can be found in Figure S1. (c) Organization of the myxarylin BGC. In comparison to other biarylitides, myxarylin BGC encodes an additional protein responsible for methylation. (d) The myxarylin pentapeptide precursor peptide (**1**) is first modified by cytochrome P450 to form a cyclic compound with the aryl linkage between Tyr3 and His5 (**2**). This is followed by methylation and proteolytic cleavage to form myxarylin. The order of modifications and the identity of the protease responsible for leader peptide removal is not known.^11^

The order of modifications was proposed based on *in vitro* studies using cytochrome P450 (P450_Blt_) from *Micromonospora* sp. MW-13.^9^ The first step involves the P450_Blt_ catalyzed formation of the biaryl-cyclized peptide via a C–N cross-link. Altering the substrate by removal of the leader peptide residues (positions 1 and 2) or substitution with bulky residues within the cyclic module (position 4) was found to be detrimental for P450_Blt_ activity. Of the substitutions at positions 3 and 5 that were tested, P450_Blt_ activity was limited to Tyr-Tyr, His-Tyr, Tyr-His, and Tyr-Trp cross-linking.^9^ The next step involves the removal of the leader peptide by as-yet unidentified protease, yielding the fully mature biarylitide RiPP.^6,9^ Given the high biarylitide-BGCs homology, myxarylin is also thought to follow a similar processing order. However, unlike other biarylitides reported to date, myxarylin BGC contains an additional gene, *bytz*, which encodes a putative S-adenosyl-methionine (SAM)-dependent methyltransferase proposed to catalyze N-terminal methylation after the removal of the leader peptide (Figure 1c,d).^11^ In short, our biosynthetic understanding beyond the P450-installed biaryl linkage is limited. Moreover, apart from P450_Blt_, the substrate scope and enzymatic potential of biarylitide P450s, particularly *in vitro*, remains largely unexplored, thereby limiting their application in peptide bioengineering, which given the minimal leader peptide requirement may have significant potential. To address these gaps in our understanding and expand the substrate scope of biarylitides-P450s, we selected myxarylin BGC as a model system.

## Results and Discussion

### *In Vitro* Characterization of P450_BytO_

We expressed and purified the cytochrome P450 from the myxarylin pathway (referred to as P450_BytO_; Figure S2a). Incubation of P450_BytO_ with sodium dithionite and subsequent fumigation with carbon monoxide led to the observation of the typical Soret band at 448 nm in the spectrum, indicating catalytically competent enzyme (Figure S2b).^13^ Since a typical redox partner required as cofactor for cytochrome P450 enzymes is not found in all known biarylitde BGCs, P450_BytO_ was incubated with precursor peptide (BytA, **1**), different electron transport systems and excess NAD­(P)H (Figures S3 and S4).^6^ The reaction mixture was analyzed by liquid chromatography–high resolution mass spectrometry (LC-HRMS), and only incubation with flavodoxin reductase (FdR, *Escherichia coli*) and flavodoxin (YkuN, *Bacillus subtilis*) resulted in the formation of a product exhibiting a loss of 2 Da (**2**, Figures 2a and S4a).^14,15^ We also noted Met sulfoxidation in these assays, likely due to the nonspecific oxidation of the precursor peptide caused by reactive oxygen species generated during the cytochrome P450 activation cycle (Figure S5a). Subsequently, tandem mass spectrometry (MS/MS) analysis confirmed the presence of the cross-link between Tyr3 and His5 (Figures S4b and S5b). Unlike microcin C7 biosynthesis, N-formylation of the initiator Meth of **1** had little impact on P450_BytO_ activity (**1** ^for^; Figures 2b and S6, S7).^16,17^ The removal of the first two amino acid residues abolished activity (**1** ^**–2A**^; Figures 2b and S8). These findings are in agreement with previously reported *in vitro* activity of P450_Blt_ and confirm that the first two residues constitute the leader peptide of the shortest precursor peptide reported to date, presenting intriguing biocatalytic potential for biarylitide P450s.^9^

**Figure 2:**
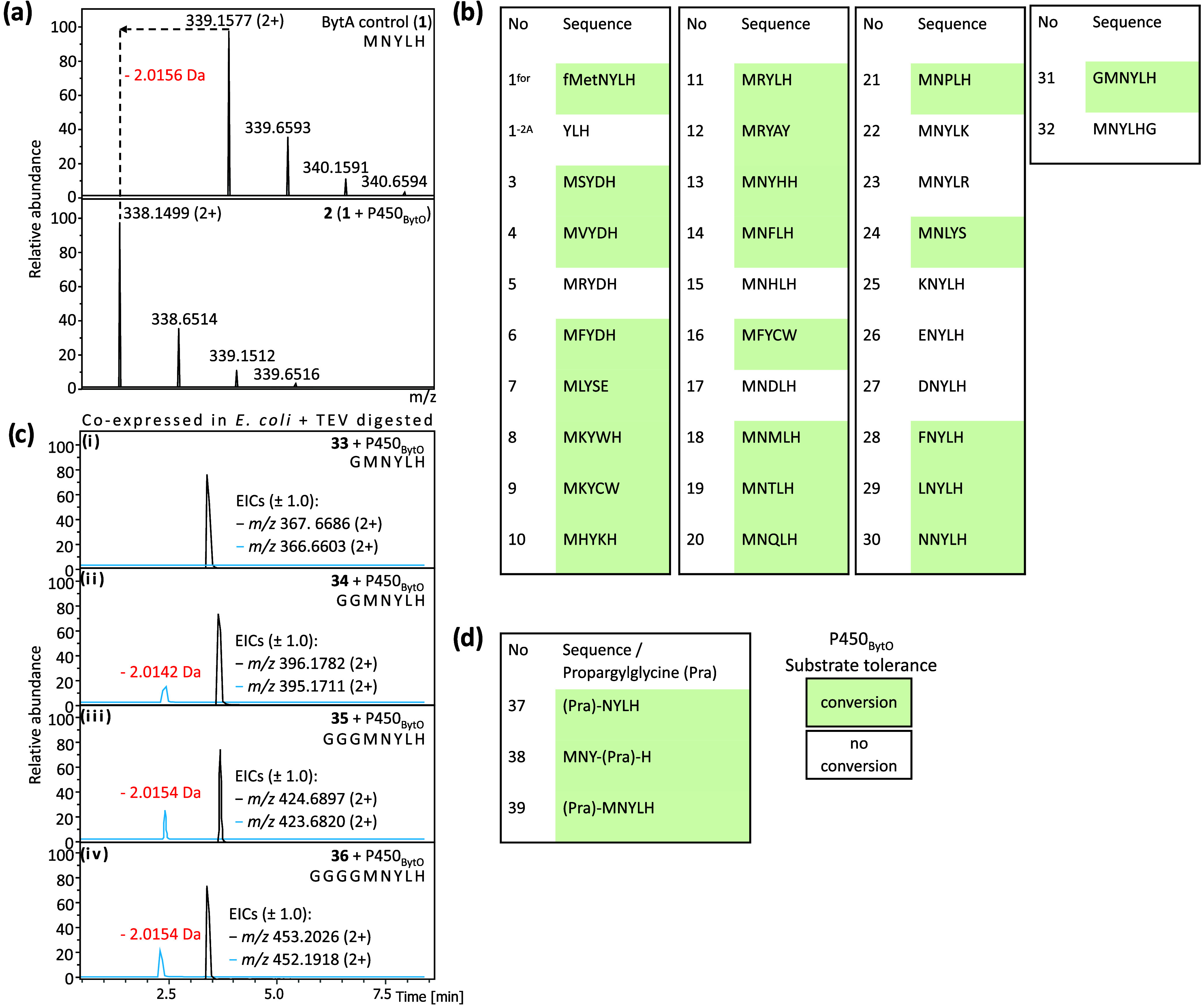
Exploring the substrate tolerance of P450_BytO_. (a) Extracted ion chromatogram (EIC) analysis of precursor peptide (**1**) after incubation with P450_BytO_ and all cofactors result in the production of a compound exhibiting a loss of 2 Da. (b) *In vitro* evaluation of P450_BytO_ substrate tolerance with alterations at residues P1 to P5 of the precursor peptide. (c) Analysis of **33**–**36** coexpressed with P450_BytO_ and redox partner (FdR/YkuN) in *E. coli*. (d) Incubation of P450_BytO_ with propargylglycine substituted precursor peptides (**37**–**39**). HPLC-ESI-MS and MS/MS analyses of **1**–**39** can be found in Figures S3–S8, S10–S26, S28–S39, and S49–S55.

### P450_BytO_ Substrate Scope and Engineering toward Thioamidated Biarylitides

To probe substrate promiscuity of biarylitide P450s, we generated a sequence similarity network (SSN) for P450_BytO_, and the genome context of the clusters associated with cytochrome P450s was analyzed either manually or by genome neighborhood network (GNN) tool to identify putative precursor peptides (Figure S9).^18,19^ P450_BytO_ readily processed many precursor peptides, showing a significantly higher tolerance for substitutions at positions 2 and 4 (**3**–**12**, Figures 2b and S10–S16) compared to P450_Blt_—these include aromatic and charged residues that were problematic for P450_Blt_. Since P450_BytO_ was able to accept Trp, Asp, His, and Lys substitutions at position 4, it appears that the size and charge of the residue inside biaryl module is not a bottleneck for P450_BytO_. Interestingly, in *Nocardia ninae* NBRC 108245, a P450_BytO_ homolog was found to be colocalized to a putative pentapeptide precursor peptide with a nonaryl residue at position 5 (**7**; Figure S12a). Surprisingly, P450_BytO_ was also able to modify this peptide (Figures 2b and S12b). We tested a series of substitutions at positions 1–5 and observed successful turnover for most variants, indicated by a loss of 2 Da (**13**–**30**, Figures 2b and S16–S25). With the exception of charged residues at position 1, the enzyme tolerated a range of amino acid changes at positions 1–5 of **1** (Figure 2b,d). Taken together, these findings confirm that P450_BytO_ exhibits remarkably relaxed substrate specificity and, intriguingly, is capable of modifying peptides with nonaryl substitutions at positions 3 or 5. Many RiPP pathways follow a leader peptide-guided biosynthesis logic, wherein tailoring enzymes bind to a recognition sequence that is separate from the peptide sequence that ends up as product.^20–26^ Consequently, significant changes to the leader (recognition) peptide are generally not well tolerated. Permissiveness of P450_BytO_ to changes in the leader peptide (positions 1 and 2) suggests substrate-engagement is length-dependent rather than sequence-dependent (Figure 2b). The ability of P450_BytO_ to act on such a diverse set of substrates sets it apart from the previously characterized biarylitide P450s.^9^

We tested whether P450_BytO_ can act on precursor peptides extended by one residue at each end of **1** (**31** and **32**). Unlike **32**, **31** was modified by P450_BytO_ (Figures 2b and S26). To rationalize the observed activity, we decided to build a model of P450_BytO_ in complex with heme group and **1** using AlphaFold 3 (Figure S27).^27^ The model suggests that **1** is buried in a small pocket with an orientation such that any C-terminal extension of **1** will likely cause a significant steric clash with the protein (Figure S27). In contrast, Met occupies a much larger pocket, with its amide bond exposed to solvent and directed away from the protein. Therefore, the Gly extension at the N-terminus is less likely to cause a clash with the protein, rationalizing the observed activity on **31** (Figure S27). Other biarylitide P450s have been shown to modify the corresponding precursor peptides when fused N-terminally to either SUMO-TEV or MBP-TEV.^8,10,12^ We further extended **1** by fusing with SUMO-TEV at the N-terminus (**33**) and coexpressed it with P450_BytO_ and FdR/YkuN in *E. coli* BL21 (DE3).^28,29^ After purification by Ni-NTA, the protein was digested by TEV protease and analyzed by LC-HRMS. We did not observe a loss of 2 Da (Figures 2c and S28), which we attributed to a clash between the TEV protease recognition site residues and the P450_BytO_ binding pocket, preventing optimal binding for catalysis. Therefore, we decided to test different flexible Gly linkers between SUMO-TEV and **1** (**34**–**36**; Figure 2c). We observed processing for **34**–**36**, and the subsequent MS/MS analyses were consistent with biaryl linkage between Tyr3 and His5 (Figures 2c and S29–S34). To confirm the nature of the biaryl linkage, we scaled up the heterologous expression to 60 L and purified the modified **34**. The position of the cross-link was determined from combined 1D and 2D NMR data (COSY, HSQC, HMBC, and ^15^N HMBC) (Figures S68–S74 and Table S2). The amount of aromatic C–H signals (HSQC) indicated the His-Tyr linkage to be located between C–N. NOESY, ROESY, and HMBC experiments did not provide sufficient evidence to assign which nitrogen is involved in the C–N linkage. ^15^N HMBC correlations provided support for a linkage between His-N10 and Tyr-C4, as correlations were observed between Tyr-H5 (δ_H_ 6.81) and His-N10 (δ_N_ 182.6), as well as between His-H3 (δ_H_ 7.08) and His-N10 (δ_N_ 182.6). This is, as all observed ^1^H, ^13^C, and ^15^N chemical shifts, in accordance with the published data for myxarylin, supporting the assignment of the cross-link between Tyr-C4 and His-N10 (Table S3).^11^ Biaryl motifs are an attractive chemical space for drug discovery and development; therefore, we tested propargylglycine (Pra) substitutions at positions −1, 1, and 3 (**37**–**39**; Figure 2d).^30,31^ All of these peptides were readily modified by P450_BytO_, thereby providing a facile route to biaryl peptides for future click-chemistry-mediated synthesis of biarylitide analogs (Figures 2d and S35–S36).^32,33^

To date, three main engineering strategies have been used to generate hybrid RiPPs: (i) chimeric leader peptide, (ii) leader peptide exchange via sortase A, and (iii) promiscuous PTM enzymes.^34–40^ Given the compact size of **1**, the minimal leader peptide requirement and amenability of P450_BytO_ to N-terminally extended **1**, we wondered if these features could be leveraged to construct a hybrid precursor peptide with a modular design comprising tandem leader peptide-core units: (leader–core_split_) _Module1_ – (leader–core) _Module2_ (Figure 3a). This modular architecture avoids the need to engineer substrate recognition by enzyme(s) involved in the modification of module 1 since the design preserves native recognition motifs in the leader peptide and, therefore, offers combinatorial flexibility.

**Figure 3:**
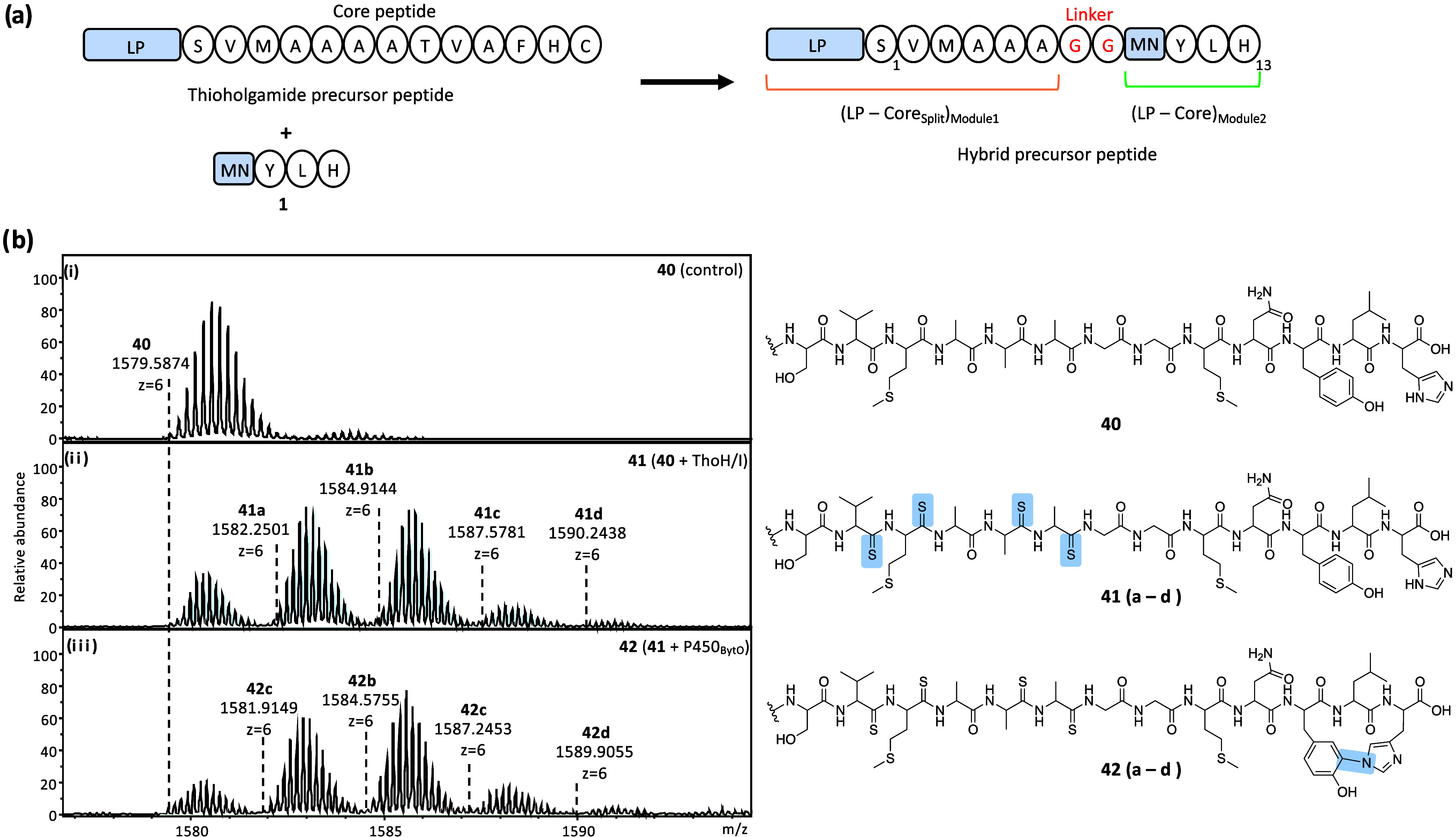
Production of thioamidated biarylitides. (a) Design of hybrid precursor peptide (**40**) based on thioholgamide and myxarylin precursor peptides (LP: leader peptide; light blue). (b) HPLC-ESI-MS analysis of **40** (i). Mass spectrum of **40** after incubation with ThoH/I in the presence of ATP and sodium sulfide (ii). Mass spectrum of **41** after incubation with P450_BytO_ and all cofactors (iii). **41**/**42a**–**d** differ in the number of thioamide bonds installed. Detailed MS and MS/MS analyses of **40**–**42** can be found in Figures S37–S43.

To demonstrate the feasibility and potential of this approach, we decided to focus on thioamidation—a rare and chemically intriguing PTM in nature that has been shown to enhance proteolytic stability of both linear and macrocyclic peptides.^41,42^ Two enzymes—ThoH and ThoI—are responsible for installing thioamide bonds in thioholgamide pathway.^43–45^ The hybrid precursor peptide, **40**, was constructed to serve as a substrate for both ThoH/I and P450_BytO_ (Figures 3a and S37). Upon treatment of **40** with ThoH/I, in the presence of ATP and sodium sulfide, we observed new peaks, consistent with the replacement of up to four oxygens by sulfur (**41a**–**41d**, Figures 3b and S38). MS/MS analysis localized the thioamidation to occur at the expected sites for the major products (**41a**, 1 thioamide bond; and **41b**, 2 thioamide bonds; Figures S39 and S40). These data also highlight that ThoH/I have a broad substrate acceptance and that the terminal precursor peptide Cys—conserved in all known thioholgamides—is not essential for thioamidation.^43–45^ Further incubation of **41** with P450_BytO_ resulted in the loss of 2 Da, with MS/MS confirming cross-linking between residues Tyr11 and His13 (**42**; Figures 3b, S41 and MS/MS Figures S42–S43). Compared to **42**, thioholgamides feature a larger macrocycle composed of six residues, with the postmacrocyclization Ser1-to-dehydroalanine (Dha) PTM installed by the ThoC/D complex.^43,44,46^ Attempts to install Dha on **42** were unsuccessful (Figure S44), suggesting substrate macrocycle size and/or residues influence ThoC/D activity. Collectively, these findings validate our modular design strategy for generating structurally diverse new-to-nature peptides. To the best of our knowledge, **42** represents the first thioamide-containing biarylitide, underscoring the remarkable plasticity of P450_BytO_ and RiPP enzymes more broadly in enabling the biosynthesis of new-to-nature molecules.

### Biarylitides C–C and C–N Bond Formation

YYH, YFH, and SlyP product are the only known biarylitides featuring C–C cross-links, despite the high structural similarity shared among biarylitide P450s (Figure S45a).^7,10^ To gain insights into the mechanism(s) controlling the cross-linking patterns, we generated AlphaFold 3 models of biarylitide P450s (YYH and P450_BytO_) with their cognate precursor peptides and compared them to the cocrystal structure of P450_Blt_ bound to its precursor peptide (pdb id: 8u2m; Figure S45b).^27,47^ Notably, the predicted AlphaFold 3 structures closely resemble the P450_Blt_ crystal structure, with the precursor peptides bound in a highly similar fashion (Figure S45b). In all P450_BytO_ models, the distance between C6 of Tyr3 and N19 of His5 of **1** is shorter than that with C5 of His5 of **1** (3.5 Å vs 3.8 Å; Figure S45b), which is consistent with the observed C–N bond formation in myxarylin.^11^ Therefore, we envisaged that subtle changes in the substrate positioning within the active site may influence the preference for C–C over C–N cross-linking. Using the P450_BytO_–**1** model as a starting point, we carried out comparative analysis of the active site pocket and identified several residues that may influence the regioselectivity (Figures 4a and S46). The cytochrome P450 residue His (His226, P450_BytO_; and His234, P450_Blt_) is located within an H-bond distance from His5 of the precursor peptide (Figure S46a). Interestingly, this position is occupied by Val or Leu in the P450s that catalyze C–C bond formation (Leu224, SlyP; Val211, P450_YYH_; and Val219 P450_YFH_; Figure S46b).^7,10^ To investigate its role, we generated a P450_BytO_^His226Val^ mutant (Figure S2a). This mutation rendered the enzyme inactive (Figure 4b). Similarly, Hansen *et al.* demonstrated that the His234Leu mutation in P450_Blt_ resulted in a significant drop in turnover.^47^ We quantified the effect of the mutation on substrate affinity and identified that the P450_BytO_^His226Val^ mutant showed affinity comparable to the wild-type P450_BytO_ enzyme for **1** (wt vs mutant *k*_d_ (μM): 9.31 and 5.95; Figure S47c). These findings support an important role for His226 in the catalysis of the precursor peptide in C–N-linked biarylitides.

**Figure 4:**
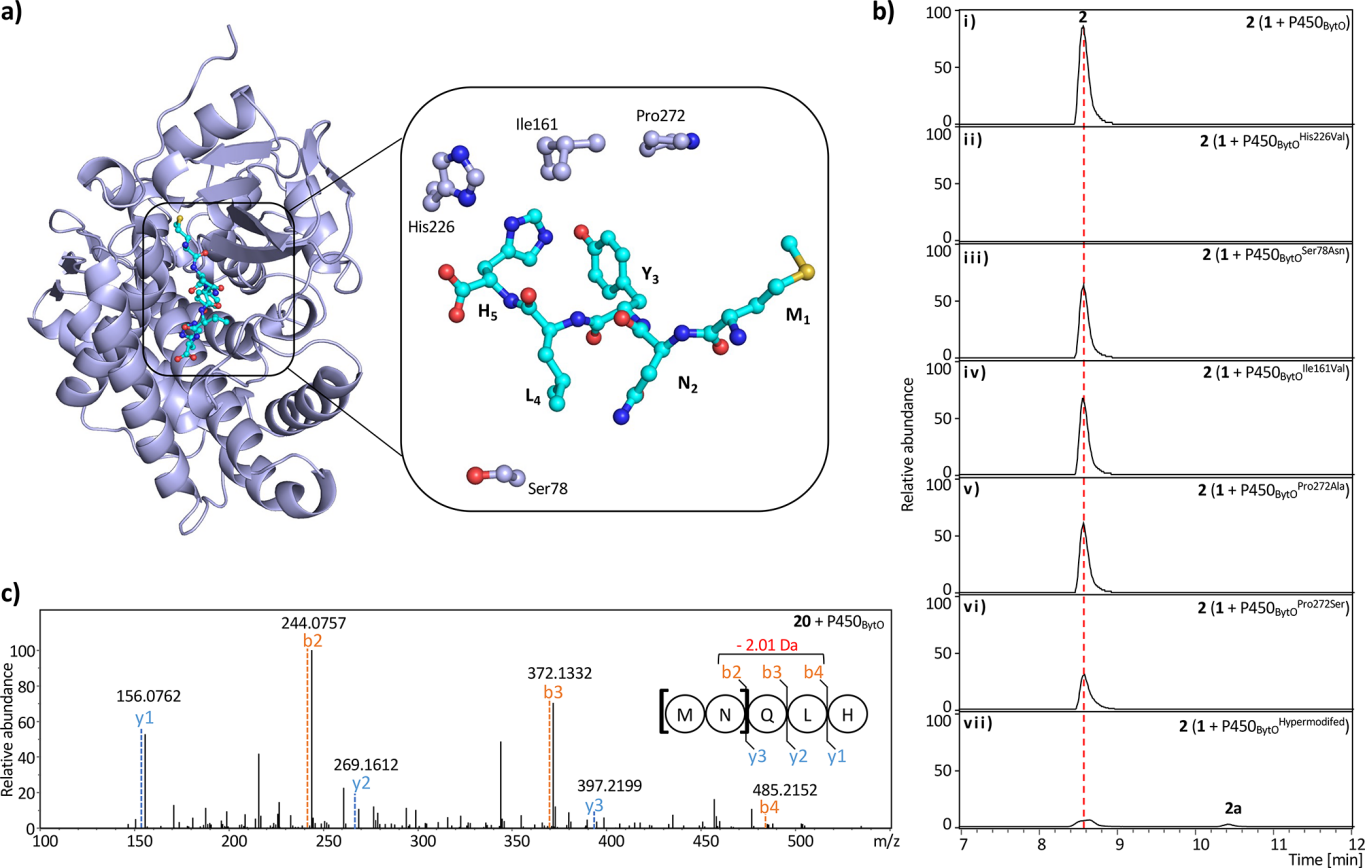
(a) AlphaFold 3 model of the P450_Byto_–**1** complex. A close-up view (rotated 90° clockwise) highlights the residues (stick, light blue) in close contact with **1** (stick, cyan) that were selected for site-directed mutagenesis. The overall mode of binding closely resembles that of the P450_Blt_–precursor peptide cocrystal structure (Figures S45 and S46). (b) HPLC-ESI-MS analysis of **1** after incubation with P450_BytO_ wild-type (i) and mutants (ii–vii). **2** and **2a** exhibit almost identical mass but differ in retention time. (c) MS/MS analysis of **20** after incubation with P450_BytO_. Sequence alignment of P450_BytO_^Hypermodified^ along with MS and MS/MS analyses of **2, 2a**, and **13**, as well as **18**–**21**, can be found in Figures S47–S55.

We subsequently generated additional mutants (Ser78Asn, Ile161Val, Pro272Ala, and Pro272Ser) targeting the substrate-binding pocket of P450_BytO_ (Figures 4a and S2a). All mutants retained catalytic activity, with products exhibiting retention times identical to those of the wild-type enzyme, consistent with C–N cross-linked biarylitide (**2**; Figure 4b). We engineered an extensively modified P450_BytO_ variant to mimic the substrate-binding pocket of P450_YYH_ (P450_BytO_^Hypermodified^; Figures S2a and S47).^7^ Upon incubation with **1**, we observed two new peaks (**2** and **2a**) with near identical masses but distinct retention times. Despite repeated efforts, we were unable to purify sufficient quantities of **2a** for in-depth structural characterization, and thus, the exact chemical nature of the linkage remains unresolved. However, MS/MS analysis points toward YxH linkage (Figure S49). Unlike P450_Blt_, incubation of **12**—precursor peptide with Tyr at positions 3 and 5—with P450_BytO_ yielded only a single product (Figure S50).^48^ These observations suggest that both the enzyme’s active site environment and the precursor peptide sequence modulate protein/substrate dynamics during catalysis, thereby dictating enzyme’s activity and regioselectivity. We examined the nonaryl precursor peptide variants (**7** and **18**–**21**). Unexpectedly, MS/MS analysis localized the loss of 2 Da to the first two residues (Figures 4c and S51–S55). Modification at the N-terminus rather than the expected C-terminal region suggests that the substrates adopt an alternative conformation in the active site of P450_BytO_. To our knowledge, no characterized biarylitide P450 or RiPP enzyme has been reported to exhibit such a drastic switch in the PTM installation site. These findings reiterate that P450_BytO_ substrate recognition is governed by the length rather than the sequence of the precursor peptide. Structural characterization studies are underway to identify the nature of the unexpected N-terminal modification that will help us better understand the molecular basis for peptide recognition and modification by P450_BytO_.

### *In Vitro* Reconstitution of *N*-Methylation and Structural Elucidation of CorZ in Complex with Myxarylin

With the biosynthesis of core scaffold established, we focused on the last two steps of the pathway, namely, methylation and the proteolytic removal of the leader peptide. The putative methyltransferase in myxarylin biosynthesis BytZ was expressed, but it was insoluble. The close homolog CorZ (93.18% sequence identity to BytZ) from *Corallococcus coralloides* MCy6431 was expressed and purified (Figure S2a). This protein originates from a cluster that is homologous to that of myxarylin (Figure S56 and Table S1).^11^ We first tested **1** and **2** as possible substrates for CorZ, but no methylation was detected (Figures 5a and S57). We determined the crystal structures of CorZ in complex with S-adenosylhomocysteine (SAH) and SAH/myxarylin to 2.5 and 2.7 Å resolution, respectively (Table S4). The overall shape of CorZ is typical of class-1 S-adenosylmethionine-dependent methyltransferase with a Rossman fold, and GxGxG motif located close to the SAH (Figures 5b and S58a).^49^ A large (∼1100Å^3^, Figure S58b), solvent exposed pocket is present, consistent with a cyclic substrate, and therefore lack of activity on **1**. The overall structure of the myxarylin-bound CorZ remains virtually unchanged compared to the CorZ-SAH complex (C_α_ RMSD of 0.30 Å, Figures 5b and S58c). The myxarylin occupies the large pocket, held in place mostly by hydrophobic interactions, such that the sulfur atom of SAH and the acceptor nitrogen atom in myxarylin are located in close proximity (∼3.9 Å, Figures 5c and S58c–e) with a near linear arrangement required for S_N_2 methyl transfer.^50^ The substrate orientation is such that N-terminal extension would clash with surrounding residues (Tyr5, Tyr12, Phe109, and Arg110; Figure 5c), rationalizing the lack of activity on **2**. In an effort to shift substrate preference of CorZ toward **2**, we generated two mutants: CorZ^Phe109Ala^ and CorZ^Phe109Gly/Arg110Gly^, to create additional space to accommodate the leader peptide (Figures S5c and S2a). We detected a mass shift (+ 14 Da; Figures 5d and S59) indicating methylation for **2**, affording **43**. Subsequently, incubation under basic conditions (*vide infra*) yielded a new peak with mass and retention time identical to myxarylin—further supporting *N*-methylation (Figure S60). We conclude methylation occurs after removal of the precursor peptide i.e., it is the free amine not the amide bond that is reactive species.

**Figure 5:**
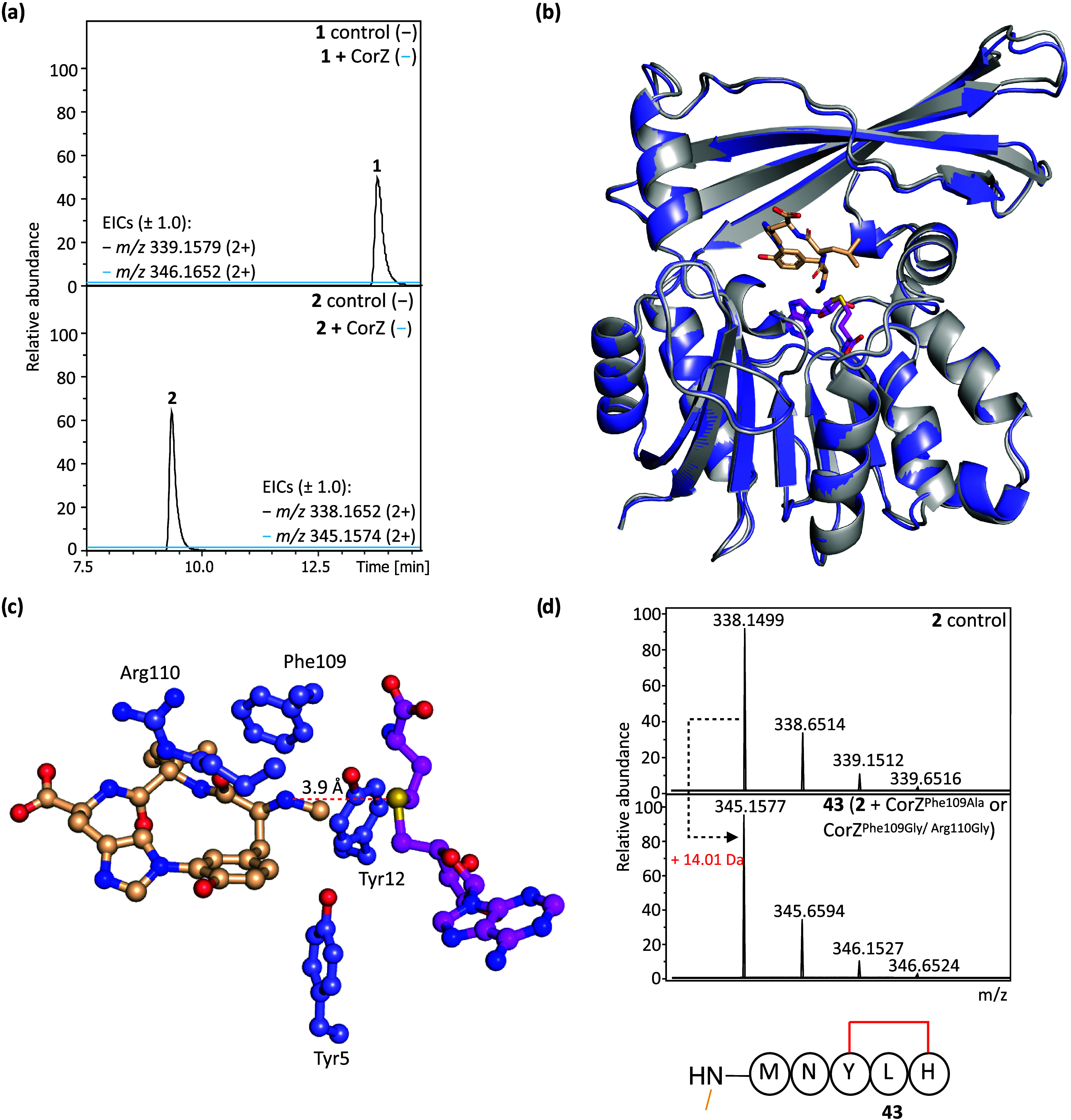
Structural and functional analysis of CorZ. (a) HPLC-ESI-MS analysis of **1** and **2** after incubation with CorZ in the presence of SAM. No methylated product was observed. (b) Superposition of the SAH bound CorZ (gray) structure with the CorZ/SAH/myxarylin complex (slate). Changes in the overall structure of the protein upon myxarylin binding are minimal (C_α_ RMSD of 0.30 Å over all non-hydrogen atoms). Myxarylin (wheat) and SAH (pink) are shown as sticks. (c) CorZ residues found to be in close proximity to the N-terminal residue of myxarylin. (d) HPLC-ESI-MS analysis of **2** after incubation with CorZ^Phe109Ala^ or CorZ^Phe109Gly/Arg110Gly^ in the presence of SAM. Detailed MS analyses of **1**, **2**, and **43** can be found in Figures S57 and S59.

### Timing of Leader Peptide Removal and the Identification of a Protease Involved in Biarylitides Maturation

Cyclic peptides are generally more stable at high pH conditions than their linear counterparts.^51–54^ We leveraged this property to obtain sufficient quantities of **44** by incubation at pH 9.0 overnight. The leader peptide was removed, with higher yields than that of **2** treated with Pronase E (Figures 6a and S61). Subsequent incubation of **44** with CorZ resulted in complete consumption of **44** (Figure S62), producing what we identified as myxarylin (mass shift of +14 Da; Figures 6a and S62), consistent with leader peptide removal followed by methylation.

**Figure 6:**
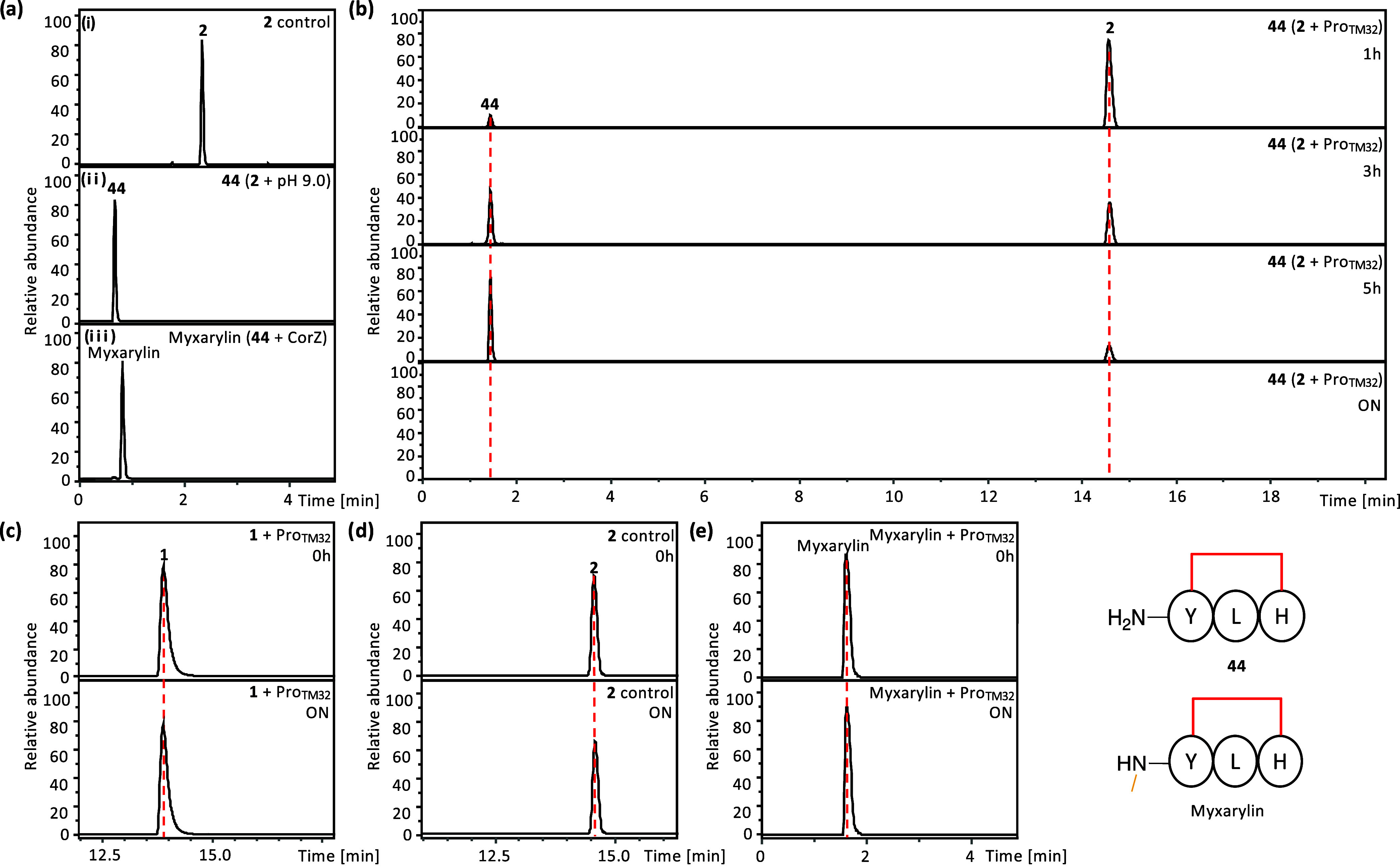
(a) *In vitro* reconstitution of myxarylin. Incubation of **44** in the presence of CorZ and SAM yielded myxarylin. (b, c) Time-course analysis of **44** and **1** in the presence of Pro_TM32_. (d) Stability analysis of **2** in the absence of Pro_TM32_. Minimal degradation was observed after overnight incubation under protease reaction conditions. (e) Effect of Pro_TM32_ on myxarylin. Myxarylin was found to be resistant to Pro_TM32_. Detailed MS analyses of **44**, **1**, **2**, and myxarylin can be found in Figures S61–S62 and S64–S67.

Inspection of the P450_BytO_ SSN reveals several biarylitide-like BGCs located next to a putative prolyl oligopeptide family serine protease (POP, pf00326; Figure S63).^55^ POPs belong to a distinct group of S9 serine proteases that generally hydrolyze polypeptides at the C-terminal of Pro residues.^56^ To our knowledge, only two bacterial RiPP POPs have been characterized: FlaP and MpcP, which are involved in the removal of the leader peptide of a class III lanthipeptide and clavusporins, respectively.^57–59^ Nonbacterial POPs have been identified in biosynthesis of cyclic RiPP toxins (amanitin and phalloidin) and omphalotin.^60–62^ We expressed and purified the putative protease from *Streptomyces sp*. TM32 (Pro_TM32_; Figures S2a and S63). Pro_TM32_ was incubated with **2**, and subsequent LC-HRMS analysis showed appearance of a species with a mass corresponding to **44** (Figures 6b and S64). Upon extended incubation, both **2** and **44** decrease, suggestive of degradation by the protease (Figure S6b). Compound **1** and modified compounds **34**–**35** were resistant to Pro_TM32_, suggesting a specificity of the protease activity toward myxarylin intermediates (Figures 6c and S65). We observed very little degradation of **2** in the absence of Pro_TM32_ (Figures 6d and S66). We speculated that methylation may confer resistance to the proteolytic cleavage and incubated myxarylin with Pro_TM32_, and monitoring the reaction over a time course showed this to be the case (Figures 6e and S67). These data point toward cooperativity between myxarylin biosynthetic machinery to ensure seamless biosynthesis. Attempts to copurify P450_BytO_/Corz/Pro_TM32_ or CorZ/Pro_TM32_ were unsuccessful, which implies any complexes are at best weak (data not shown). As all the biarylitides characterized to date lack a dedicated protease within their BGCs, the respective POP (or a different peptidase) may be encoded at different loci in the respective genomes. Such peptidases may fulfill other functions in their native hosts and are merely utilized by biarylitides for completing the precursor peptide modification.^6–10^

## Conclusion

The data presented here provide the first complete *in vitro* reconstitution of myxarylin, enabling us to dissect individual PTMs and thus propose a biosynthetic scheme (Figure 7).^11^ The first step, biaryl cross-linking, is catalyzed by P450_BytO_. This is followed by leader peptide removal by a protease. Further, nonspecific cleavage of the cyclized product is prevented by the action of CorZ, which installs N-terminal methyl group that renders the RiPP product resistant to proteolysis. The CorZ cocrystal structures allowed us to rationalize the substrate the selectivity of the methylase, demonstrated by the generation of CorZ mutants with extended substrate scope.

**Figure 7:**
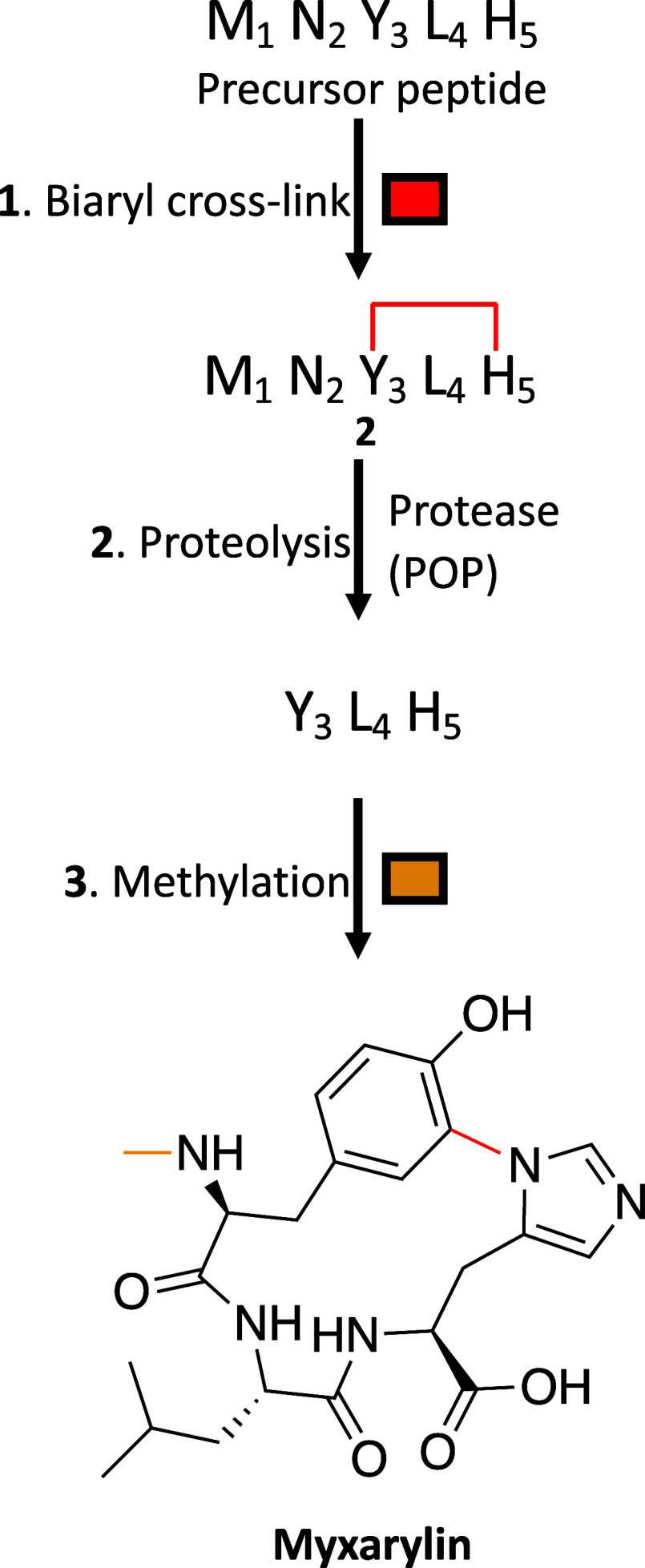
Proposed pathway to myxarylin.

A comprehensive analysis of the substrate tolerance of P450_BytO_ reveals remarkable substrate promiscuity, with one important element: a minimum two residue leader peptide. P450_BytO_ can also tolerate a folded protein domain as an N-terminal extension. Utilizing this broad substrate tolerance, we developed a modular precursor peptide design composed of tandem leader-core units, expanding the toolkit for RiPP pathway engineering.^39,40^ This RiPP engineering approach allowed the *in vitro* biosynthesis of thioamidated biarylitides. With standard coexpression plasmid system demonstrated to be suitable for the production of biarylitides (**34**–**36**; final yields ∼0.1 mg/L), the stage is set for the generation of biaryl derivatives. Combined with the modular leader peptide engineering strategy presented here, the platform has potential for combinatorial RiPP biosynthesis and generation of additional hybrid RiPP compounds. Engineered biarylitides allowed us to shed light on the substrate tolerance of ThoH/I and ThoC/D enzymes from the thioholgamide pathway.^43,44,46^ We extend the substrate scope of ThoH/I and demonstrate that the highly conserved terminal cysteine of the precursor peptide is not critical for its processivity. For ThoC/D, the size and/or composition of the substrate macrocycle appears to be crucial for activity.

A newly reported biarylitide P450 was shown to catalyze cross-linking between His-C2 and Tyr-O4.^12^ Additionally, the P450_Blt_ paralogue RufO was recently shown to perform nitration on a pentapeptide precursor peptide, which subsequently serves as a building block in the biosynthesis of rufomycin, an antituberculosis cyclic peptide.^63^ The expanding chemical space of biaryl and biarylitide-like P450s underscores the need for in-depth mechanistic studies and continued genome mining efforts to find new RiPP P450s, and other unrelated BGCs, with short precursor peptides.^8,10,64–69^ These investigations are ongoing and will complement RiPP engineering strategies to biosynthesize NPs with distinct chemical structures and bioactivities.

## Supplementary Material



## Notes

Experimental details, materials and methods, schematic representation of known biarylitides biosynthetic gene clusters; characterization of proteins used in this study; characterization of 42a; characterization of 44; biarylitide-like BGCs containing putative prolyl oligopeptidase; ^15^N HMBC spectrum of 34 in DMSO-*d*_6_ (0.2% TFA); sequence of genes of the *cor* BGC, ^1^H, ^13^C, and ^15^N shift comparison for the aromatic region of 34 and Myxarylin in DMSO-*d*_6_; and primers used in this study (PDF)
