# Supplementary material for: Myxarylin: Total In Vitro Biosynthesis, Expansion of Substrate Scope, and Bioengineered Thioamidated Biarylitides
Source: J Am Chem Soc. 2026 Feb 13;148(7):6970–80. doi: 10.1021/jacs.5c17257 (PMC12951456; doi:10.1021/jacs.5c17257)
Supplement: Supplementary file 1 [file ja5c17257_si_001.pdf]

Myxarylin: Total *In Vitro* Biosynthesis, Expansion of Substrate Scope, and Bioengineered Thioamidated Biarylitides

Asfandyar Sikandar, [a, b] Lana Vianey, [a] Kai Schließmann, [a] Qiyao Shen, [a] C. Logan Mackay, [b] F. P. Jake Haeckl, [a] Vlada B. Urlacher, [c] James H. Naismith, [b, d] and Rolf Müller [a, f, *]

a Helmholtz Institute for Pharmaceutical Research Saarland (HIPS), Helmholtz Center for Infection Research (HZI), Saarbrücken, 66123, Germany

b The Rosalind Franklin Institute, Harwell Campus, Didcot, OX11 0QX, U.K.

c Institute of Biochemistry, Heinrich-Heine University Düsseldorf, Düsseldorf, 40225, Germany.

d University of Oxford, Division of Structural Biology, Oxford, OX3 7BN, U.K.

f Department of Pharmacy, Pharmaceutical Biotechnology, Saarland University, Saarbrücken, 66123, Germany.

Table of Contents

Experimental Procedures 4
- Cloning, Expression and Purification 4
- Peptide synthesis 5
- Structural modelling 5
- Biochemical assay 5
- LC-MS and MS/MS analysis 5
- Purification of 34 7
- NMR measurement 7
- Crystallization and structure determination 7
- Ligand binding assay 7

Supplementary Figures 9
- Figure S1 9
- Figure S2. 9
- Figure S3 10
- Figure S4. 10
- Figure S5. 11
- Figure S6. 11
- Figure S7. 12
- Figure S8. 12
- Figure S9. 13
- Figure S10. 14
- Figure S11. 14
- Figure S12. 15
- Figure S13 15
- Figure S14. 16
- Figure S15. 17
- Figure S16. 17
- Figure S17. 18
- Figure S18 18
- Figure S19 19
- Figure S20 19
- Figure S21 20
- Figure S22. 20
- Figure S23 21
- Figure S24 21
- Figure S25. 22
- Figure S26. 22
- Figure S27 23
- Figure S28 24
- Figure S29 24
- Figure S30 25
- Figure S31 25
- Figure S32 26
- Figure S33 26
- Figure S34 27

Figure S35 27
Figure S36 28
Figure S37 28
Figure S38 29
Figure S39 29
Figure S40 30
Figure S41 30
Figure S42 31
Figure S43 31
Figure S44 32
Figure S45 32
Figure S46 33
Figure S47 34
Figure S48 35
Figure S49 35
Figure S50 36
Figure S51 36
Figure S52 36
Figure S53 37
Figure S54 37
Figure S55 37
Figure S56 38
Figure S57 38
Figure S58 39
Figure S59 40
Figure S60 40
Figure S61 41
Figure S62 42
Figure S63 43
Figure S64 44
Figure S65 44
Figure S66 45
Figure S67 45

NMR Spectra and Figures 46
Figure S68 46
Figure S69 46
Figure S70 47
Figure S71 47
Figure S72 48
Figure S73 48
Figure S74 49

Supplementary Tables 50
Table S1 50
Table S2 52
Table S4 55
Table S5 56
Table S6 57

References 59

Experimental Procedures

Cloning, Expression and Purification

Genes were amplified using the corresponding primers (Table S5). All proteins except for $P450_{BytO}^{Hypermodified}$ and Pro_{TM32} (Figure S47 and Table S1; GenScript) were cloned from genomic DNA isolated from *Pyxidicoccus fallax* An d48 and *Corallococcus coralloides* Mcy6431 into the respective expression plasmids using Gibson Assembly HiFi master mix (NEB). Hybrid precursor peptide (**40**) was cloned from $ThoA^{A\ (8\text{-}12)}$ plasmid.[1] Site-directed mutagenesis was carried out by overlap extension PCR. The resulting plasmids were verified by enzyme restriction digestion and DNA sequencing before being transformed into C43 (DE3) or BL21 (DE3).

A single colony was transferred into LB liquid medium containing appropriate antibiotic(s) to grow an overnight culture. This culture was then used to inoculate (1 to 100) fresh TB medium supplemented with antibiotics and grown at 37 °C until the optical density (OD_{600}) reached 1.0. The cultures were then cooled on ice for 15 min, followed by induction of protein expression by addition of 0.5 mM IPTG at a shaker temperature of 25 °C and left shaking overnight (180 rpm). In the case of $P450_{BytO}$ (and mutants) the cultures were further supplemented with 5-Aminolevulinic acid (final concentration: 1mM). Cell were harvested by centrifugation and cell pellet frozen at – 80 °C. Frozen cell pellets were resuspended in lysis buffer (20 mM Tris pH 8.0, 500 mM NaCl, 20 mM imidazole, pH 8.0) supplemented with 0.4 mg DNAse per gram of wet pellet and cOmplete EDTA-free protease inhibitor tablets (Roche). Pro_{TM32} was purified in the absence of protease inhibitor tablets. The cell suspension was lysed via CF1 cell disruptor (30 kpsi, Constant Systems Ltd.) and cell debris removed by centrifugation (40,000 x g, 4 °C, 20 min). The supernatant was loaded onto a 5 mL His60 (Takara Bio) column preequilibrated with lysis buffer and the loaded column was washed extensively (20 column volumes) with lysis buffer. The bound protein was eluted in one step using lysis buffer supplemented with 250 mM imidazole. Fractions containing protein were directly loaded onto a gel filtration column (HiLoad 16/600 Superdex 200 pg, GE Healthcare) preequilibrated in gel filtration buffer ($P450_{BytO}$ or mutants and Pro_{TM32}: 50 mM Tris pH 7.5, 200 mM NaCl; and CorZ: 20 mM Tris pH 8.0, 150 mM NaCl ,1mM TCEP). The fractions of the highest purity and RZ value (absorption ratio $A_{418}/A_{280} > 0.6$ for $P450_{BytO}$ and mutants) were pooled together and concentrated to 5 - 8 mg mL^{-1} using either a Bradford assay or Nanodrop UV-Vis spectrophotometer. The concentration of cytochrome P450s were further estimated using the CO-difference spectral assay as described previously with $\varepsilon_{450\text{–}490} = 91\ mM^{-1}\ cm^{-1}$.[2]

For co-expression studies the purification protocol was slightly modified. The cell pellet was resuspended in lysis buffer supplemented with 6 M urea. The sample was sonicated and the insoluble material was removed by centrifugation (40,000 x g, 30 min). The supernatant was passed through a 0.45 µm filter and then loaded onto a 5mL His60 (Takara Bio) column. The column was washed extensively with lysis buffer containing 6 M urea and the peptide was eluted with lysis buffer supplemented with 250 mM imidazole. The fractions were desalted into lysis buffer containing 1 M urea. After overnight treatment with TEV protease at 4 °C the peptides were reapplied to 5 mL His60 (Takara Bio) column. The flow through was collected and loaded onto a desalting column to change the buffer to gel filtration buffer.

Redox partners FdR (*E. coli*) and YkuN (*B. substilis*) or PdR and Pdx (*P. putida*) were expressed and purified as previously described.[3, 4] For spectroscopic determination of YkuN ($\varepsilon_{461} = 10.01\ mM^{-1}\ cm^{-1}$), FdR ($\varepsilon_{456} = 7.1\ mM^{-}$

^{1} cm^{-1}), Pdr (ε_{454} = 10.9 mM^{-1} cm^{-1}) and Pdx (ε_{455} = 5.9 mM^{-1} cm^{-1}) concentrations published extinctions coefficients were used. [4-7] Spinach ferredoxin (Fdx), ferredoxin-$NADP^+$ reductase (Fdr, Simga-Aldrich) and Pronase E were purchased from Sigma-Aldrich and were used without further purification. ThoH/I and ThoC/D were purified as previously described.[1]

Peptide synthesis

The solid-phase synthesis of the peptides (**1** – **32** and **37** – **39**) was carried out by GenScript and were used without further purification.

Structural modelling

Protein structure predictions were performed using AlphaFold server.[8]

Biochemical assay

Generally, peptide (50 – 100 µM) was incubated with protein ($P450_{BytO}$ or its variants (2.5 µM); Pro_{TM32} (5 µM): Pronase E (2.5 µg); or CorZ (5 µM)) in reaction buffer (Table S5) at 30 °C for 4 –18 hr. Reactions were stopped and enzymes were precipitated by addition of one volume of MeOH before being analyzed by LC-MS. Large scale $P450_{BytO}$ cyclization reactions were carried out in 1 ml scale using the above-mentioned reaction set-up. After MeOH precipitation the supernatant was filtered through a 0.22 µm filter, lyophilized overnight and then resuspended in reaction buffer or 50 mM Tris pH 9.0.

LC-MS and MS/MS analysis

LC-MS measurements were performed on the following systems.

1) Peptides **1** – **24** and **31** – **32**: Dionex Ultimate 3000 RSLC system using a BEH C18, 50 × 2.1 mm^2, 1.7-µm particle diameter (dp) column equipped with a C18 precolumn (Waters), coupled to a maXis 4G high-resolution time of flight (HR-ToF) mass spectrometer (Bruker Daltonic, Germany) using an Apollo electrospray ionization (ESI) source. Separation of 3 µL samples was achieved by a linear gradient from H_2O plus 0.1% formic acid (FA) (Solvent A) to acetonitrile (ACN) plus 0.1% FA (solvent B). Gradient B: 0 – 0.5 min (2%), 0.5 – 10.0 min (2 – 75%), 10.0 – 13.0 min (75%), 13.0 – 18.0 min (2%). The following condition were used; capillary voltage 4,000 V, temperature 200 °C, dry gas flow rate 5 L / min and nebulizer 14.5 psi. Data was recorded in the mass range from 150 to 2,500 m / z and the calibration of the maXis 4G spectrometer was achieved with sodium formate clusters before every injection to avoid mass drift. LC-MS/MS fragmentation was performed using an automatic precursor peptide selection mode.
2) Peptides **1** – **24** and **31** – **32:** Vanquish Flex UHPLC equipped with Waters (Eschborn, Germany) BEH C_{18} column (100 × 2.1 mm, 1.7 µm) equipped with a Waters VanGuard BEH C_{18} 1.7 µm guard column. Separation of 2 µL sample was achieved by a linear gradient from (A) H_2O + 0.1% FA to (B) ACN + 0.1% FA at a flow rate of 600 µL/min and 45 °C. The gradient was initiated by a 0.5 min isocratic

step at 5 % B, followed by a slow increase to 40 % B in 16 min and a steep increase to 95 % in 2 min to end with a 2 min step at 95 % B before re-equilibration with initial conditions. UV-vis spectra were recorded by a DAD in the range from 200 to 600 nm. The timsTOF fleX was operated in positive ESI mode, with 1.0 bar nebulizer pressure, 5.0 L/min dry gas, 200°C dry heater, 4000 V capillary voltage, 500 V end plate offset, 500 Vpp funnel 1 RF, 250 Vpp funnel 2 RF, 80 V deflection delta, 5 eV ion energy, 10 eV collision energy, 1100 Vpp collision RF, 5 µs pre-pulse storage, 65 µs transfer time. TIMS delta values were set to -20 V (delta 1), -120 V (delta 2), 80 V (delta 3), 100 V (delta 4), 0 V (delta 5), and 100 V (delta 6). The 1/K0 (inverse reduced ion mobility) range was set from 0.55 Vs/cm2 to 1.87 Vs/cm^2, the mass range was *m/z* 100-2000. MS2 spectra were acquired using the PASEF DDA mode with a collision energy gradient based on ion mobility: Starting at 25 eV for 0.55 Vs/cm^2 (1/K0) to 35 eV at 1.2 Vs/cm^2 to 40 eV at 1.5 Vs/cm^2 to 60 eV for 1.87 Vs/cm^2. Ion charge control (ICC) was enabled and set to 7.5 Mio. counts. The analysis accumulation and ramp time was set at 100 ms with a spectra rate of 9.43 Hz and a total cycle of 0.32 sec was also selected resulting in one full TIMS-MS scan and two PASEF MS/MS scans. Precursor ions were actively excluded for 0.1 min and were reconsidered if the intensity was 2.0-fold higher than the previous selection with a target intensity of 4000 and an intensity threshold of 100. TIMS dimension was calibrated linearly using 4 selected ions from ESI Low Concentration Tuning Mix (Agilent Technologies, USA) [*m/z*, $1/k_0$: (301.998139, 0.6678 Vs cm^{-2}), (601.979077, 0.8782 Vs cm−2)] in negative mode and [*m/z*, $1/k_0$: (322.048121, 0.7363 Vs cm^{-2}), (622.028960, 0.9915 Vs cm^{-2}), (922.0098, 0.9915 Vs cm^{-2}), (622.028960, 0.9915 Vs cm^{-2})] in positive mode. The mobility for mobility calibration was taken from the CCS compendium.[9] Calibration was done automatically before every LC-MS run by injection of a basic sodium formate solution through a filled 20 µL loop switched into the LC flow at the beginning of each run.

3) Peptides **40 – 42**: Dionex Ultimate 3000 RSLC system using an Aeris Widepore XB-c8, 150 x 2.1 mm, 3.6 µm particle diameter (dp) column (Phenomenex, USA), coupled to a maXis 4G high-resolution time of flight (HR-ToF) mass spectrometer (Bruker Daltonic, Germany) using an Apollo electrospray ionization (ESI) source. Separation of 3 µL samples was achieved by a linear gradient from H_2O plus 0.1% formic acid (FA) (Solvent A) to acetonitrile (ACN) plus 0.1% FA (solvent B). Gradient B: 0 – 0.5 min (2%), 0.5 – 10.0 min (2 – 75%), 10.0 – 13.0 min (75%), 13.0 – 18.0 min (2%). The following condition were used; capillary voltage 4,000 V, temperature 200 °C, dry gas flow rate 5 L / min and nebulizer 14.5 psi. Data was recorded in the mass range from 150 to 2,500 m / z and the calibration of the maXis 4G spectrometer was achieved with sodium formate clusters before every injection to avoid mass drift. LC-MS/MS fragmentation spectra were recorded using scheduled precursor lists (SPLs). Separation was achieved using conditions identical to LC-MS. SPL entries and parameter were set to fragment only the ions of interest. SPL tolerance parameters for precursor ion selection were 0.5 min and 0.5 m / z. The collision-induced dissociation energy was ramped from 35 eV for 500 m / z to 50 eV for 1,000 m / z.

4) Peptides **25** – **30** and **37** – **39**: Acquity UPLC (Waters) using an BEH C18, 50 × 2.1 mm2 , 1.7-µm particle diameter (dp) column (Waters), coupled to a 7 T prototype SolariX TIMS FT-ICR MS (Bruker Daltonics, Germany) with an electrospray ionization (ESI) source in positive mode, with nebulizer gas flow set to 1.0 bar with drying gas at 4 L min-1 at 200 °C. Separation 7 of 1-5 µL samples was achieved by a linear

gradient from H2O plus 0.1% formic acid (FA) (Solvent A) to acetonitrile (ACN) plus 0.1% FA (solvent B). Gradient B: 0 – 0.5 min (2%), 0.5 – 10.0 min (2 – 50%), 10.0 – 13.0 min (50 – 75%), 13.0 – 18.0 min (2%). Each spectra was the sum of 2 2 Mword acquisitions between *m/z* 150 and 3000.

Purification of **34**

The expression was scaled to 60 litres of media. Standard co-expression and purification protocol mentioned earlier was followed. After TEV cleavage the peptide was reapplied to 5 ml His60 (Takara Bio) column and the flow through was collected. Further purification was performed using preparative reverse-phase (RP) HPLC-MS with an AutoPurification system (Waters Corp.) equipped with a DAD detector module and a single-quad MS spectrometer using a XBridge BEH C18 column (130Å, 5µm, 19mm x 150mm; Waters Corps) at 25 mL/min flow rate. Fractions were collected by time-based collection and their purity was verified by analytical LC-MS. The conditions for the chromatographic system were as follows: solvent A: H_2O + 0.1% FA, solvent B: ACN + 0.1% FA; gradient: 0-1 min: 5% B, 1-6 min: 5-14% B, 6-7 min: 95% B, 7-10 min: 95-5% B.

NMR measurement

NMR data was recorded on an AVANCE III 700 MHz NMR (^{1}H at 700 MHz, ^{13}C at 175 MHz, ^{15}N at 71 MHz) and an UltraShield 500 MHz (^{1}H at 500 MHz, ^{13}C at 125 MHz) equipped with a 5 mm inverse TCI cryoprobe (Bruker). Shift values (δ) were calculated in ppm, and coupling constants (J) were calculated in Hz. Spectra were recorded in DMSO-d_6 and adjusted to the solvent signals (δ_H 2.5, δ_C 39.51). Measurements were conducted in 5 mm Shigemi tubes (Shigemi Inc.). For the two-dimensional experiments HMBC, HSQC and gCOSY standard pulse programs were used. HMBC experiments were optimized for $^{2,3}J_{C\text{-}H}$ = 8 Hz, and HSQC were optimized for $^1J_{C\text{-}H}$ = 145 Hz. ^{15}N-HMBC experiments were optimized for $^{2,3}J_{C\text{-}H}$ = 5 Hz and referenced to ammonia (= 0 ppm).

Crystallization and structure determination

Crystals of CroZ / SAH and CorZ / SAH / myxarylin were obtained using the sitting-drop vapor diffusion method at 18 °C in 15 – 34% precipitant mix 2 (40% v/v ethylene glycol; 20% w/v PEG 8000), 0.09M halogens (0.3M sodium fluoride; 0.3M sodium bromide; 0.3M sodium iodide) and buffer system 3 pH 8 - 9 (Tris (base); Bicine). For complex crystallization, protein (8 mg / mL) was incubated with excess myxarylin (1 mM) on ice overnight. Crystals were cryoprotected in mother liquor supplemented with 30% glycerol. The diffraction data was collected from a single crystal at 100 K at Petra III (Beamline: P11, DESY)[10], processed using Xia2[11] or XDS[12], and the structure was determined using PHASER molecular replacement[13] using AlphaFold model generated using Colab notebook.[8] The structure was manually rebuilt in COOT[14], refined using PHENIX Refine[13] and validated using MolProbity.[15] The images presented were created using PyMOL (Schrödinger) and LigPlot$^+$.[16]

Ligand binding assay

The dissociation constants (K_d values) were determined on a CLARIOstar*Plus* microplate reader (BMG Labtech) using difference spectroscopy as described elsewhere with slight modifications.[17] Samples containing P450s (3

μM) were incubated with different concentrations of **1** in binding buffer (50 mM Tris-HCl, pH 8.0). Amplitudes of the different spectra of three individual experiments were extrapolated ($\Delta A = A_{max} - A_{min}$) and plotted against ligand concentration. Curve fitting of the data points was performed using GraphPad Prism 10 by applying one-site binding model $Y = B_{max}*X/(K_d+X)$ + NS*X + Background (Y = delta Abs, B_{max} = the maximum specific binding, K_d = equilibrium dissociation constant, NS = slope of nonspecific binding, Background = nonspecific binding with no added ligand).

Supplementary Figures

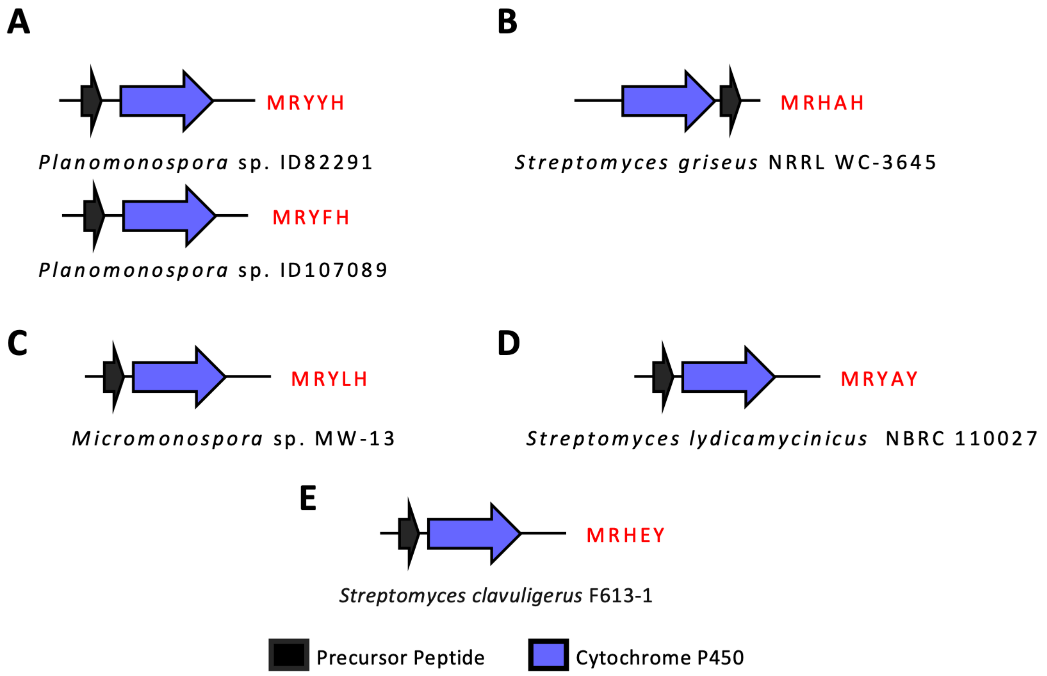

Figure S1. Schematic representation of known biarylitides biosynthetic gene clusters (BGCs). After ribosomal translation, the precursor peptide (sequence: highlighted red) undergoes cyclisation catalysed by a cytochrome P450 enzyme to form biarylitide YYH / YFH (A), gristide 834 (B), ΔN-1_{linked} (C) SlyP product (D) and ShyB product (E), respectively. The protease(s) responsible for the removal of the leader peptide (first two residues) is currently unknown.[18] Unlike other biarylitides, YYH and YFH are N-acetylated by a yet unidentified acetylation mechanism.[19]

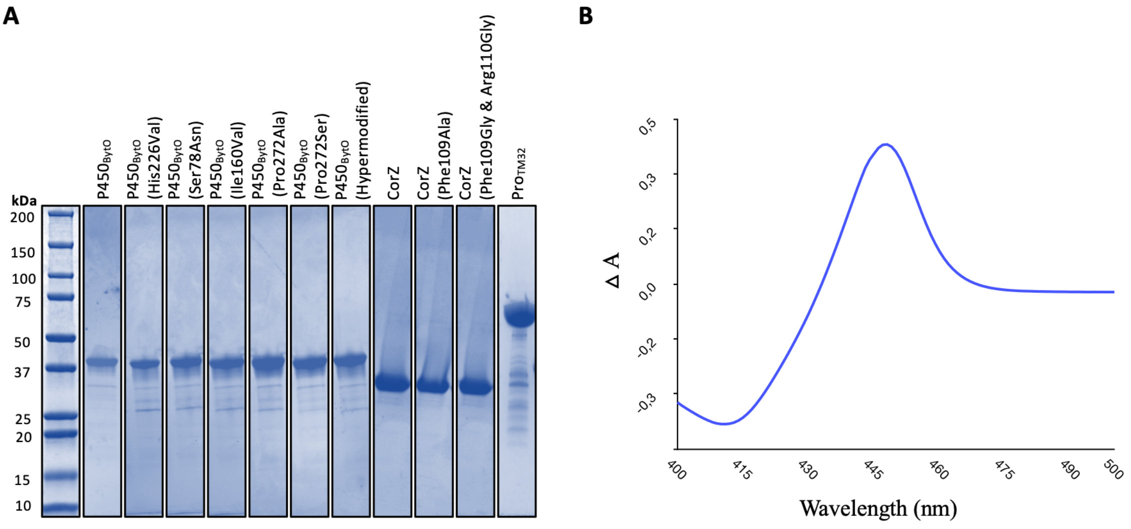

Figure S2. Characterization of proteins used in this study. **A)** SDS-PAGE analysis of $P450_{BytO}$ and its engineered variants, CorZ and its mutants, and Pro_{TM32}. Sequence alignment of $P450_{BytO}$ and hypermodified $P450_{BytO}$ can be found in Figure S47. Protein purity was assessed visually using Coomassie staining. **B)** The dithionite reduced CO-difference spectrum of $P450_{BytO}$ (blue line) shows the typical peak maximum at 448 nm for the Fe^{II}-CO complex.

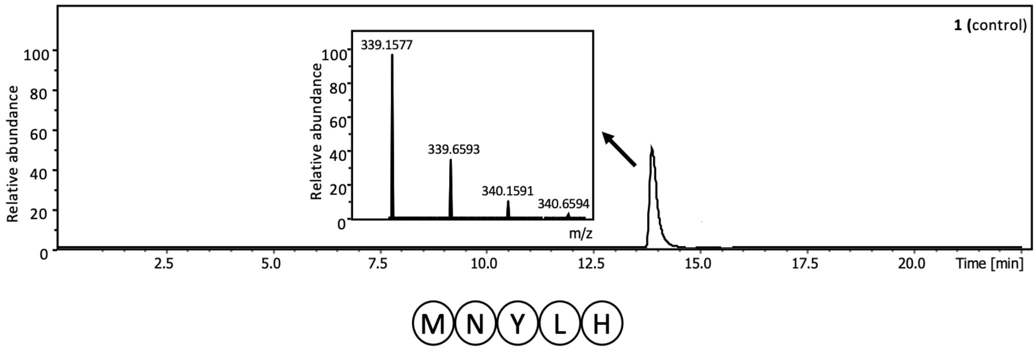

1

Calculated Mass: 339.1574 (2+)
Observed Mass: 339.1577 (2+)
Relative deviation (ppm): 0.88

Figure S3. Characterization of precursor peptide (**1**). HPLC-ESI-MS analysis of **1** is shown.

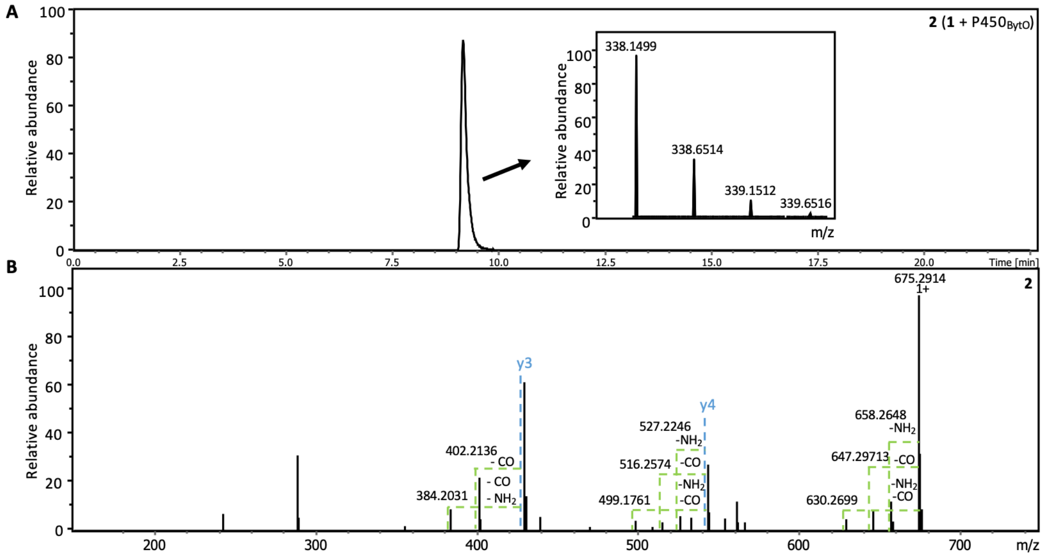

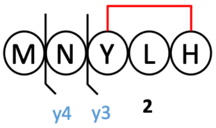

2

Calculated Mass: 338.1496 (2+)
Observed Mass: 338.1499 (2+)
Relative deviation (ppm): 0.89

	Observed Mass m/z	Expected Mass m/z	Relative deviation (ppm)
y3	430.2085	430.2079	1.40
y4	544.2517	544.2509	1.47

Figure S4. Characterization of **2**. **A)** HPLC-ESI-MS analysis of **2**. $P450_{BytO}$ was incubated in the presence of different redox partners (not shown). Only FdR/YkuN pair resulted in the formation of **2 (**biaryl linkage, red bracket). **B)** HR-MS/MS analysis is shown and the y ions are listed in the table and marked in the spectrum. No conversion was observed with other redox partners tested (Table S5).

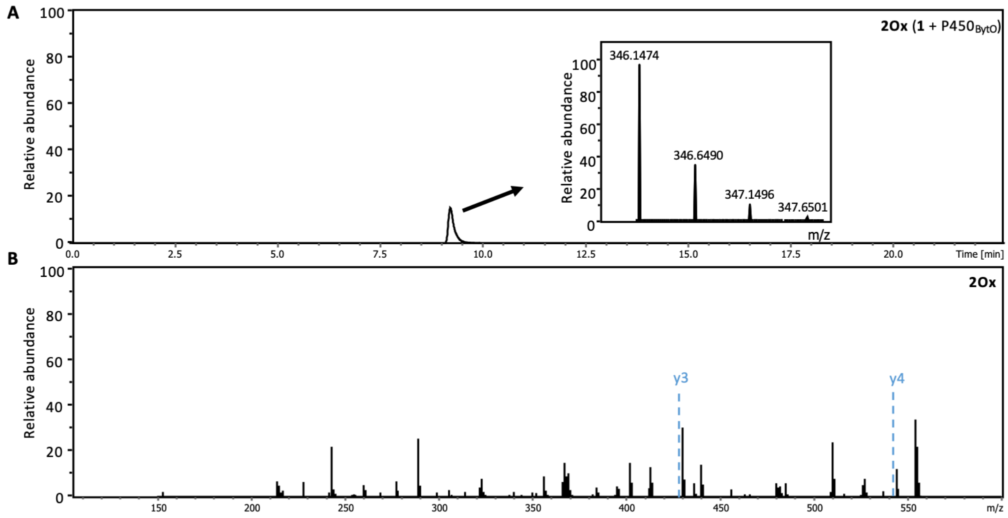

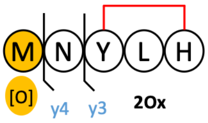

Calculated Mass: 346.1470 (2+)
Observed Mass: 346.1474 (2+)
Relative deviation (ppm): 1.16

	Observed Mass m/z	Expected Mass m/z	Relative deviation (ppm)
y3	430.2081	430.2079	0.46
y4	544.2509	544.2509	0

Figure S5. Characterization of oxidised **2**. **A)** HPLC-ESI-MS analysis of oxidized **2** (**2Ox**; biaryl linkage, red bracket). In comparison to **2**, the amount of **2Ox** observed is significantly less (see Figure S3). **B)** HR-MS/MS analysis is shown and the y ions are listed in the table and marked in the spectrum.

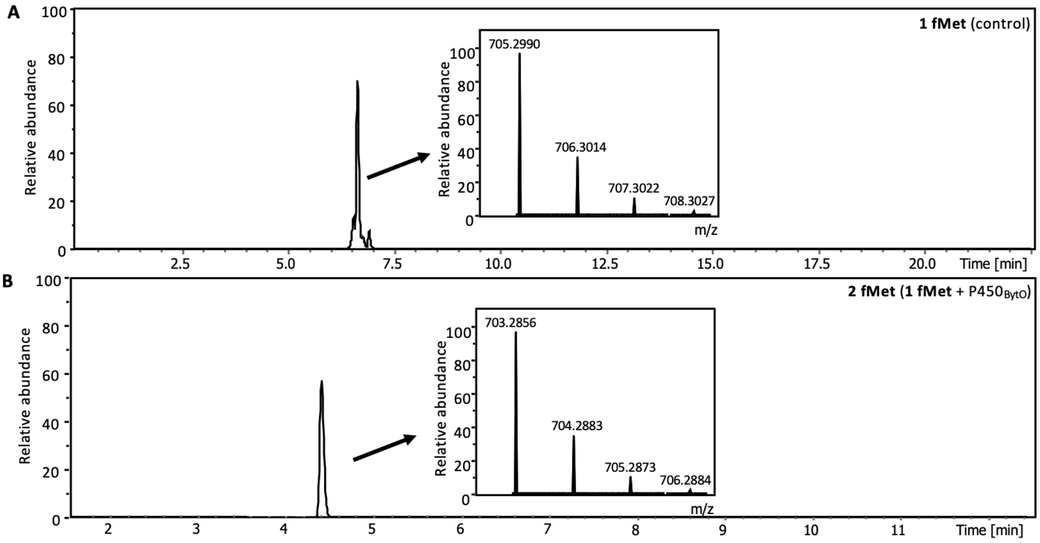

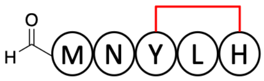

2 fMet

Calculated Mass: 703.2868 (1+)
Observed Mass: 703.2856 (1+)
Relative deviation (ppm): -1.71

Figure S6. Characterization of N-formylmethionine **1**. **A)** HPLC-ESI-MS analysis of N-formylmethionine1 (**1 fMet**; control). **B)** EIC of **2 fMet**. Similar to **1**, complete consumption of **1 fMet** was observed.

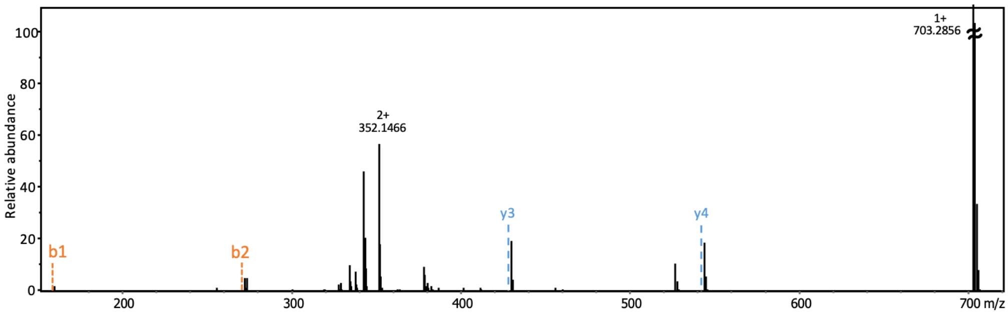

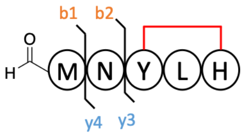

2 fMet

	Observed Mass m/z	Expected Mass m/z	Relative deviation (ppm)
b1	160.0423	160.0432	-5.62
b2	274.0853	274.0862	-3.28
y3	430.2080	430.2079	0.23
y4	544.2508	544.2509	-0.18

Figure S7. Characterization of **2 fMet**. HR-MS/MS analysis is shown and the b and y ions are listed in the table and marked in the spectrum**.**

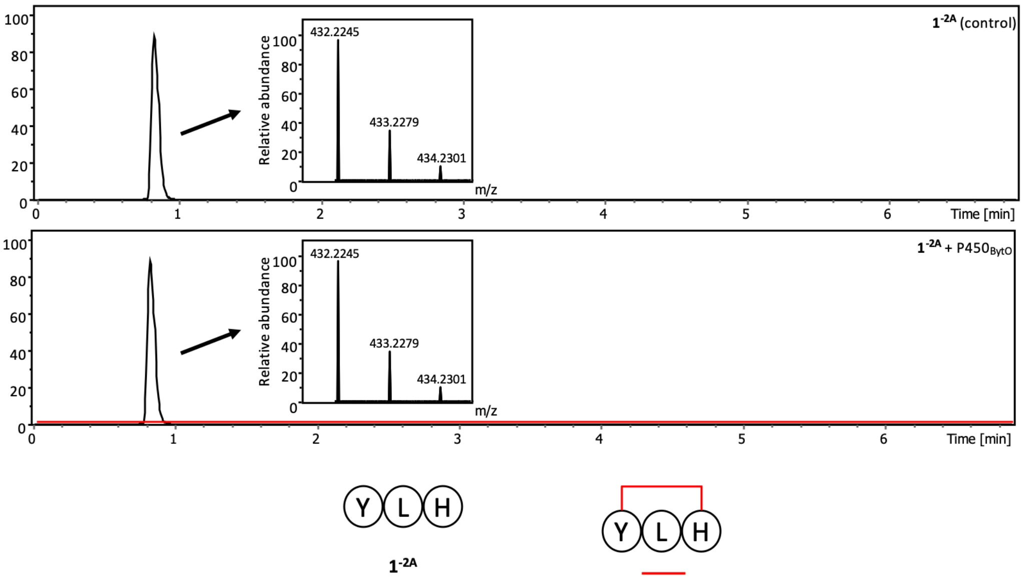

1^{-2A}

Calculated Mass: 432.2242 (1+)
Observed Mass: 432.2245 (1+)
Relative deviation (ppm): 0.70

Calculated Mass: 430.2085 (1+)
Observed Mass: not observed

Figure S8. Characterization of **1^{-2A}**. HPLC-ESI-MS analysis of **1^{-2A}** (control; top) and upon incubation with P450$_{BytO}$ is shown (bottom). Even upon extended incubation (> 24 hr) no product was observed (EIC: 430.2085 ±0.2; red line).

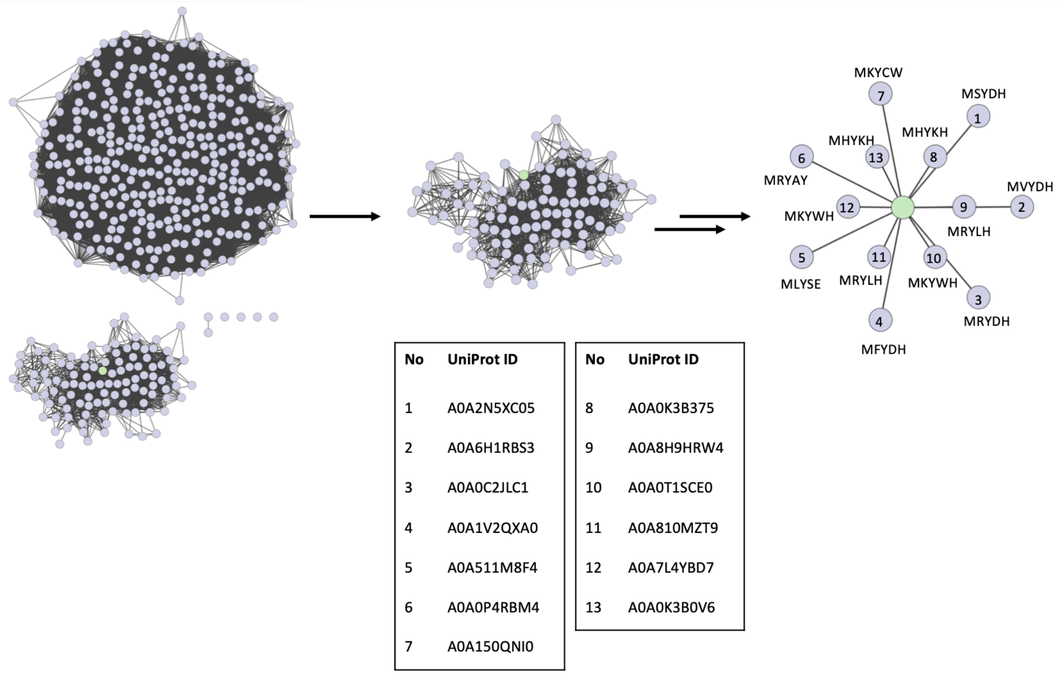

No	UniProt ID	No	UniProt ID
1	A0A2N5XC05	8	A0A0K3B375
2	A0A6H1RBS3	9	A0A8H9HRW4
3	A0A0C2JLC1	10	A0A0T1SCE0
4	A0A1V2QXA0	11	A0A810MZT9
5	A0A511M8F4	12	A0A7L4YBD7
6	A0A0P4RBM4	13	A0A0K3B0V6
7	A0A150QNI0		

Figure S9. Sequence similarity network (SSN) of $P450_{BytO}$. The network was generated using the EFI tool with default settings and visualized in cytoscape v3.9.1.[20] An alignment score of 71 was applied to separate the clusters. Subsequently, daughter networks were generated from the represented node (RepNod 40, shown on the left) by selecting the first neighbours of $P450_{BytO}$ (light green node). The uniport IDs of cytochrome P450 enzymes are shown at the bottom. The BGCs associated with these cytochrome P450s were analysed manually and / or via genome neighbourhood diagram (GND) to locate the putative precursor peptides (sequence shown next to the associated nodes).

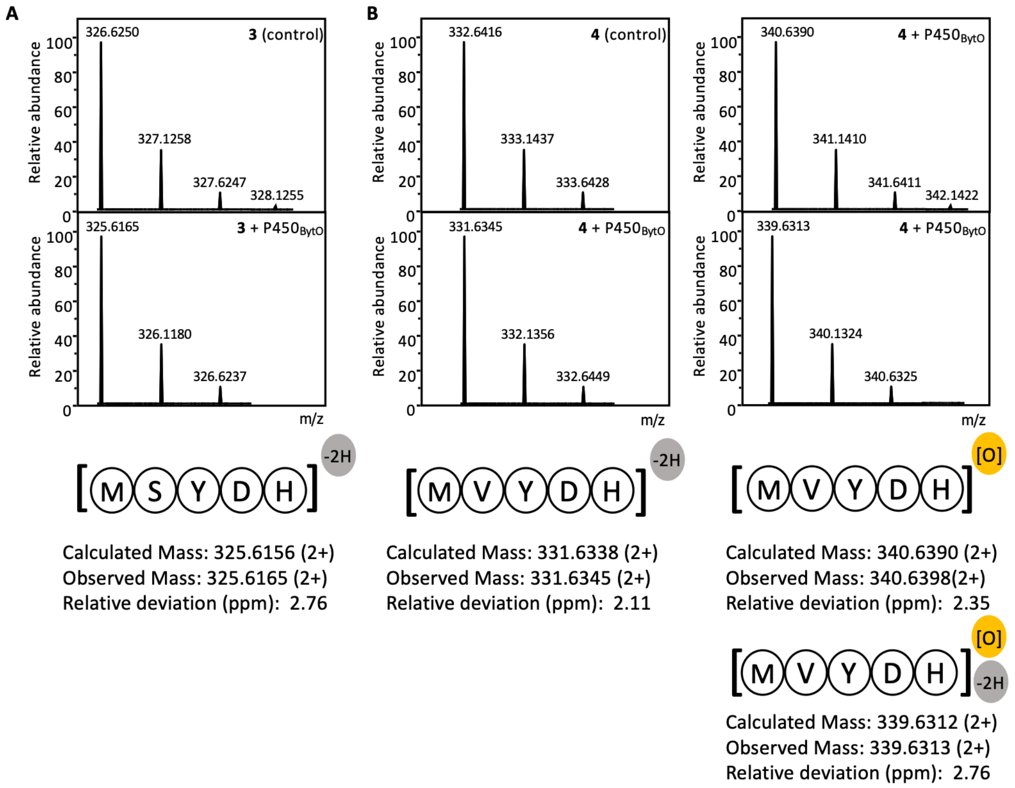

Figure S10. Characterization of **3** and **4**. **A** and **B)** HPLC-ESI-MS characterization of **3** and **4** (control) and after incubation with P450$_{BytO}$ are shown. Observed and calculated mass values are shown and the modifications installed by P450$_{BytO}$ are highlighted.

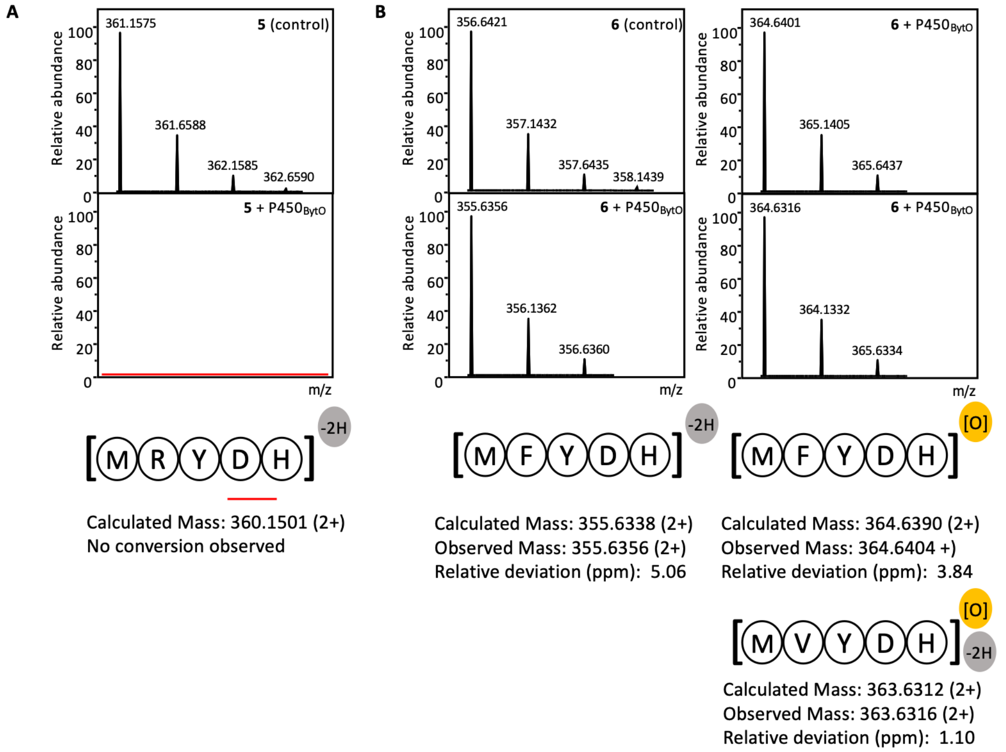

Figure S11. Characterization of **5** and **6**. **A** and **B)** HPLC-ESI-MS characterization of **5** and **6** (control) and after incubation with P450$_{BytO}$ are shown. Observed and calculated mass values are shown and the modifications installed by P450$_{BytO}$ are highlighted.

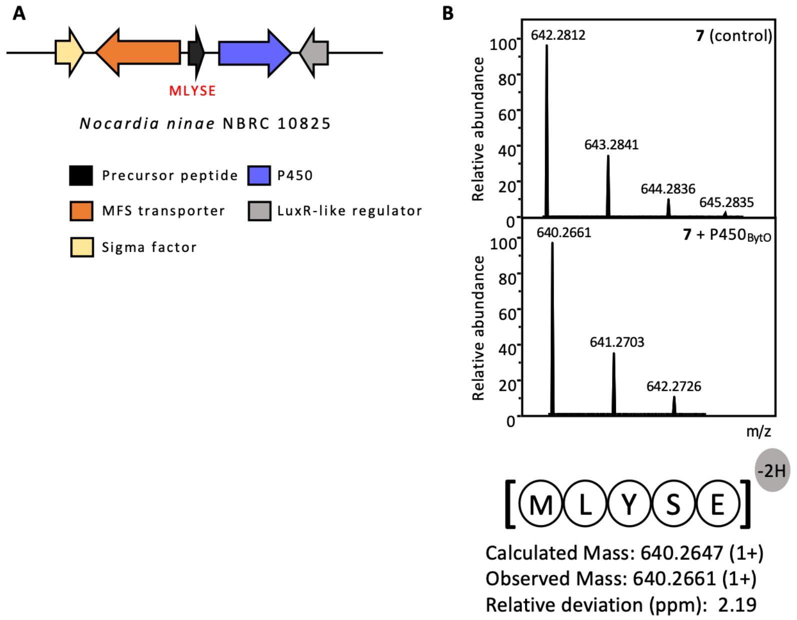

Figure S12. Characterization of BGC containing **7**. **A)** Simplified diagram of biarylitide-like BGC found in Nocardia ninae NBRC 10825. The putative precursor peptide sequence is shown (red). **B)** HPLC-ESI-MS characterization of **7** (control) and after incubation with $P450_{BytO}$ are shown. Observed and calculated mass values are shown and the modification installed by $P450_{BytO}$ are highlighted.

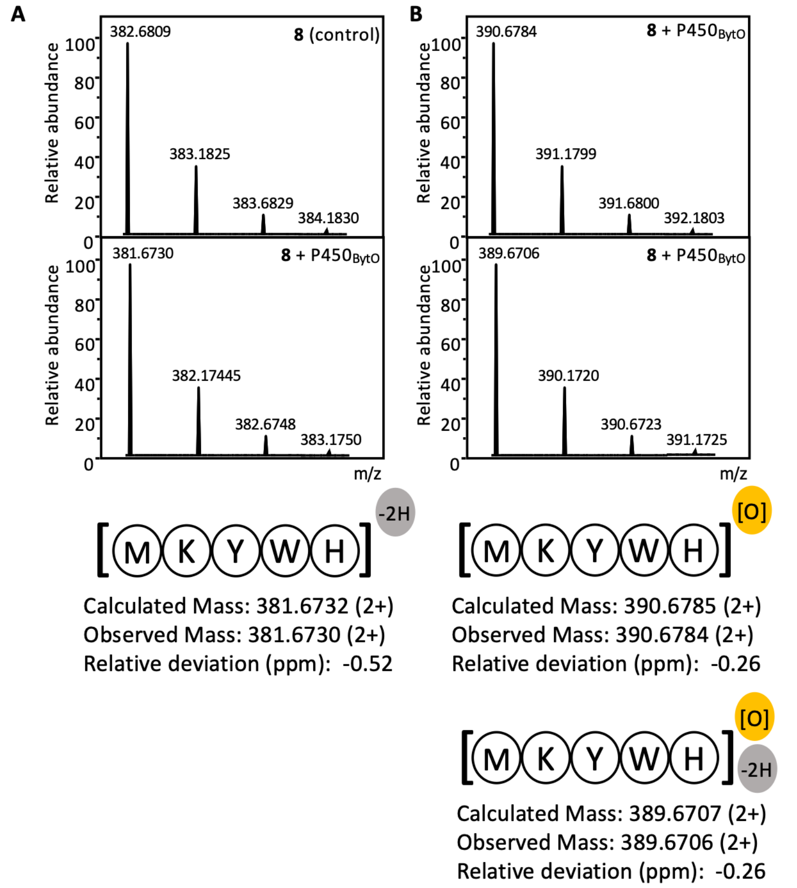

Figure S13. Characterization of **8**. **A** and **B)** HPLC-ESI-MS characterization of **8** (control) and after incubation with $P450_{BytO}$ are shown. Observed and calculated mass values are shown and the modifications installed by $P450_{BytO}$ are highlighted.

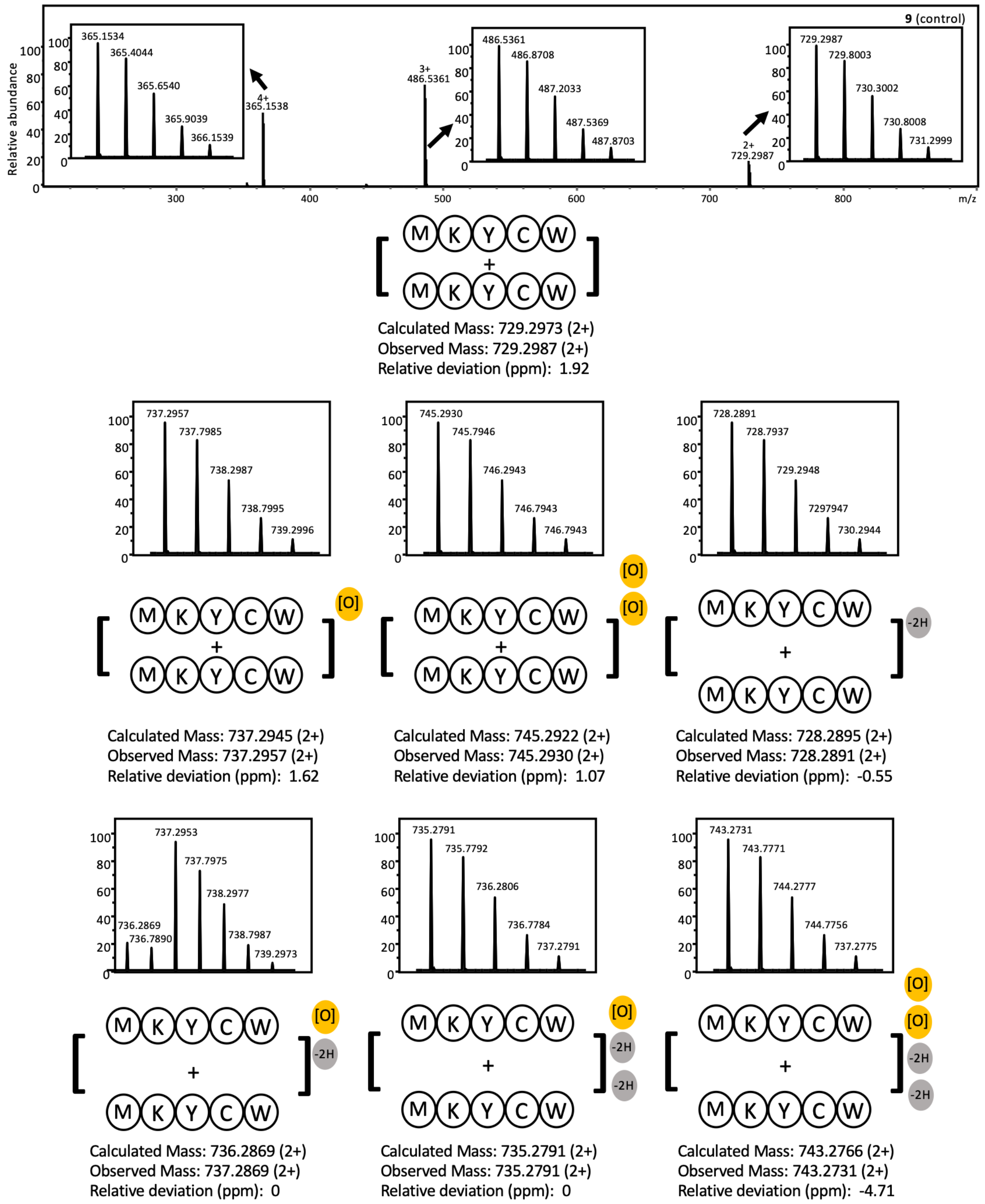

Figure S14. Characterization of **9**. HPLC-ESI-MS characterization of **9** (control) and after incubation with $P450_{BytO}$ are shown. The observed substrate dimerization is likely due to cysteine disulfide bond formation. Observed and calculated mass values are shown and the modifications installed by $P450_{BytO}$ are highlighted.

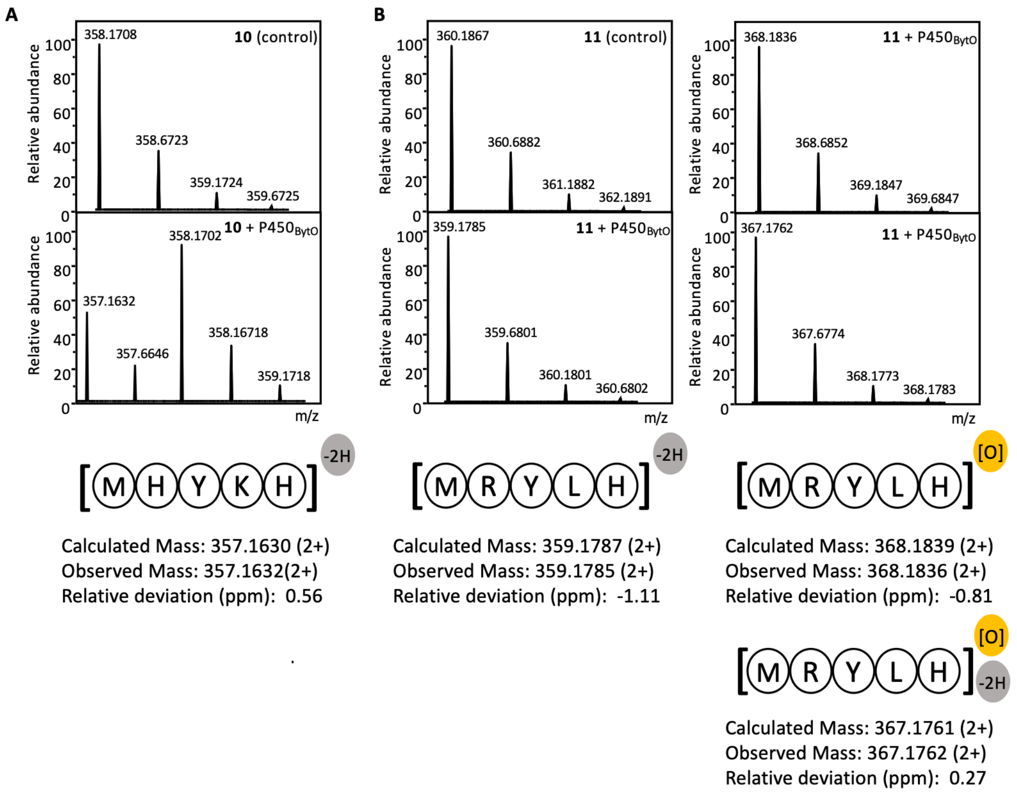

Figure S15. Characterization of **10** and **11**. **A** and **B)** HPLC-ESI-MS characterization of **10** and **11** (control) and after incubation with $P450_{BytO}$ are shown. Observed and calculated mass values are shown and the modifications installed by $P450_{BytO}$ are highlighted.

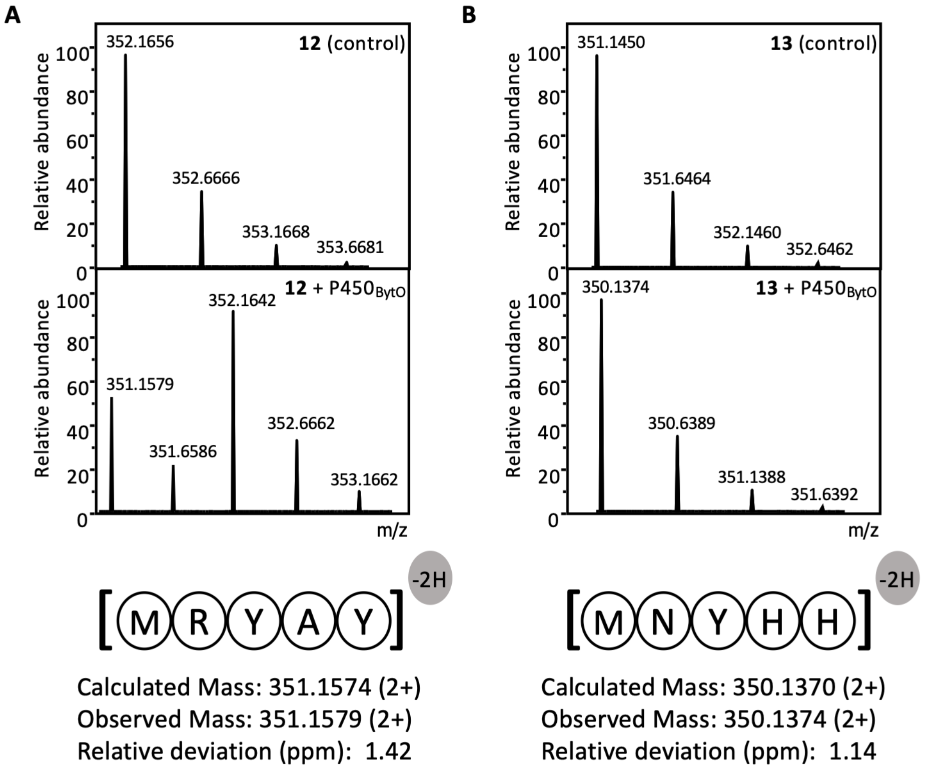

Figure S16. Characterization of **12** and **13**. HPLC-ESI-MS characterization of **12** and **13** (control) and after incubation with $P450_{BytO}$ are shown. Observed and calculated mass values are shown and the modification installed by $P450_{BytO}$ are highlighted.

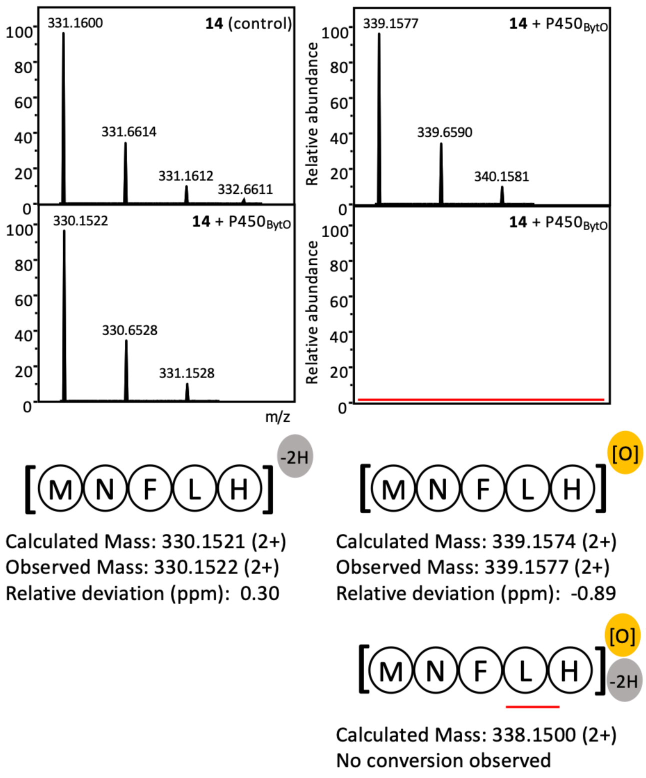

Figure S17. Characterization of **14**. **A** and **B)** HPLC-ESI-MS characterization of **14** (control) and after incubation with P450$_{\text{BytO}}$ are shown. Observed and calculated mass values are shown and the modifications installed by P450$_{\text{BytO}}$ are highlighted.

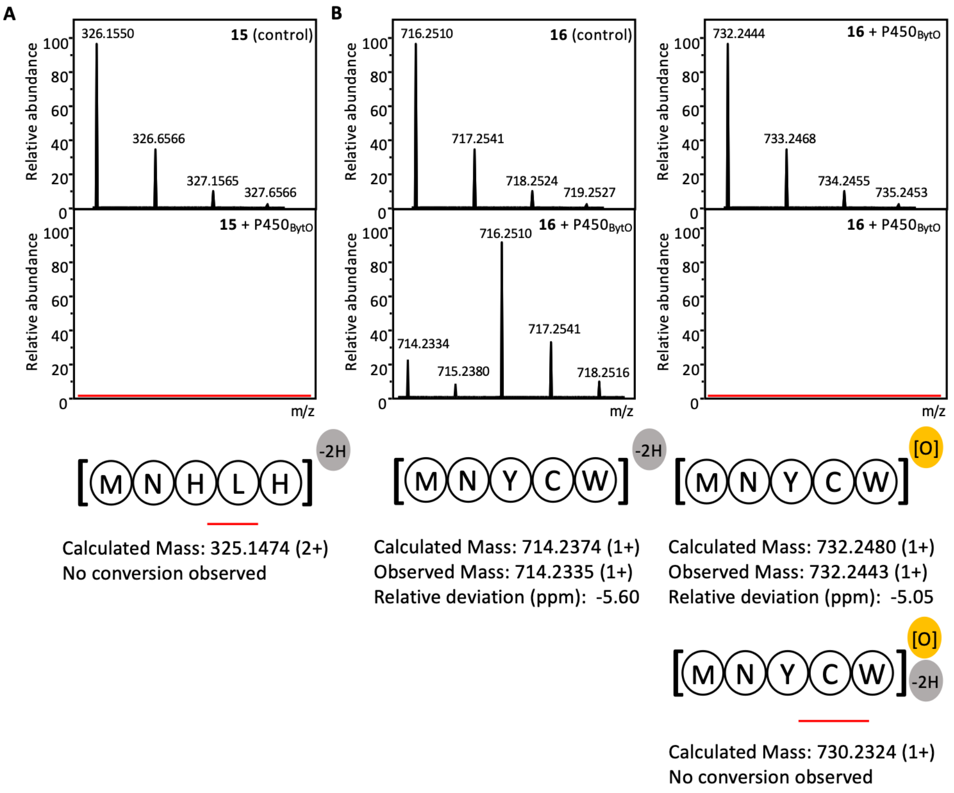

Figure S18. Characterization of **15** and **16**. **A** and **B)** HPLC-ESI-MS characterization of **15** and **16** (control) and after incubation with P450$_{\text{BytO}}$ are shown. Observed and calculated mass values are shown and the modifications installed by P450$_{\text{BytO}}$ are highlighted.

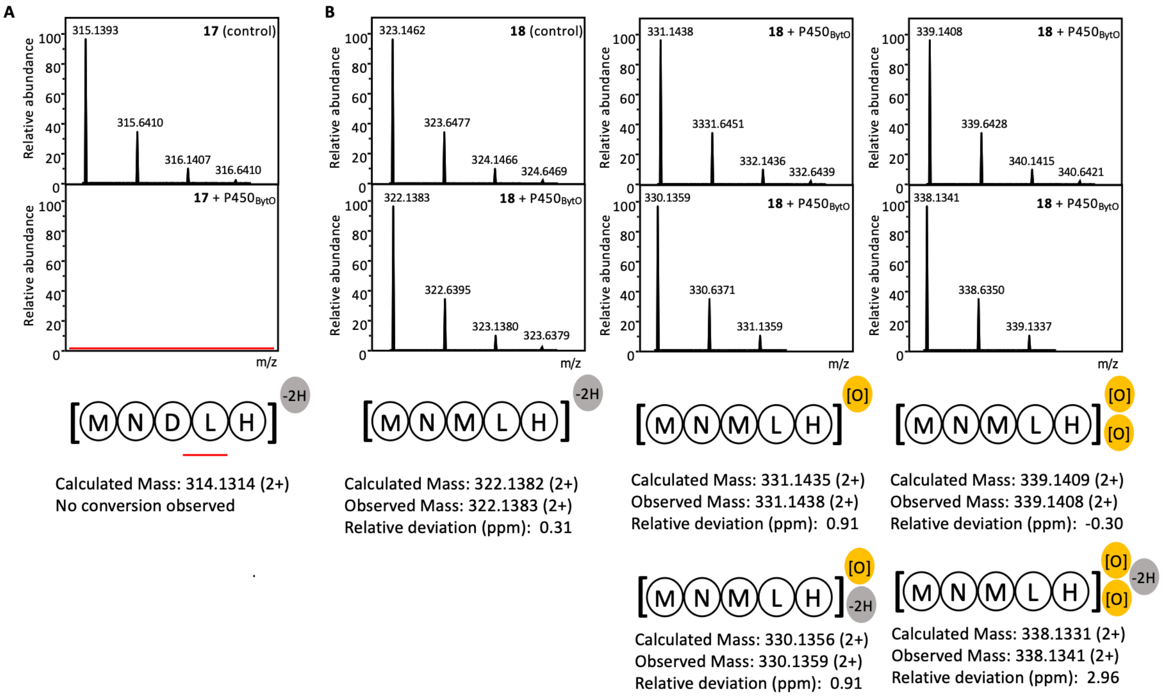

Figure S19. Characterization of **17** and **18**. **A** and **B)** HPLC-ESI-MS characterization of **17** and **18** (control) and after incubation with $P450_{BytO}$ are shown. Observed and calculated mass values are shown and the modifications installed by $P450_{BytO}$ are highlighted.

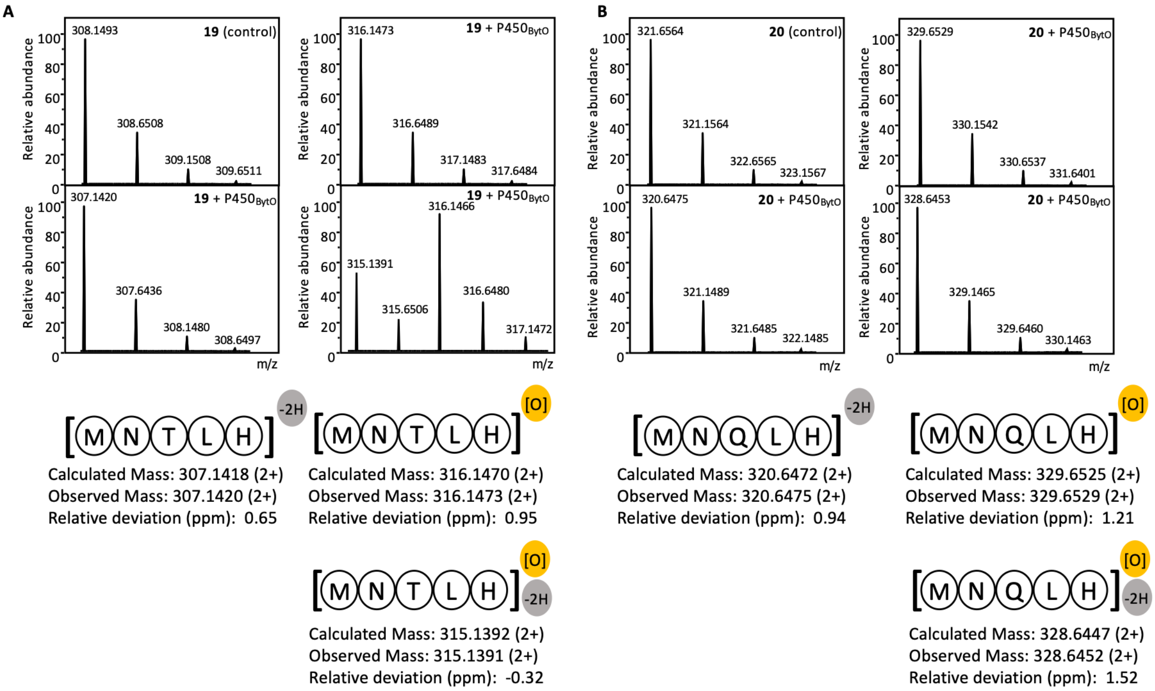

Figure S20. Characterization of **19** and **20**. **A** and **B)** HPLC-ESI-MS characterization of **19** and **20** (control) and after incubation with $P450_{BytO}$ are shown. Observed and calculated mass values are shown and the modifications installed by $P450_{BytO}$ are highlighted.

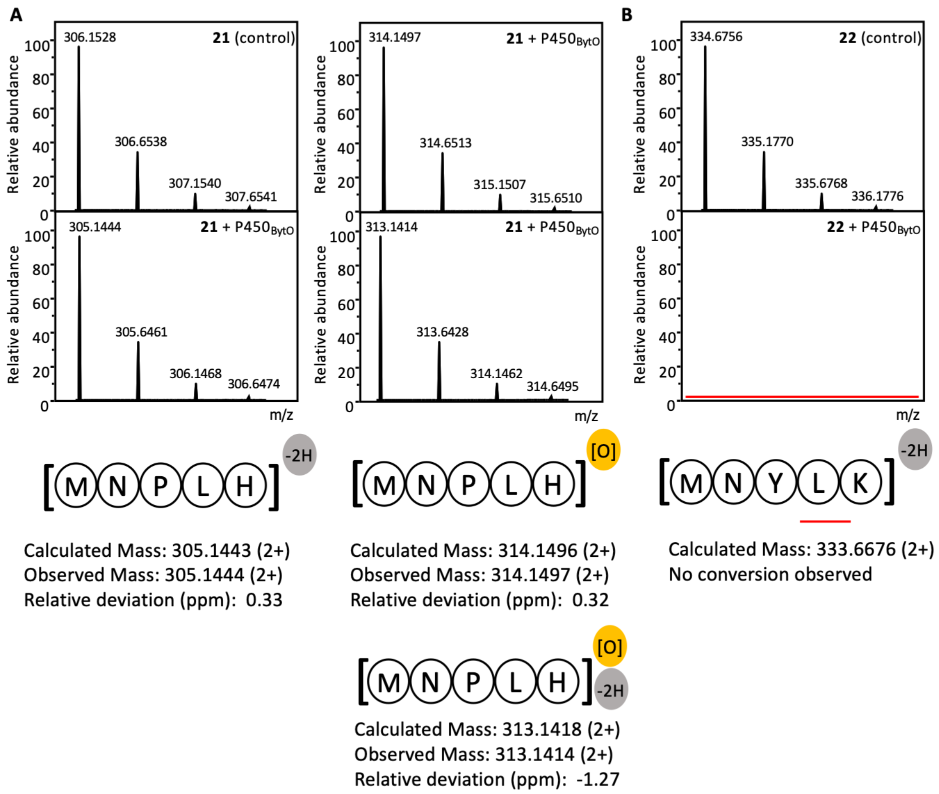

Figure S21. Characterization of **21** and **22**. **A** and **B)** HPLC-ESI-MS characterization of **21** and **22** (control) and after incubation with P450$_{\text{BytO}}$ are shown. Observed and calculated mass values are shown and the modifications installed by P450$_{\text{BytO}}$ are highlighted.

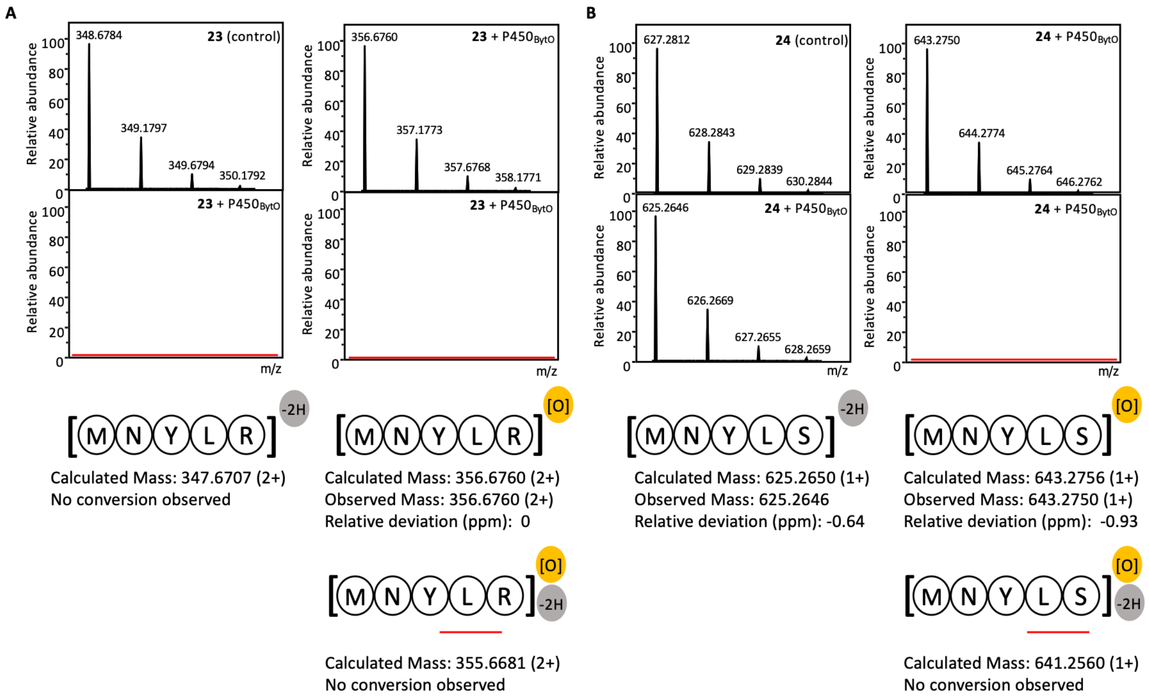

Figure S22. Characterization of **23** and **24**. **A** and **B)** HPLC-ESI-MS characterization of **23** and **24** (control) and after incubation with P450$_{\text{BytO}}$ are shown. Observed and calculated mass values are shown and the modifications installed by P450$_{\text{BytO}}$ are highlighted.

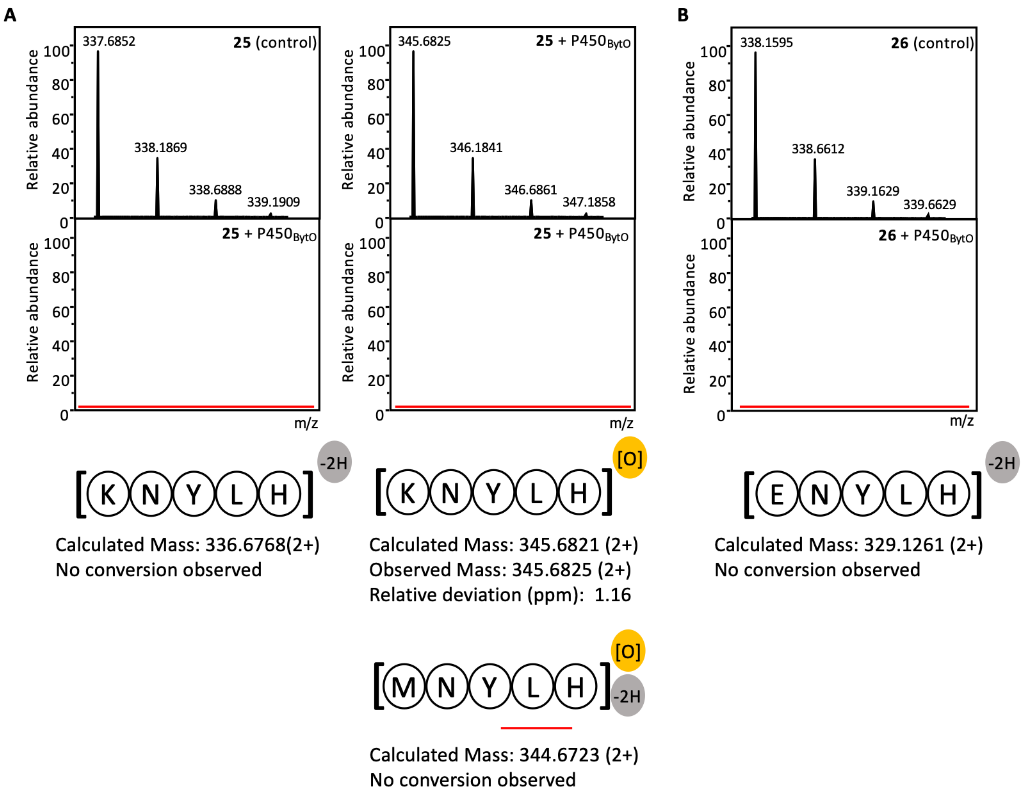

Figure S23. Characterization of **25** and **26**. **A** and **B)** HPLC-ESI-MS characterization of **25** and **26** (control) and after incubation with $P450_{BytO}$ are shown. Observed and calculated mass values are shown and the modifications installed by $P450_{BytO}$ are highlighted.

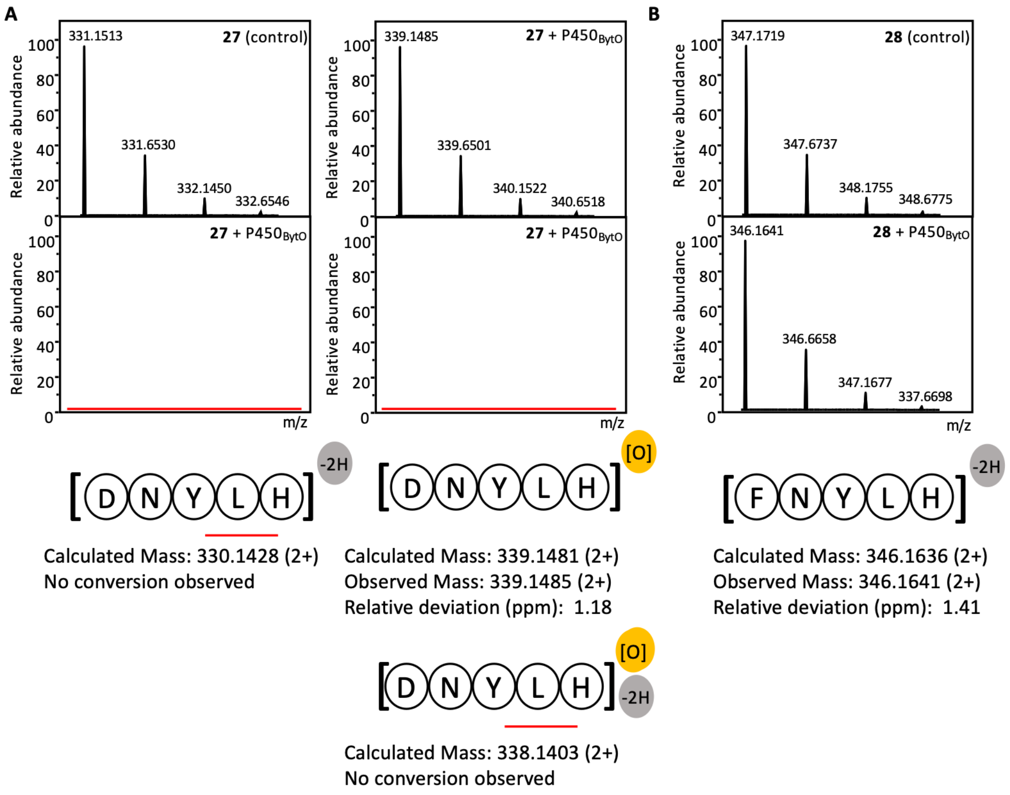

Figure S24. Characterization of **27** and **28**. **A** and **B)** HPLC-ESI-MS characterization of **27** and **28** (control) and after incubation with $P450_{BytO}$ are shown. Observed and calculated mass values are shown and the modifications installed by $P450_{BytO}$ are highlighted.

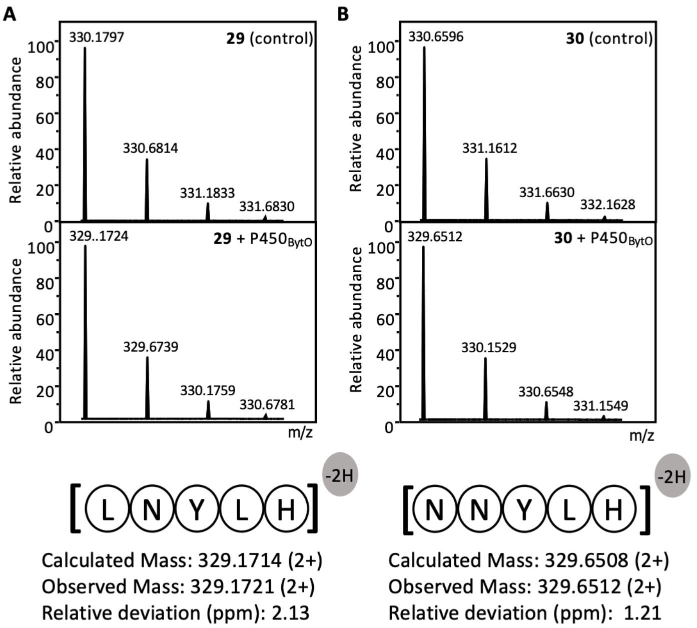

Figure S25. Characterization of **29** and **30**. **A** and **B)** HPLC-ESI-MS characterization of **30** and **31** (control) and after incubation with $P450_{BytO}$ are shown. Observed and calculated mass values are shown and the modification installed by $P450_{BytO}$ are highlighted.

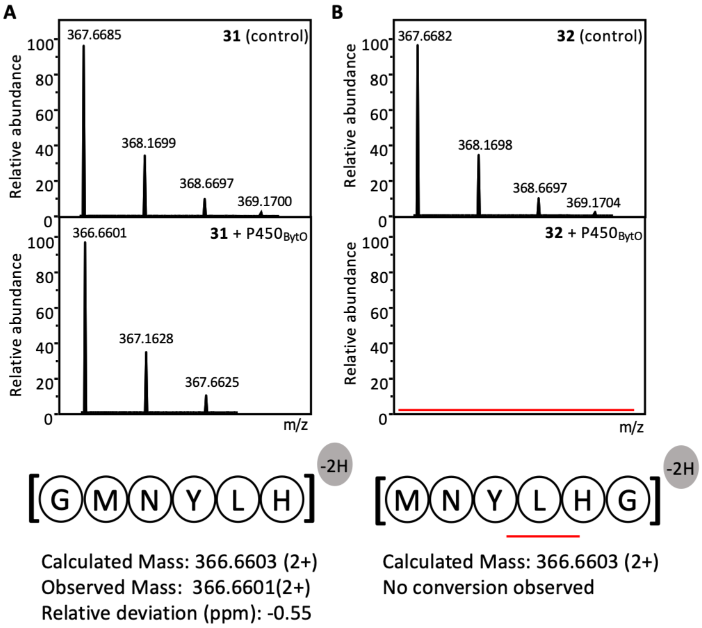

Figure S26. Characterization of **31** and **32**. **A** and **B)** HPLC-ESI-MS characterization of **31** and **32** (control) and after incubation with $P450_{BytO}$ are shown. Observed and calculated mass values are shown and the modification installed by $P450_{BytO}$ are highlighted.

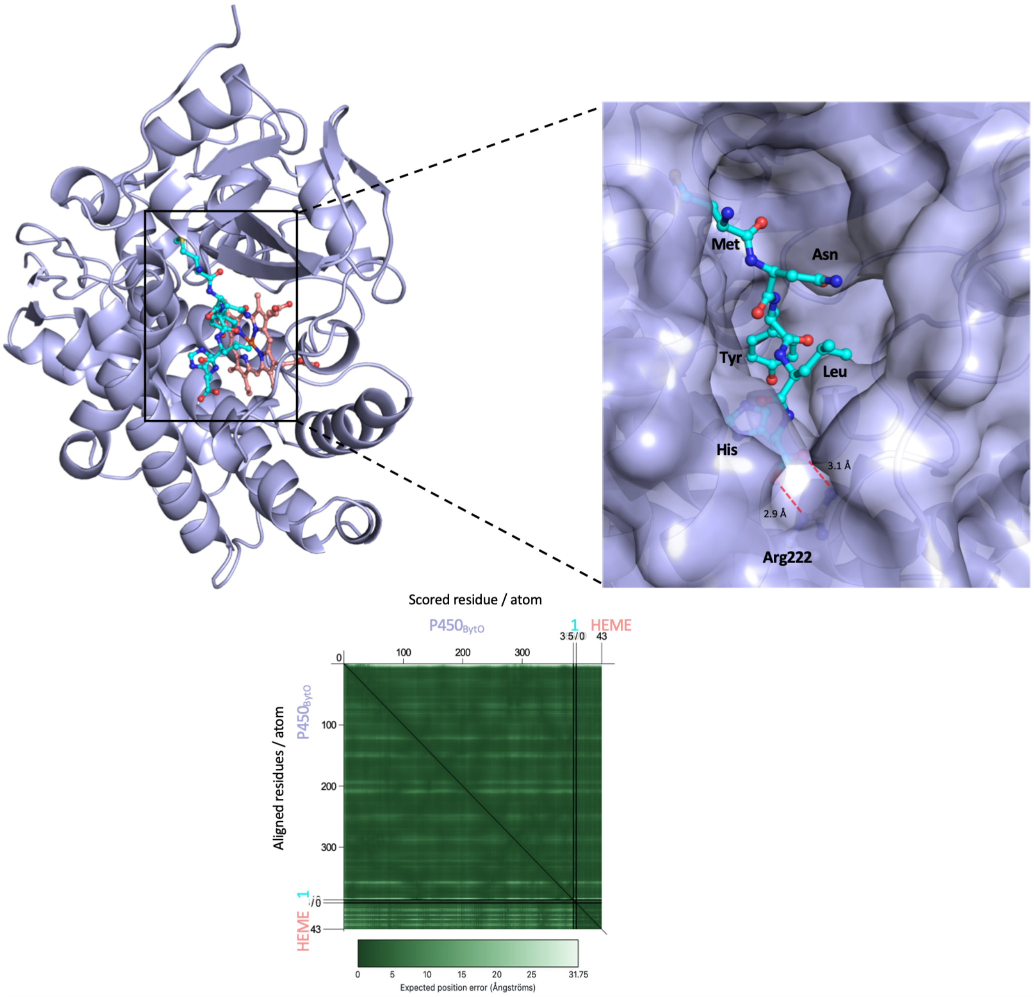

Figure S27. AlphaFold 3 model of P450$_{\text{BytO}}$–**1**–Heme complex. Cartoon representation of the P450$_{\text{BytO}}$ –**1**–Heme complex (P450$_{\text{BytO}}$, light blue; **1**, cyan; and heme group, orange). A close-up view of the substrate-binding pocket highlights the predicted binding pose of **1** (peptide sequence: MNYLH; residues labeled). The bidentate interaction between Arg222 and the terminal carbonyl group of **1** that likely help anchor **1** in the pocket is shown as dotted red lines with distances given in Å. The bottom panel displays the predicted aligned error (PAE) heatmap along with the error score generated via PAE viewer.[21] The predicted template modelling (pTM) and the predicted interface modelling (ipTM) scores were 0.96 and 0.94, respectively, indicating very high confidence in the model.

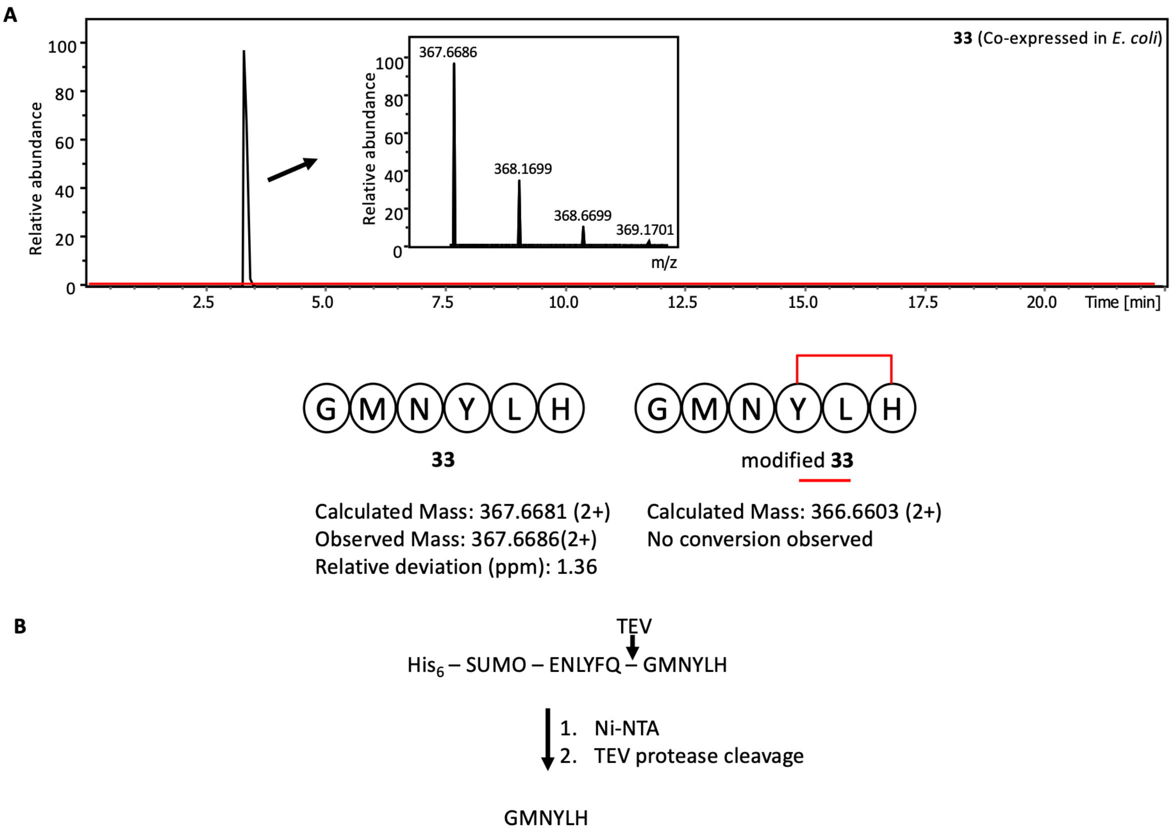

Figure S28. Characterization of co-expressed **33**. **A**) HPLC-ESI-MS analyses of **33** and **33** co-expressed with P450$_{\mathrm{BytO}}$ and FdR/YkuN in *E. coli* are shown. The observed and the calculated masses for the heterologous expressed and TEV- cleaved **33** are shown at the bottom. **B**) The co-expressed **33** was isolated using Ni-NTA, desalted and treated with TEV protease overnight before being analysed LC-HRMS.

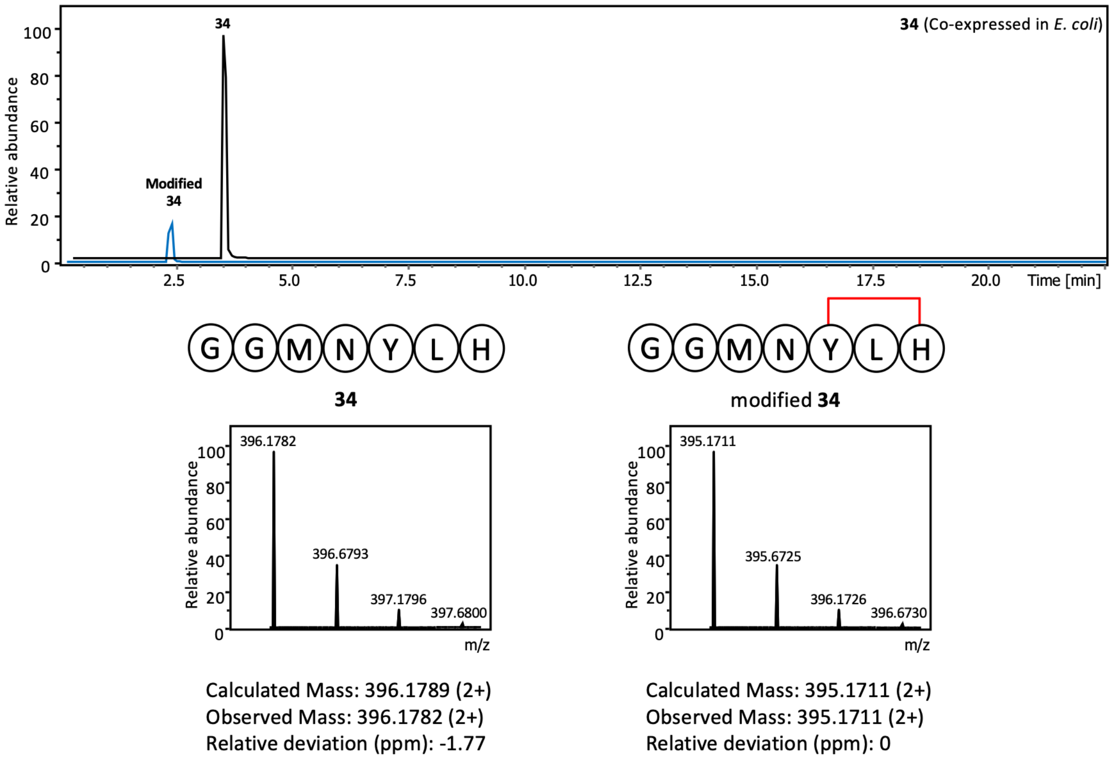

Figure S29. Characterization of co-expressed **34**. EICs of modified and unmodified **34** when co-expressed with P450$_{\mathrm{BytO}}$ and FdR/YkuN in *E. coli* are shown. Major peak corresponds to the unmodified **34**. The observed and the calculated masses for the heterologously expressed and TEV- cleaved **34** are shown at the bottom. HR-MS/MS characterization can be found in Figure S30.

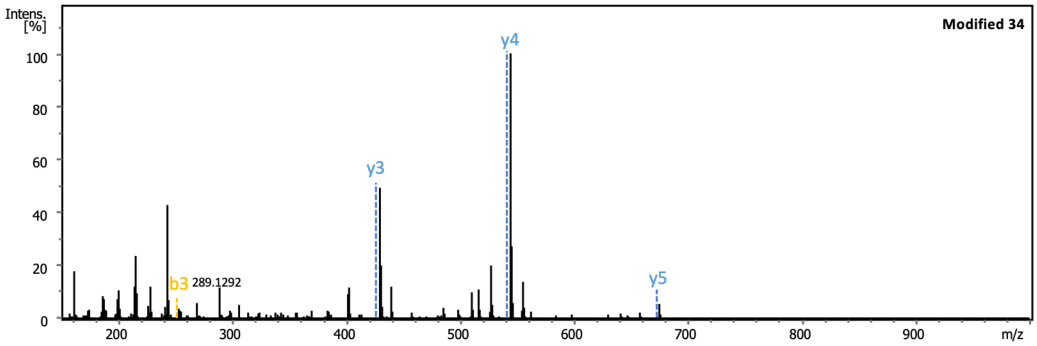

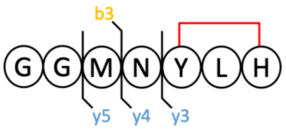

	Observed Mass m/z	Expected Mass m/z	Relative deviation (ppm)
b3	246.0902	246.0912	-4.06
y3	430.2083	430.2079	0.93
y4	544.2511	544.2509	0.37
y5	675.2920	675.2914	0.89

Figure S30. Characterization of modified **34**. HR-MS/MS analysis of modified **34** is shown. *b* and *y* ions are listed in the table and marked in the spectrum.

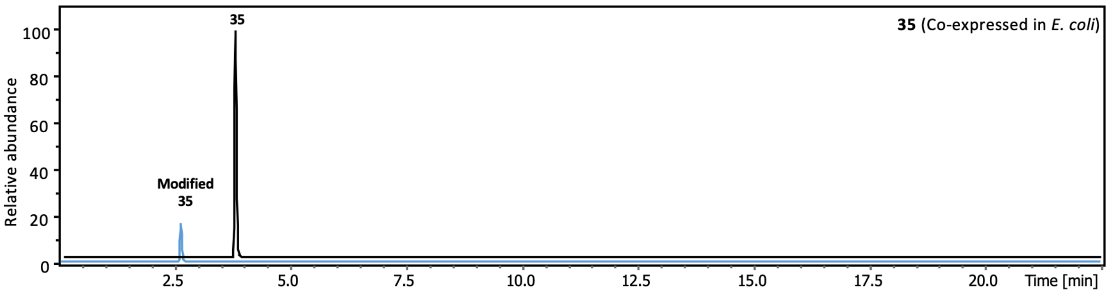

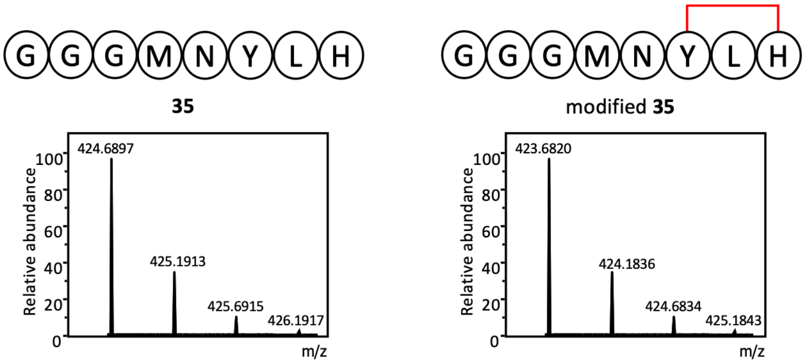

Calculated Mass: 424.6896 (2+)
Observed Mass: 424.6897 (2+)
Relative deviation (ppm): 0.24

Calculated Mass: 423.6818 (2+)
Observed Mass: 423.6820 (2+)
Relative deviation (ppm): 0.47

Figure S31. Characterization of co-expressed **35**. EICs of modified and unmodified **35** when co-expressed with $P450_{BytO}$ and FdR/YkuN in *E. coli* are shown. Major peak corresponds to the unmodified **35**. The observed and the calculated masses for the heterologously expressed and TEV- cleaved **35** are shown at the bottom. HR-MS/MS characterization can be found in Figure S32.

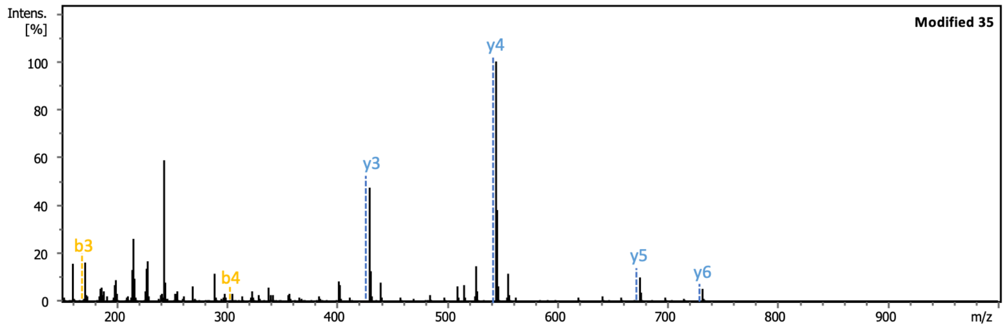

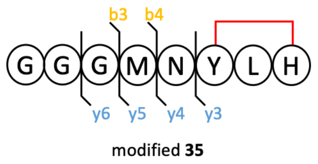

	Observed Mass m/z	Expected Mass m/z	Relative deviation (ppm)
b3	172.0730	172.0722	4.65
b4	303.1131	303.1127	1.32
y3	430.2100	430.2079	4.88
y4	544.2540	544.2509	5.70
y5	675.2950	675.2914	5.33
y6	732.3178	732.3128	5.73

Figure S32. Characterization of modified **35**. HR-MS/MS analysis of modified **35** is shown. *b* and *y* ions are listed in the table and marked in the spectrum.

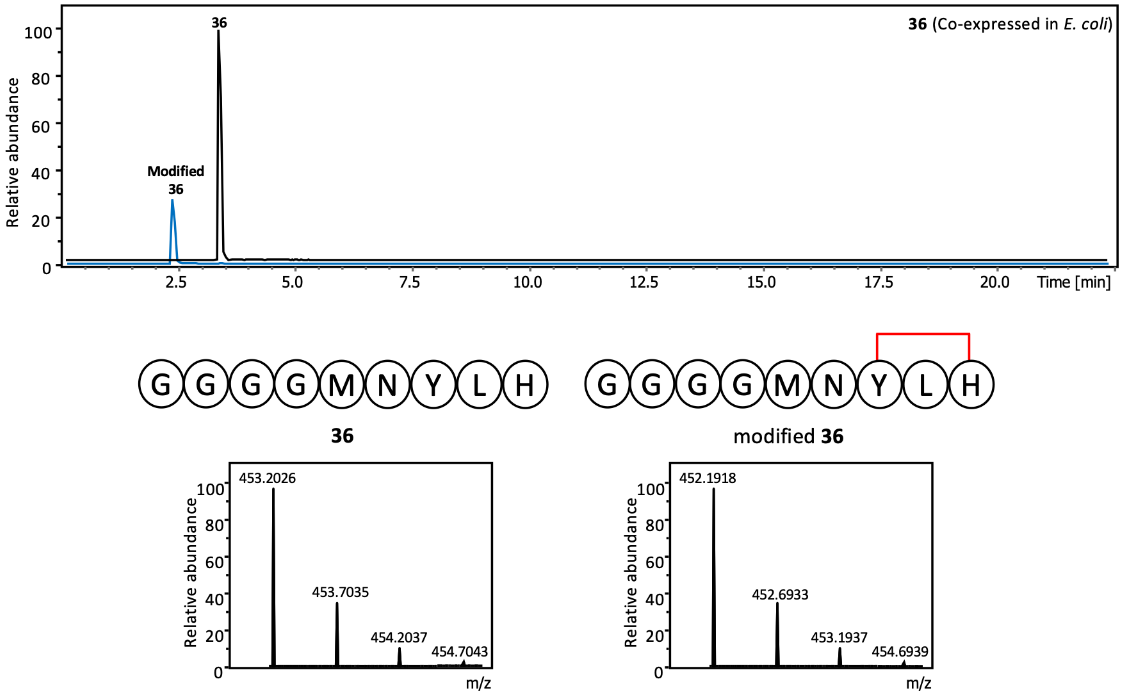

Calculated Mass: 453.2003 (2+)
Observed Mass: 453.2026(2+)
Relative deviation (ppm): 5.08

Calculated Mass: 452.1925 (2+)
Observed Mass: 452.1918 (2+)
Relative deviation (ppm): -1.55

Figure S33. Characterization of co-expressed **36**. EICs of modified and unmodified **36** when co-expressed with $P450_{BytO}$ and FdR/YkuN in *E. coli* are shown. Major peak corresponds to the unmodified **36**. The observed and the calculated masses for the heterologously expressed and TEV- cleaved **36** are shown at the bottom. HR-MS/MS characterization can be found in Figure S34.

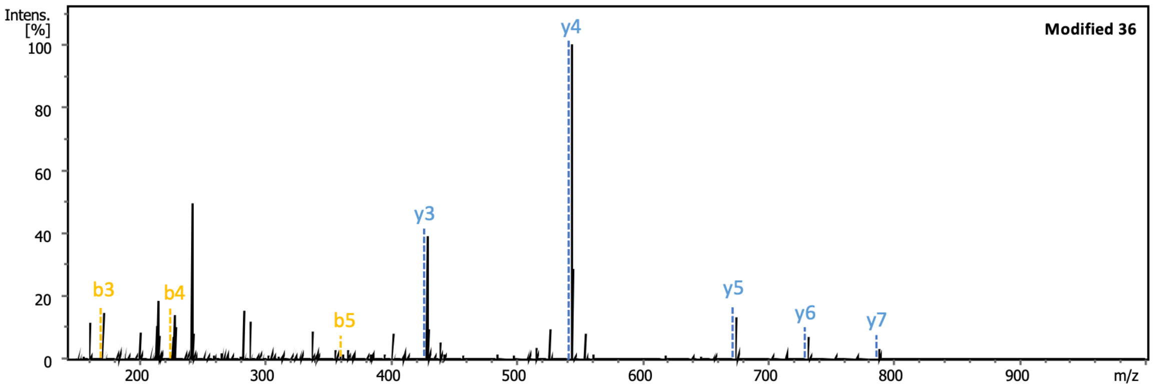

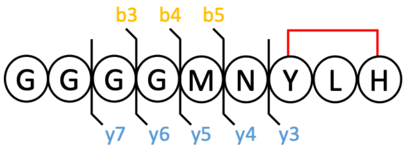

Modified 36

	Observed Mass m/z	Expected Mass m/z	Relative deviation (ppm)
b3	172.0722	172.0714	4.65
b4	229.0937	229.0927	4.37
b5	360.1342	360.1326	4.44
y3	430.2079	430.2075	0.93
y4	544.2509	544.2501	1.47
y5	675.2914	675.2900	2.07
y6	732.3128	732.3118	1.37
y7	789.3328	789.3343	-1.90

Figure S34. Characterization of modified **36**. HR-MS/MS analysis of modified **36** is shown. *b* and *y* ions are listed in the table and marked in the spectrum.

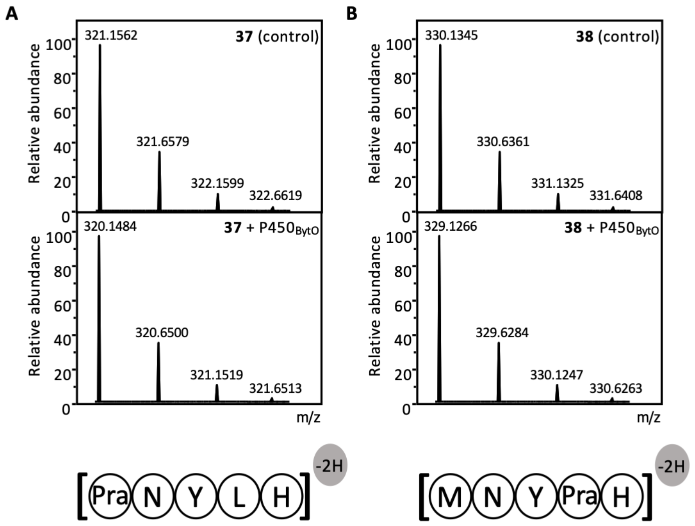

Calculated Mass: 320.1479 (2+)
Observed Mass: 320.1484 (2+)
Relative deviation (ppm): 1.56

Calculated Mass: 329.1261 (2+)
Observed Mass: 329.1266 (2+)
Relative deviation (ppm): 1.52

Figure S35. Characterization of **37** and **38**. **A** and **B)** HPLC-ESI-MS characterization of propargylglycine (Pra) containing **37** (control) and **38** (control) and after incubation with $P450_{BytO}$ are shown. Observed and calculated mass values are shown and the modification installed by $P450_{BytO}$ are highlighted.

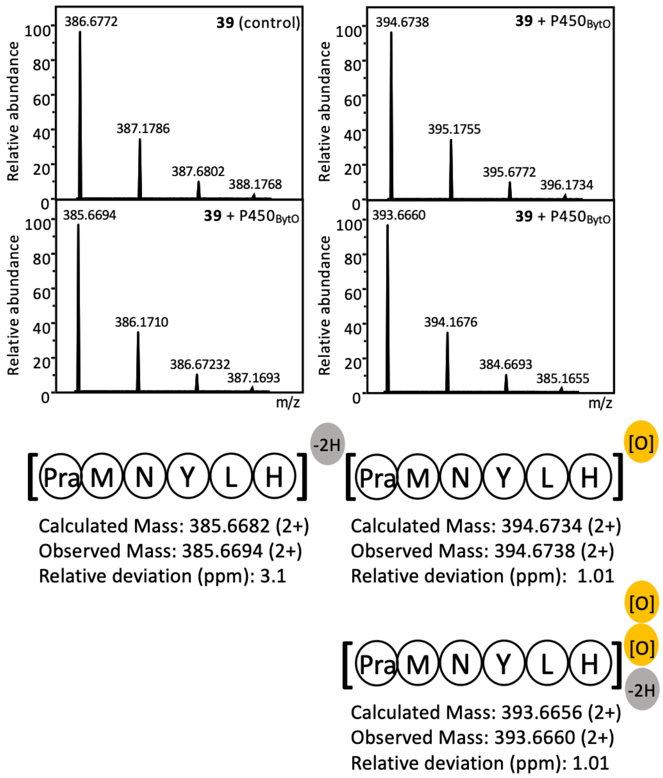

Figure S36. Characterization of **39**. **A** and **B)** HPLC-ESI-MS characterization of propargylglycine (Pra) containing **39** (control) and after incubation with $P450_{BytO}$ are shown. Observed and calculated mass values are shown and the modifications installed by $P450_{BytO}$ are highlighted.

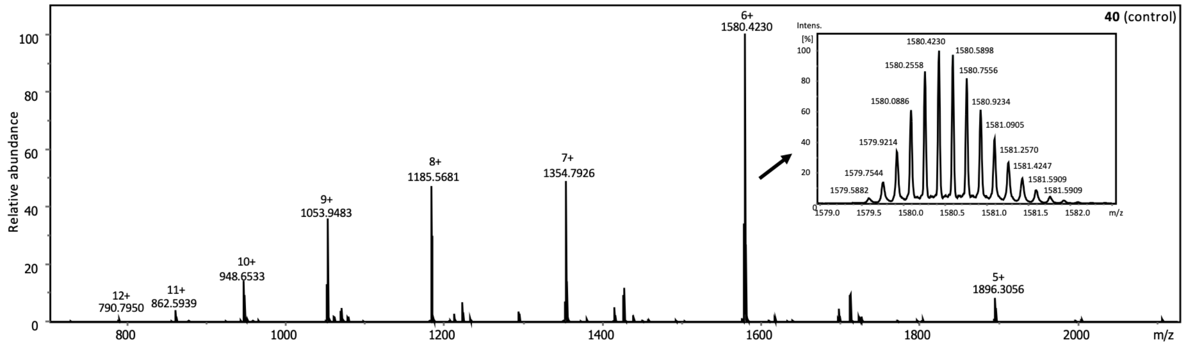

Hybrid precursor peptide (**40**): GAMVYSEAMVSAVDETAFADLVSKIQDAEASMTDEQRAVIDPASGEKALQELAGVSPEELQAFLEEKAGISPDEEAQGSVMAAAGGMNYLH

Calculated monoisotopic mass: 1579.5834 (6+)

Observed Mass: 1579.5888 (6+)

Relative deviation (ppm): 3.42

Figure S37. Characterization of **40**. HPLC-ESI-MS analysis of **40** (control) used for *in vitro* studies is shown. The modified thioholgamide core peptide[1] is underlined with sequences of the linker (GG, blue) and **1** (MNYLH, red) highlighted.

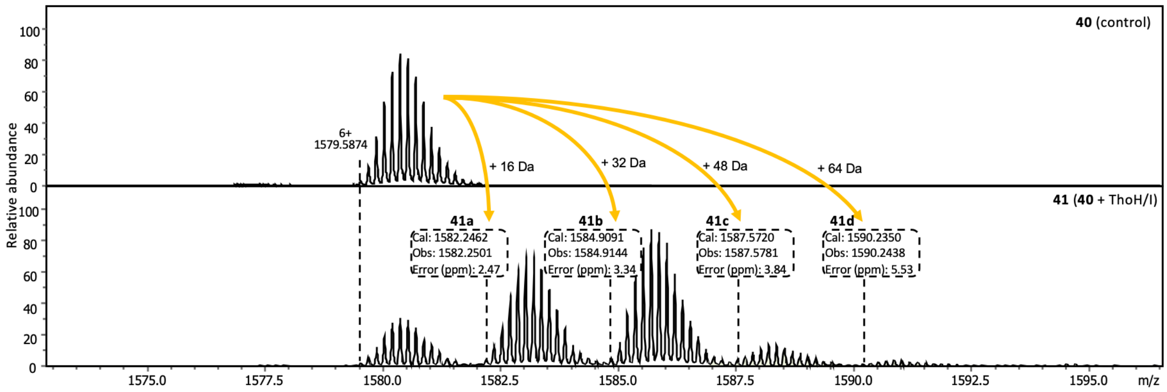

Figure S38. Characterization of **41**. HPLC-ESI-MS analysis of **40** (control, top) and after reaction with ThoH/I in the presence of ATP and sodium sulfide (bottom). Only the $[M+6H]^{6+}$ charge state is shown. HR-MS/MS analyses of **41a** and **41b** can be found in Figures S39 and S40.

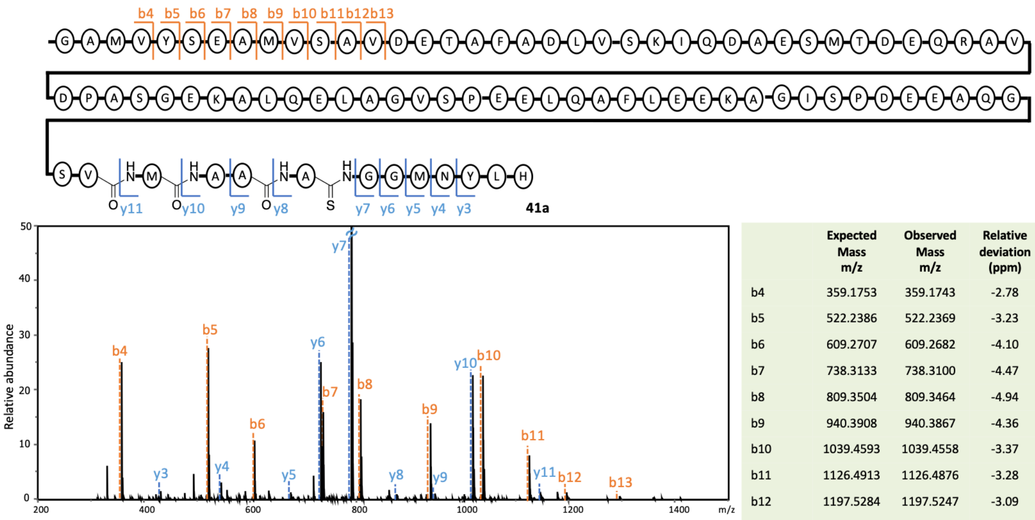

	Expected Mass m/z	Observed Mass m/z	Relative deviation (ppm)
b4	359.1753	359.1743	-2.78
b5	522.2386	522.2369	-3.23
b6	609.2707	609.2682	-4.10
b7	738.3133	738.3100	-4.47
b8	809.3504	809.3464	-4.94
b9	940.3908	940.3867	-4.36
b10	1039.4593	1039.4558	-3.37
b11	1126.4913	1126.4876	-3.28
b12	1197.5284	1197.5247	-3.09
b13	1296.5968	1296.5974	0.46
y3	432.2236	432.2234	-0.46
y4	546.2665	546.2659	-1.10
y5	677.3070	677.3059	-1.62
y6	734.3285	734.3265	-2.72
y7	791.3499	791.3488	-1.39
y8	878.3642	878.3620	-2.50
y9	949.4013	949.3987	-2.74
y10	1020.4384	1020.4365	-1.86
y11	1151.4789	1151.4781	-0.69

Figure S39. Characterization of **41a**. HR-MS/MS analysis of **41a** is shown and the *b* and *y* ions are listed in the table and marked in the spectrum.

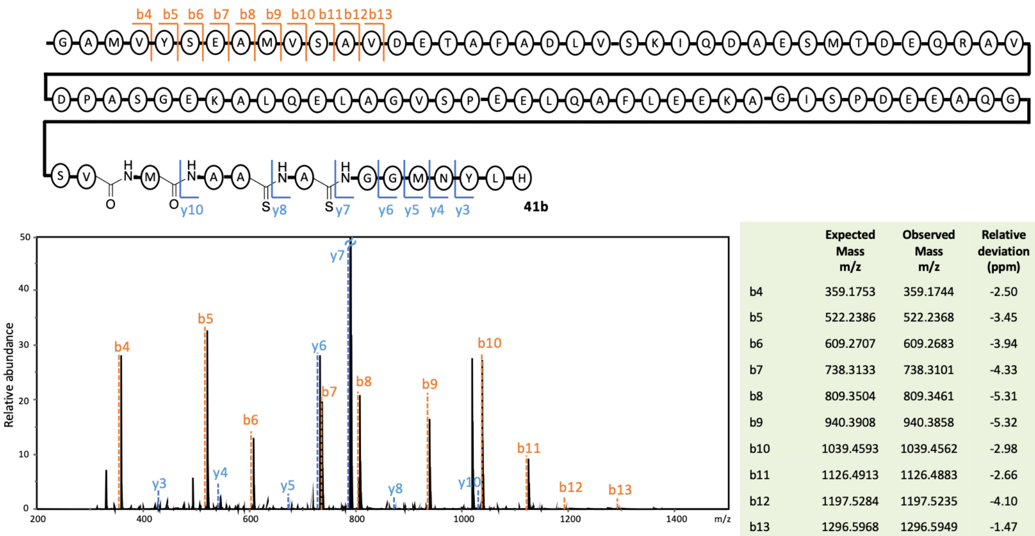

	Expected Mass m/z	Observed Mass m/z	Relative deviation (ppm)
b4	359.1753	359.1744	-2.50
b5	522.2386	522.2368	-3.45
b6	609.2707	609.2683	-3.94
b7	738.3133	738.3101	-4.33
b8	809.3504	809.3461	-5.31
b9	940.3908	940.3858	-5.32
b10	1039.4593	1039.4562	-2.98
b11	1126.4913	1126.4883	-2.66
b12	1197.5284	1197.5235	-4.10
b13	1296.5968	1296.5949	-1.47
y3	432.2236	432.2223	-3.01
y4	546.2665	546.2646	-3.48
y5	677.3070	677.3050	-2.95
y6	734.3285	734.3266	-2.59
y7	791.3499	791.3490	-1.14
y8	878.3642	878.3614	-3.20
y10	1036.4156	1036.4172	1.54

Figure S40. Characterization of **41b**. HR-MS/MS analysis of **41b** is shown and the *b* and *y* ions are listed in the table and marked in the spectrum.

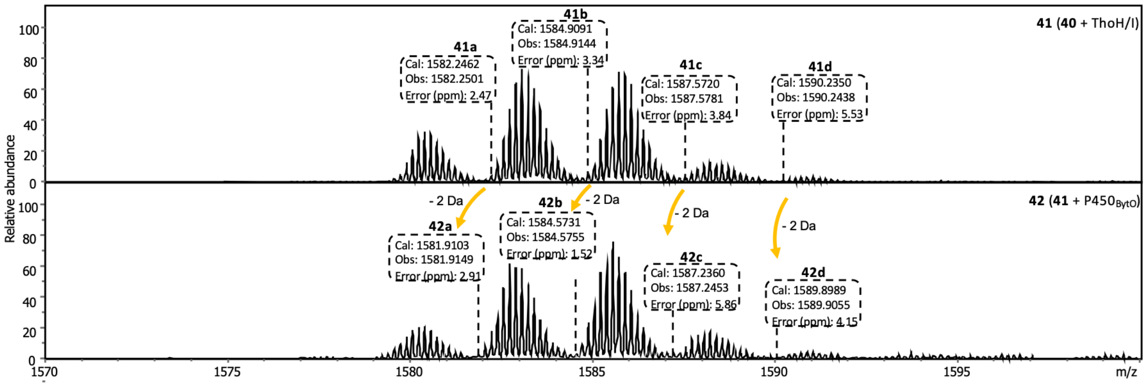

Figure S41. Characterization of **42**. HPLC-ESI-MS analysis of **41** (control, top) and after reaction with $P450_{BytO}$ in the presence FdR/YkuN. Only the $[M+6H]^{6+}$ charge state is shown. HR-MS/MS analyses of **42a** and **42b** can be found in Figure S42 and S43.

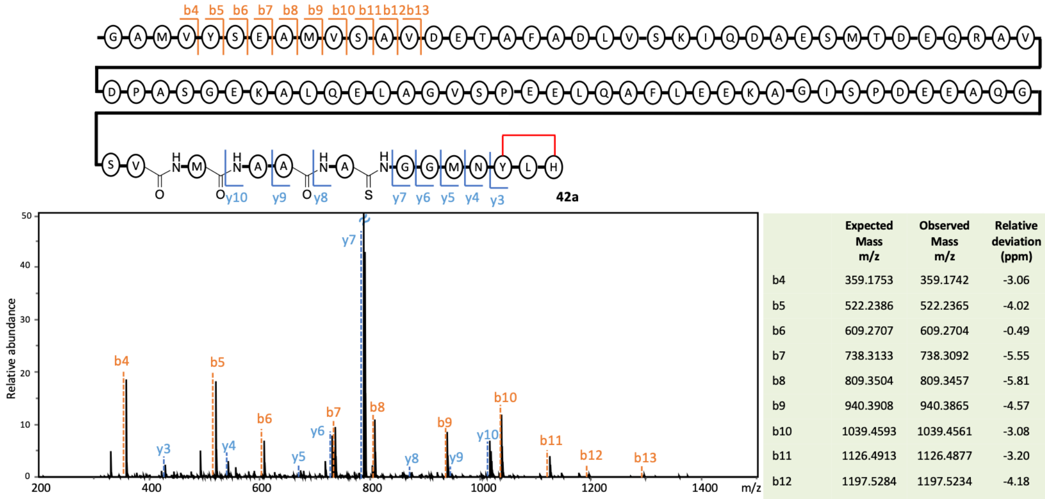

	Expected Mass m/z	Observed Mass m/z	Relative deviation (ppm)
b4	359.1753	359.1742	-3.06
b5	522.2386	522.2365	-4.02
b6	609.2707	609.2704	-0.49
b7	738.3133	738.3092	-5.55
b8	809.3504	809.3457	-5.81
b9	940.3908	940.3865	-4.57
b10	1039.4593	1039.4561	-3.08
b11	1126.4913	1126.4877	-3.20
b12	1197.5284	1197.5234	-4.18
b13	1296.5968	1296.5986	1.39
y3	430.2079	430.2075	-0.93
y4	544.2509	544.2495	-2.57
y5	675.2914	675.2898	-2.37
y6	732.3128	732.3122	-0.82
y7	789.3343	789.3319	-3.04
y8	876.3486	876.3449	-4.22
y9	947.3857	947.3797	-6.33
y10	1018.4218	1018.4217	-0.10

Figure S42. Characterization of **42a**. HR-MS/MS analysis of **42a** is shown and the *b* and *y* ions are listed in the table and marked in the spectrum. Biaryl-linkage is shown as red square bracket.

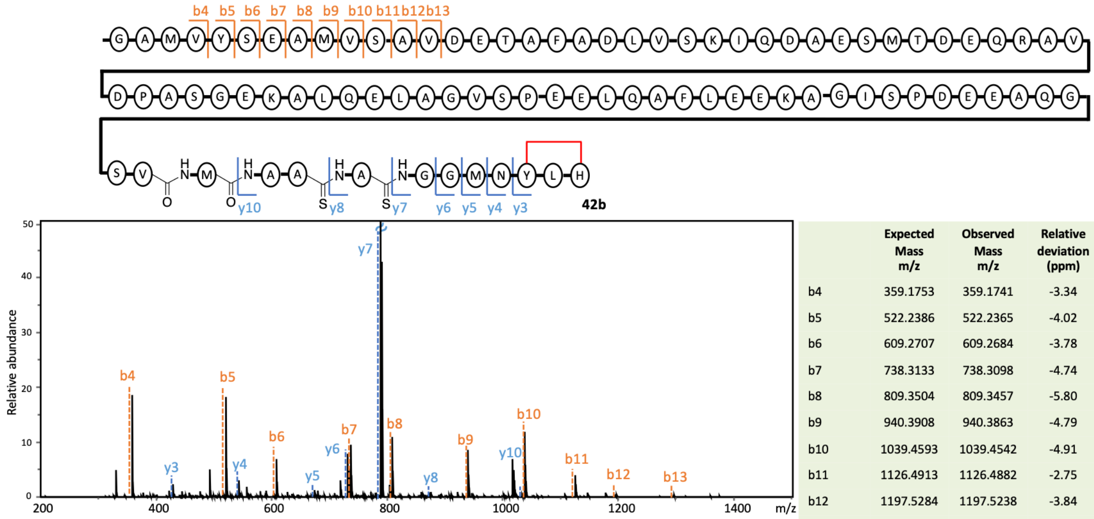

	Expected Mass m/z	Observed Mass m/z	Relative deviation (ppm)
b4	359.1753	359.1741	-3.34
b5	522.2386	522.2365	-4.02
b6	609.2707	609.2684	-3.78
b7	738.3133	738.3098	-4.74
b8	809.3504	809.3457	-5.80
b9	940.3908	940.3863	-4.79
b10	1039.4593	1039.4542	-4.91
b11	1126.4913	1126.4882	-2.75
b12	1197.5284	1197.5238	-3.84
b13	1296.5968	1296.5993	1.93
y3	430.2079	430.2077	-0.46
y4	544.2509	544.2494	-2.76
y5	675.2914	675.2884	-4.44
y6	732.3128	732.3096	-4.37
y7	789.3343	789.3319	-3.04
y8	876.3486	876.3455	-3.54
y10	1034.3999	1034.4043	4.25

Figure S43. Characterization of **42b**. HR-MS/MS analysis of **42b** is shown and the *b* and *y* ions are listed in the table and marked in the spectrum. Biaryl-linkage is shown as red square bracket.

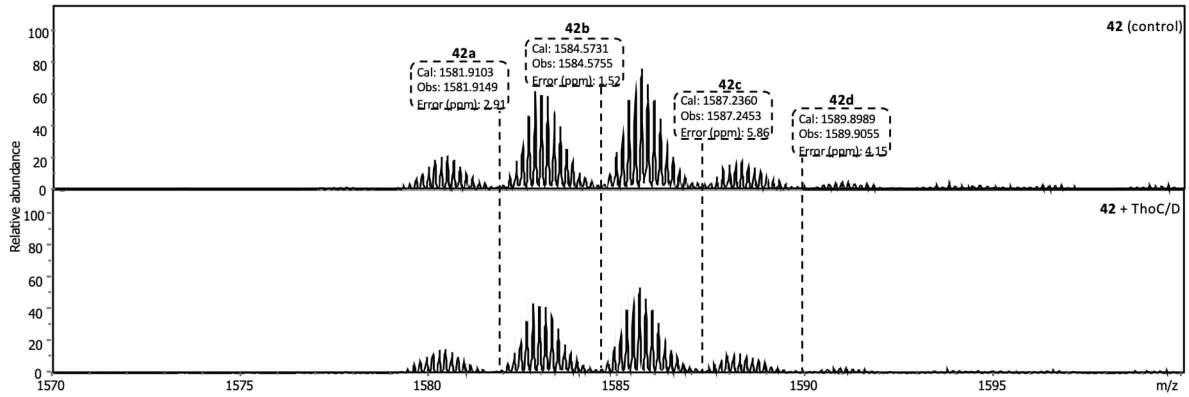

Figure S44. Incubation of **42** with ThoC/D. HPLC-ESI-MS analysis of **42** (control, top) and after reaction with ThoC/D in the presence of excess ATP/Mg^{2+}. We did not observe a specie with a loss of 18 Da, expected for dehydration of Ser1 of the core peptide (peptide sequence given in Figure S37). Only the $[M+6H]^{6+}$ charge state is shown.

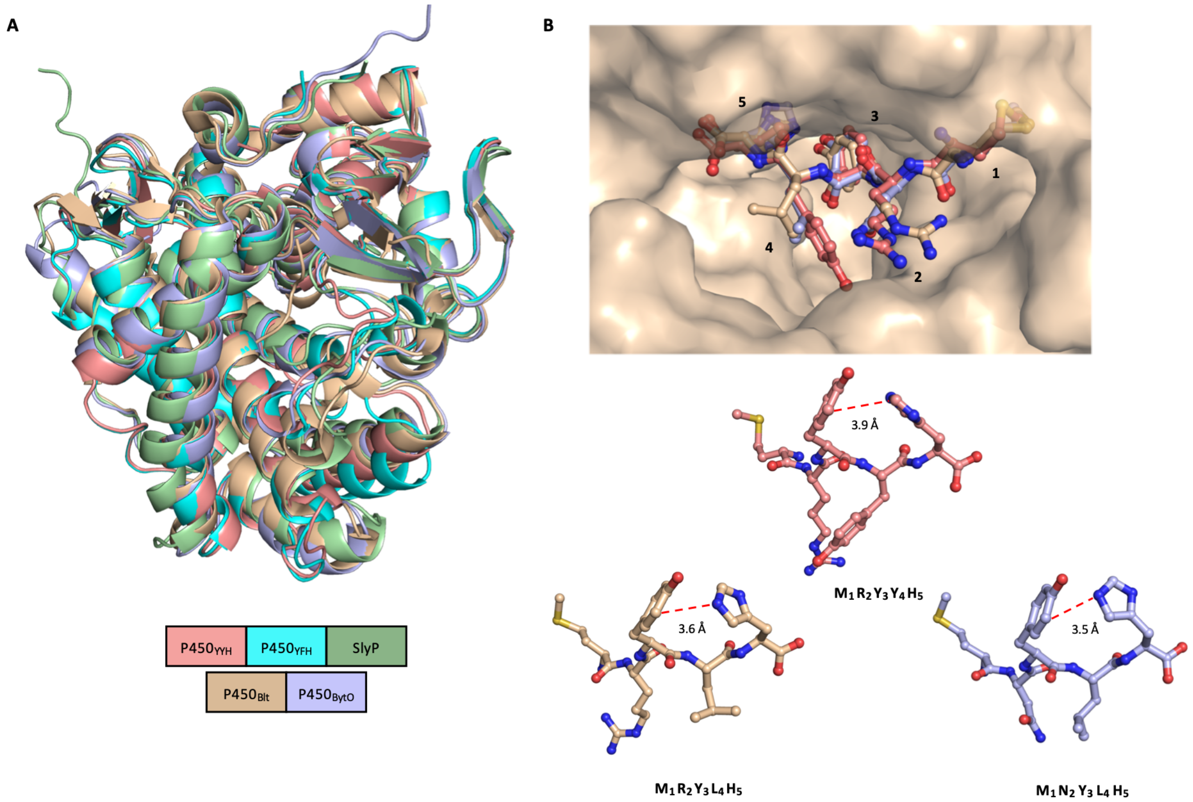

Figure S45. Structural comparison of biarylitide-P450s. **A**) Superposition of AlphaFold 3 predicted structures of cytochrome P450s (YYH, YFH, SlyP and $P450_{BytO}$) with the crystal structure of $P450_{Blt}$ (PDB id: 8u2m).[22] The Cα RMSDs were approx. 1.3 Å (YYH), 1.4 (YFH), 1.3 Å (SlyP) and 1.0 Å ($P450_{BytO}$) over the entire length of the protein. **B**) Comparison of the $P450_{Blt}$ – precursor peptide complex structure (PDB ID: 8u2m) with AlphaFold 3 predicted P450 – precursor peptide complexes (YYH and $P450_{BytO}$). For clarity, only the binding pocket of $P450_{Blt}$ is shown. Precursor peptide residues are numbered, and the distances between the proposed C–C or C–N cross-linking sites are shown. For $P450_{BytO}$ and $P450_{Blt}$ the distance between C–N is shorter than C–C. YxH C–C distance for myxarylin precursor peptide is approx. 3.8 Å. The precursor peptides are color coded according the scheme shown in panel A. YYH and YFH BGCs each encode a cytochrome P450 enzyme, designated BytO, which herein referred to as $P450_{YYH}$ and $P450_{YFH,}$ respectively.

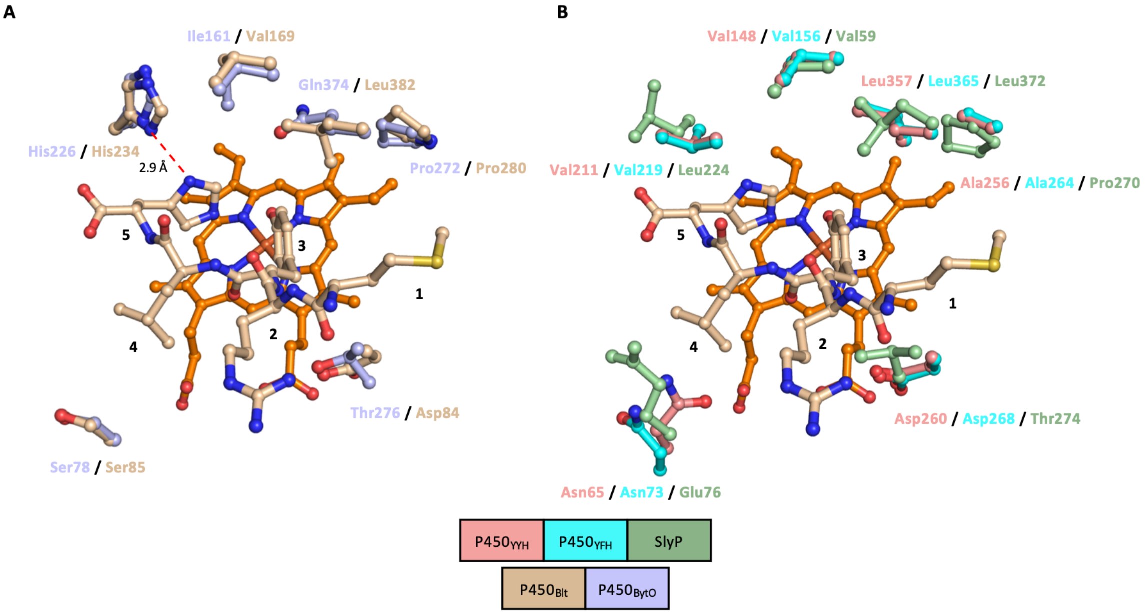

Figure S46. Structural comparison of the substrate-binding pockets of biarylitide-P450s. **A**) Binding pockets of cytochrome P450s involved in C – N biaryl bond formation are shown. **B**) Binding pockets of cytochrome P450s involved in C – C biaryl formation are shown. AlphaFold 3 models of cytochrome P450 (YYH, YFH, SlyP and P450$_{\mathrm{BytO}}$) were superimposed onto the co-crystal structure of P450$_{\mathrm{Blt}}$ (PDB ID: 8u2m) in complex with the precursor peptide (stick, wheat; numbered 1-5) and heme cofactor (stick, orange). The residues are color coded according the scheme shown at the bottom and the distance between His residues is depicted with dotted red line. pLDDT score for all residues was higher than 90 except for Asn65 (YYH; 90 > plDDt > 70) and Asn73 (YFH; 70 > plDDt > 50). YYH and YFH BGCs each encode a cytochrome P450 enzyme, designated BytO, which herein referred to as P450$_{\mathrm{YYH}}$ and P450$_{\mathrm{YFH,}}$ respectively.

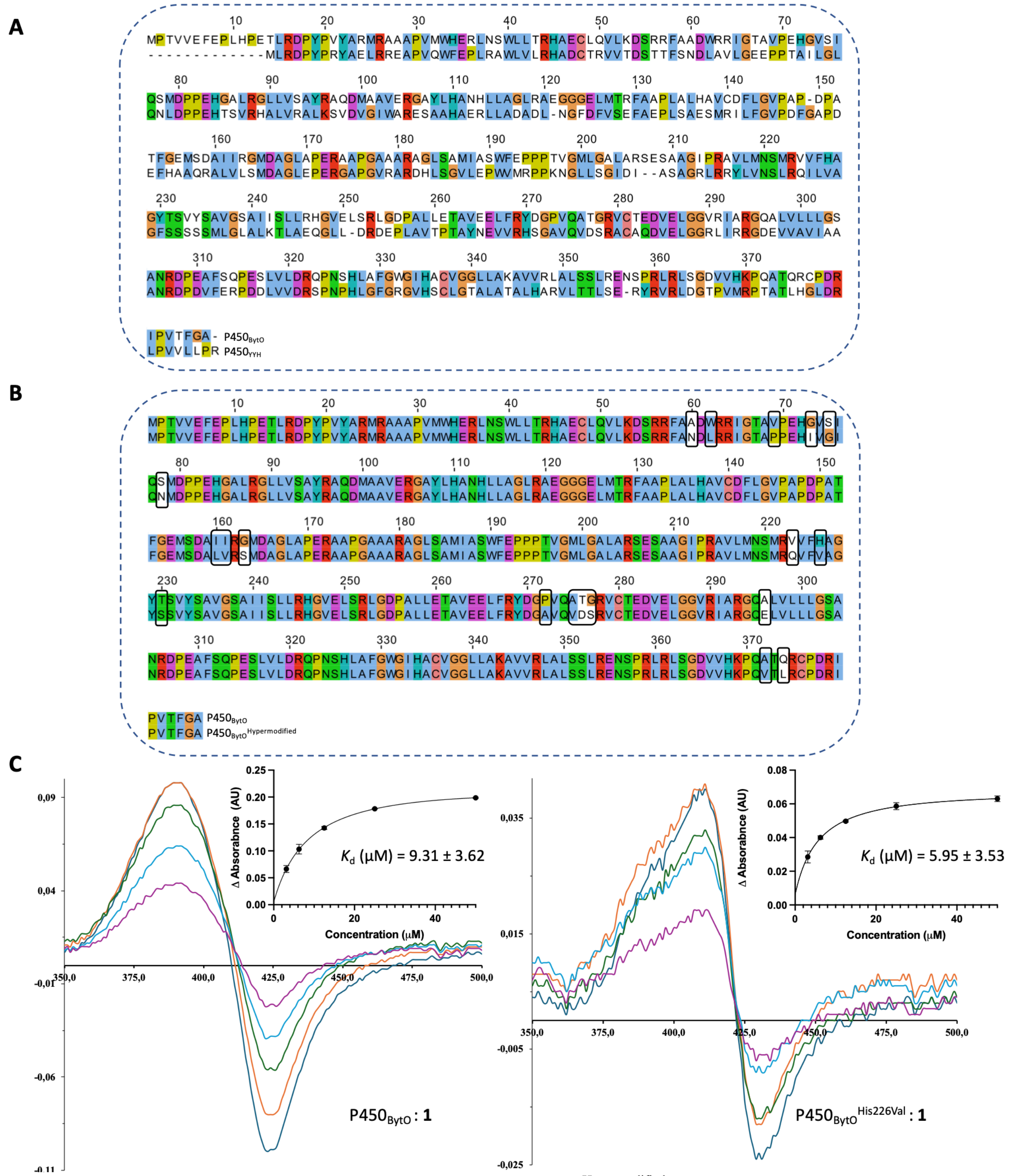

Figure S47. Sequence alignment of $P450_{BytO}$, $P450_{YYH}$ and $P450_{BytO}^{Hypermodified}$. **A**) Sequence alignment of $P450_{BytO}$ and $P450_{YYH}$.**B**) Sequence alignment of $P450_{BytO}$ and $P450_{BytO}^{Hypermodified}$. The mutations are highlighted (black box) Alignments were performed using Clustal Omega[23] and visualised using Jalview.[24] **C**) UV-visible spectroscopic investigation of the binding of $P450_{BytO}$ (left) and $P450_{BytO}^{His226Val}$ (right) to **1**. K_d values are calculated as discussed in the method section.

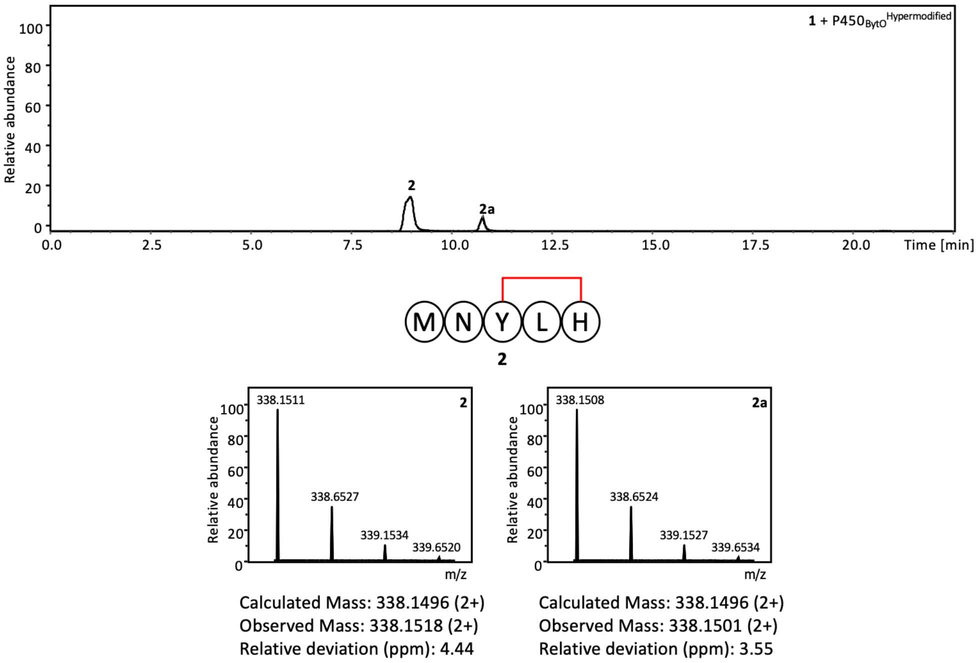

Figure S48. Functional analysis of P450$_{BytO}$^{Hypermodifed}. Incubation of **1** with P450$_{BytO}$^{Hypermodified} resulted in appearance of two peaks (-2 Da loss) with near identical mass but distinct retention times. EICs of **2** and **2a** are shown and the observed and calculated mass values are displayed below. Due to low yields, full structural characterization of **2a** was not possible. HR-MS/MS analysis of **2a** can be found in Figure S49.

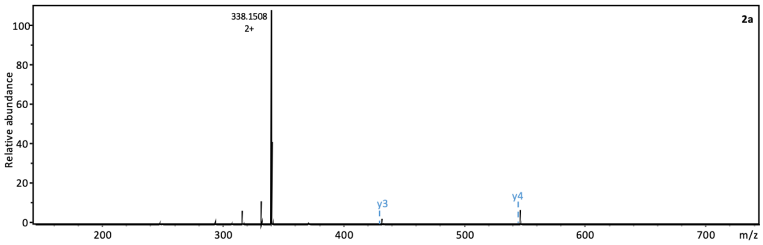

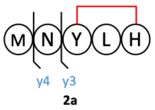

	Observed Mass m/z	Expected Mass m/z	Relative deviation (ppm)
y3	430.2085	430.2079	1.40
y4	544.2521	544.2509	2.20

Figure S49. Characterization of **2a**. HR-MS/MS analysis of **2a** is shown and the *b* and *y* ions are listed in the table and marked in the spectrum. The HR-MS/MS analysis suggest YxH biaryl linkage shown as red square bracket.

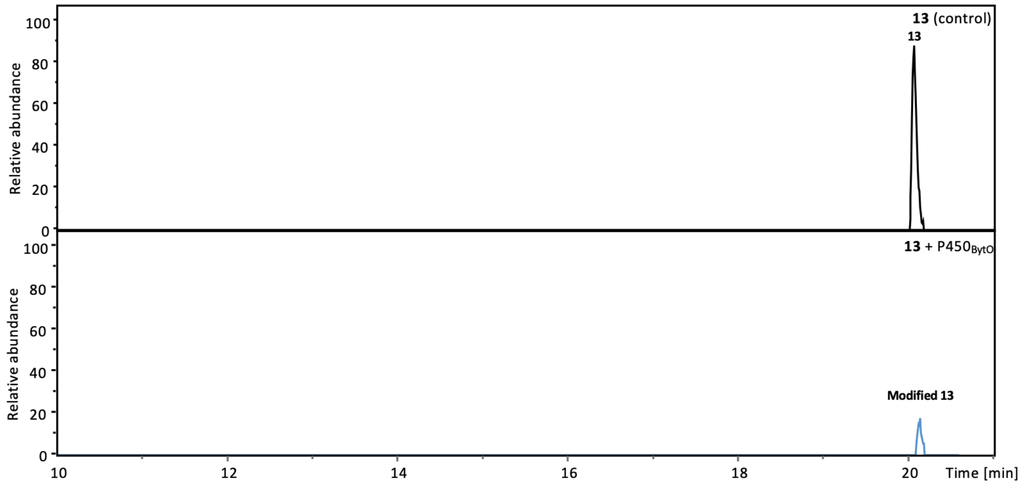

Figure S50. Characterization of modified **12**. EICs of modified and unmodified **12** when incubated with $P450_{BytO}$ are shown. The observed and the calculated masses can be found in Figure S16A. Measurements performed using varying HPLC-gradients also showed a single product peak (data not shown).

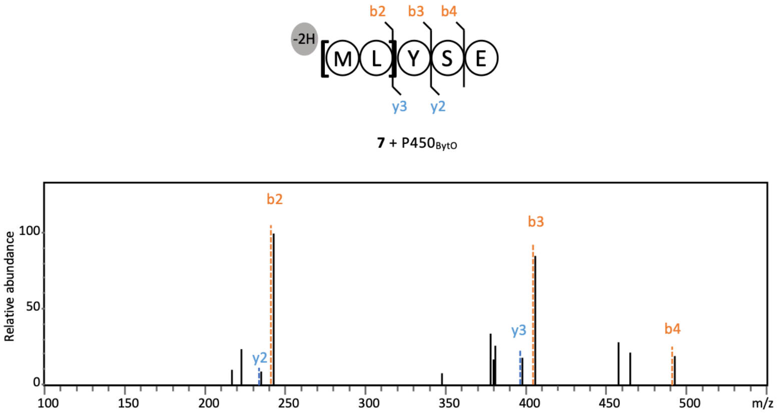

	Expected Mass m/z	Observed Mass m/z	Relative deviation (ppm)
b2	243.1167	243.1154	-4.66
b3	406.1801	406.1785	-3.93
b4	493.2121	493.2164	8.72
y2	235.0930	235.0938	3.40
y3	398.1563	398.1574	2.76

Figure S51. Characterization of modified **7**. HR-MS/MS analysis of modified **7** is shown and the *b* and *y* ions are listed in the table and marked in the spectrum.

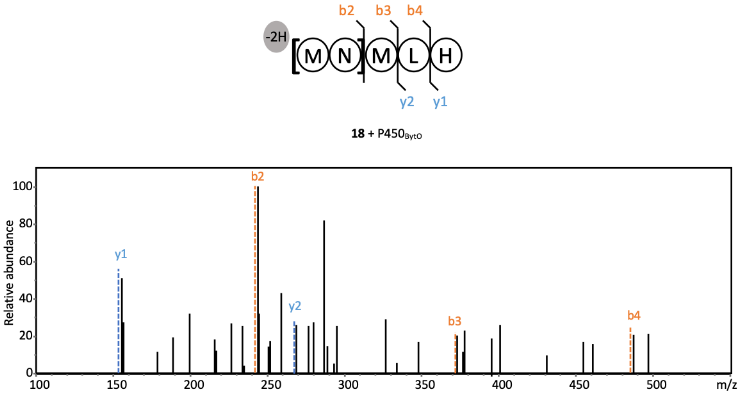

	Expected Mass m/z	Observed Mass m/z	Relative deviation (ppm)
b2	244.0756	244.0742	-5.74
b3	375.1161	375.1200	10.40
b4	488.2001	488.2033	6.55
y1	156.0762	156.0772	6.40
y2	269.1603	269.1598	-1.86

Figure S52. Characterization of modified **18**. HR-MS/MS analysis of modified **18** is shown and the *b* and *y* ions are listed in the table and marked in the spectrum.

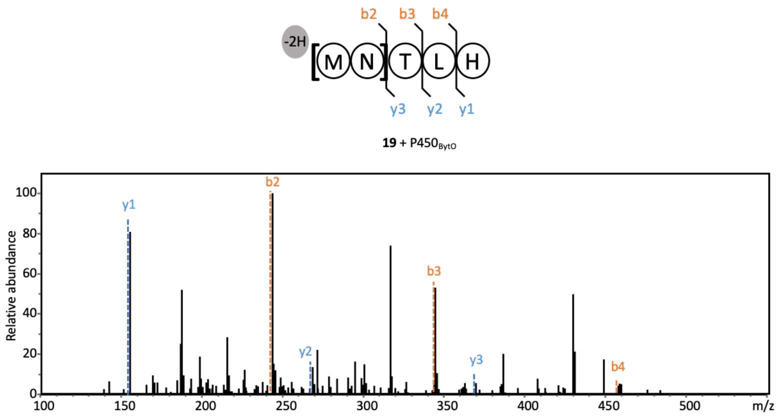

	Expected Mass m/z	Observed Mass m/z	Relative deviation (ppm)
b2	244.0756	244.0747	-3.69
b3	345.1233	345.1238	1.45
b4	458.2073	458.2068	-1.09
y1	156.0762	156.0770	5.12
y2	269.1603	269.1614	4.09
y3	370.2079	370.2082	0.81

Figure S53. Characterization of modified **19**. HR-MS/MS analysis of modified **19** is shown and the *b* and *y* ions are listed in the table and marked in the spectrum.

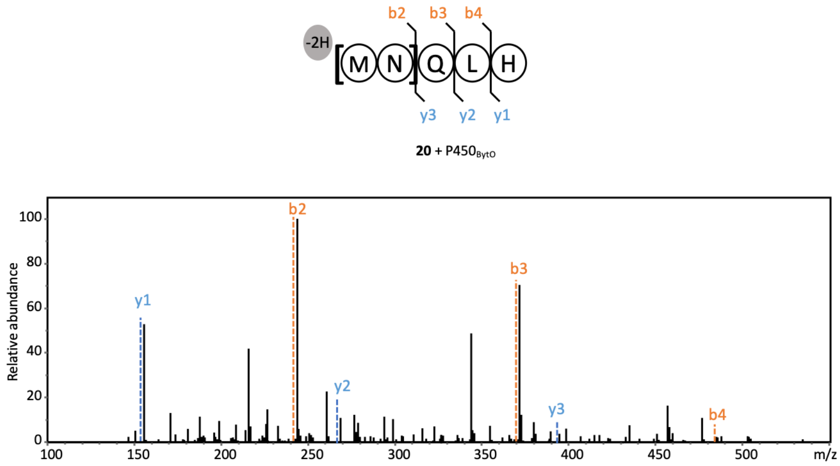

	Expected Mass m/z	Observed Mass m/z	Relative deviation (ppm)
b2	244.0756	244.0757	0.41
b3	372.1342	372.1332	-2.69
b4	485.2182	485.2152	-6.18
y1	156.0762	156.0762	0.00
y2	269.1603	269.1612	3.34
y3	397.2188	397.2199	2.77

Figure S54. Characterization of modified **20**. HR-MS/MS analysis of modified **20** is shown and the *b* and *y* ions are listed in the table and marked in the spectrum.

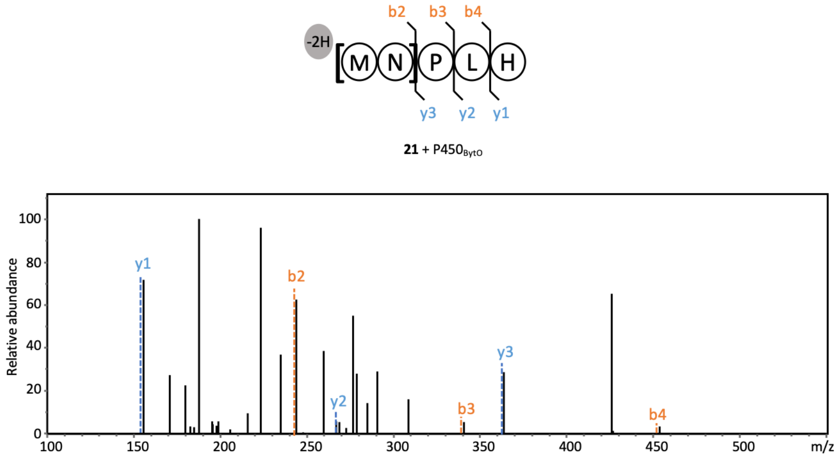

	Expected Mass m/z	Observed Mass m/z	Relative deviation (ppm)
b2	244.0756	244.0757	0.41
b3	341.1284	341.1279	-1.47
b4	454.2124	454.2118	-1.32
y1	156.0762	156.0761	-0.64
y2	269.1603	269.1597	-2.23
y3	366.2130	366.2135	1.37

Figure S55. Characterization of modified **21**. HR-MS/MS analysis of modified **21** is shown and the *b* and *y* ions are listed in the table and marked in the spectrum.

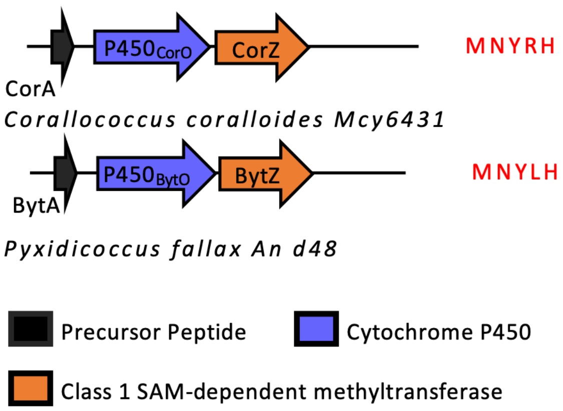

Protein	Sequence identity
P450$_{CorO}$/P450$_{BytO}$	91.69% (100% coverage)
CorZ/BytO	93.18% (100% coverage)

Figure S56. Putative biarylitide-like BGC from *Corallococcus coralloides*. A BGC highly homologous in sequence and organization to myxarylin producer (*pyxidicoccus fallax An d48*; NCBI GenBank: OL539738.1) was found in an in-house strain library (*corallococcus coralloides MCy6431*). The precursor peptide sequences are shown on the right (red) and the sequence identity at the bottom. The sequence of *cor* BGC can be found in Table S1.

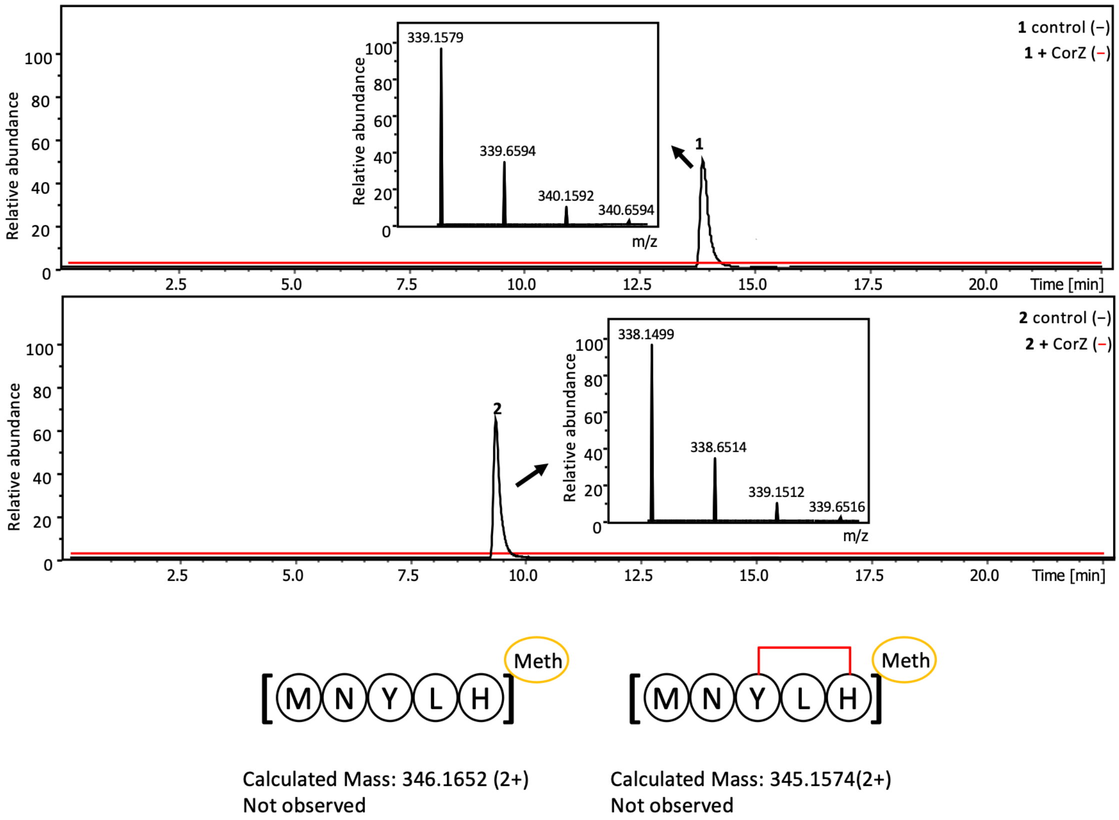

Figure S57. Incubation of **1** and **2** with CorZ. EIC analyses of **1** and **2** (control, –) and after incubation with CorZ in the presence of excess SAM (–). We failed to observe a peak corresponding to approx. + 14 Da, a mass increase expected for methylation. HPLC-ESI-MS analyses (inlet) along with the expected masses are shown (bottom).

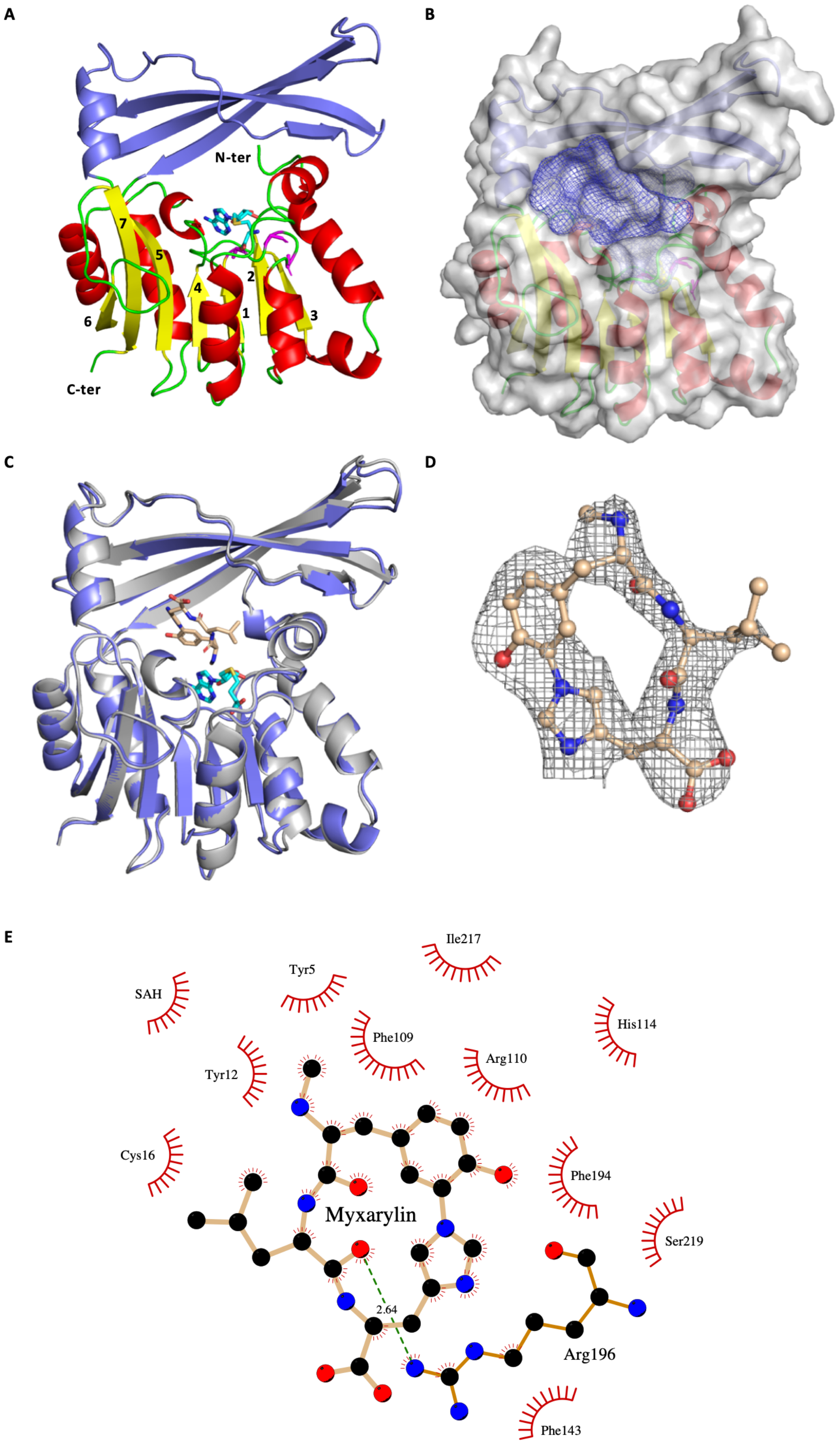

Figure S58. Structural analysis of CorZ. **A**) Cartoon representation of the CorZ monomer highlighting the core Rosmann fold (colour scheme: α-helices, red; β-strands 1-7, yellow; and loops, green) and the additional insert (slate) that forms a lid over the substrate-binding pocket. The conserved GxGxG motif located close to the SAH (cyan) is depicted as pink sticks. **B**) Substrate-binding pocket of CorZ shown as mesh (blue). Total volume of the pocket was calculated to be approx. 1100 Å^3 by CavitOmix (v.10.2022, Innophore Gmbh). **C**) Superposition of CorZ-SAH (grey) with CorZ-SAH-myxarylin (slate) structure. Cα RMSD was approx. 0.30 Å over the entire length of the protein. SAH (pink) and myxarylin (wheat) are shown as sticks **D**) Polder map (grey isomesh) of myxarylin contoured at 3.0σ. **E**) Ligplot diagram of the interactions between myxarylin with CorZ and SAH. Atom are represented as circles (carbon black, oxygen red, nitrogen blue). Hydrogen interactions are shown as dashed green line with distance given in Å; whereas, the hydrophobic interactions are depicted as red spoked arcs.

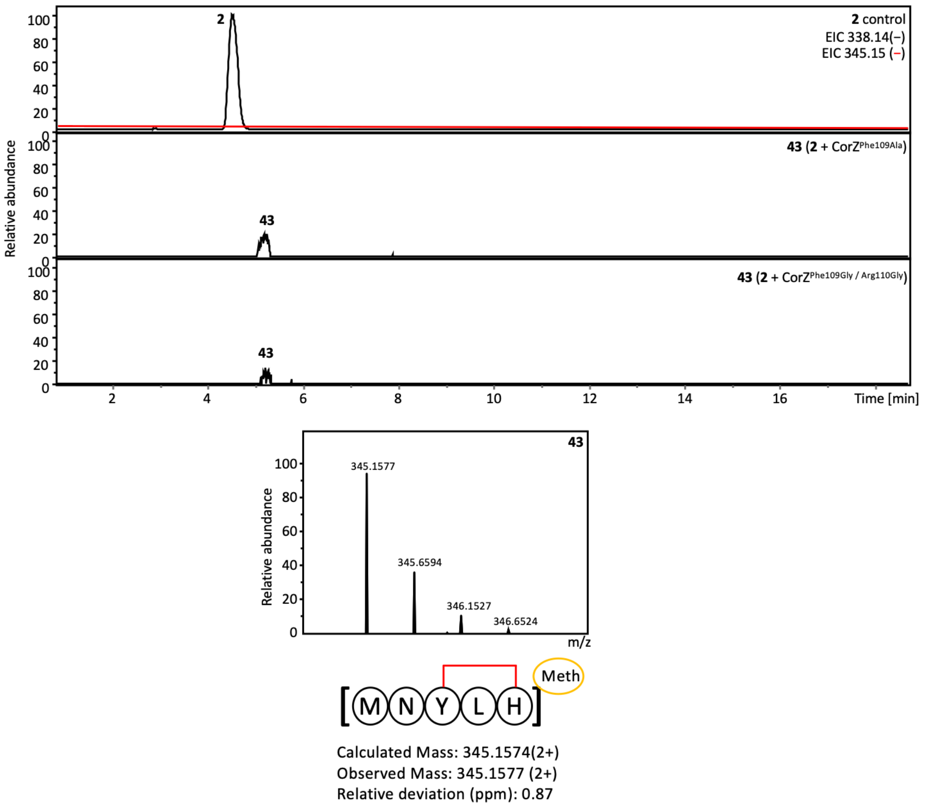

Figure S59. Characterization of **43**. EIC analysis of **2** control (unmethylated, EIC 338.14 ± 0.1; and methylated EIC: 345.15 ± 0.) after incubation with excess SAM is shown at the top. Incubation of **2** in the presence of CorZ^{Phe109Ala} (middle) or CorZ$^{Phe109Gly / Arg110Gly}$ (bottom) and SAM leads to the appearance of **43**. HPLC-ESI-MS characterization of **43** along with the observed and calculated mass values are shown.

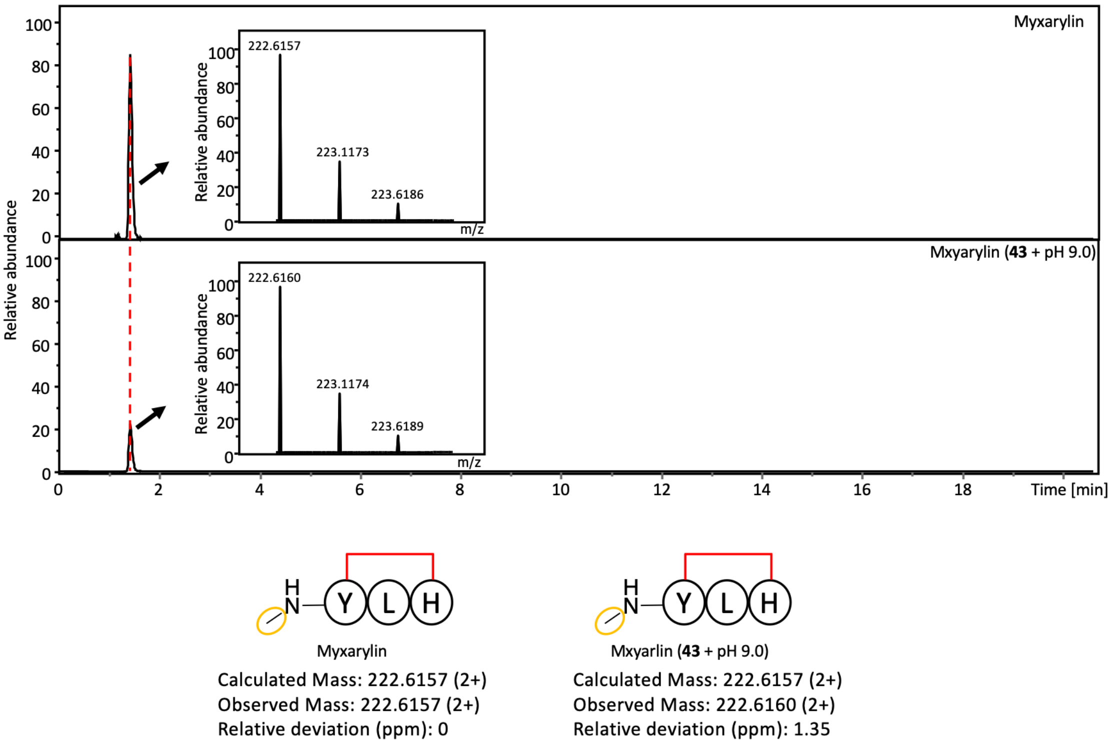

Figure S60. Incubation of **43** under basic condition. Freeze-dried **43** was resuspended in Tris pH 9.0, and overnight incubation at 37 °C resulted in accumulation of a peak with mass and retention time similar to that of purified myxarylin, supporting N-terminal methylation by CorZ mutants (Figure S59). HPLC-ESI-MS characterization of myxarylin along with the observed and calculated mass values are shown.

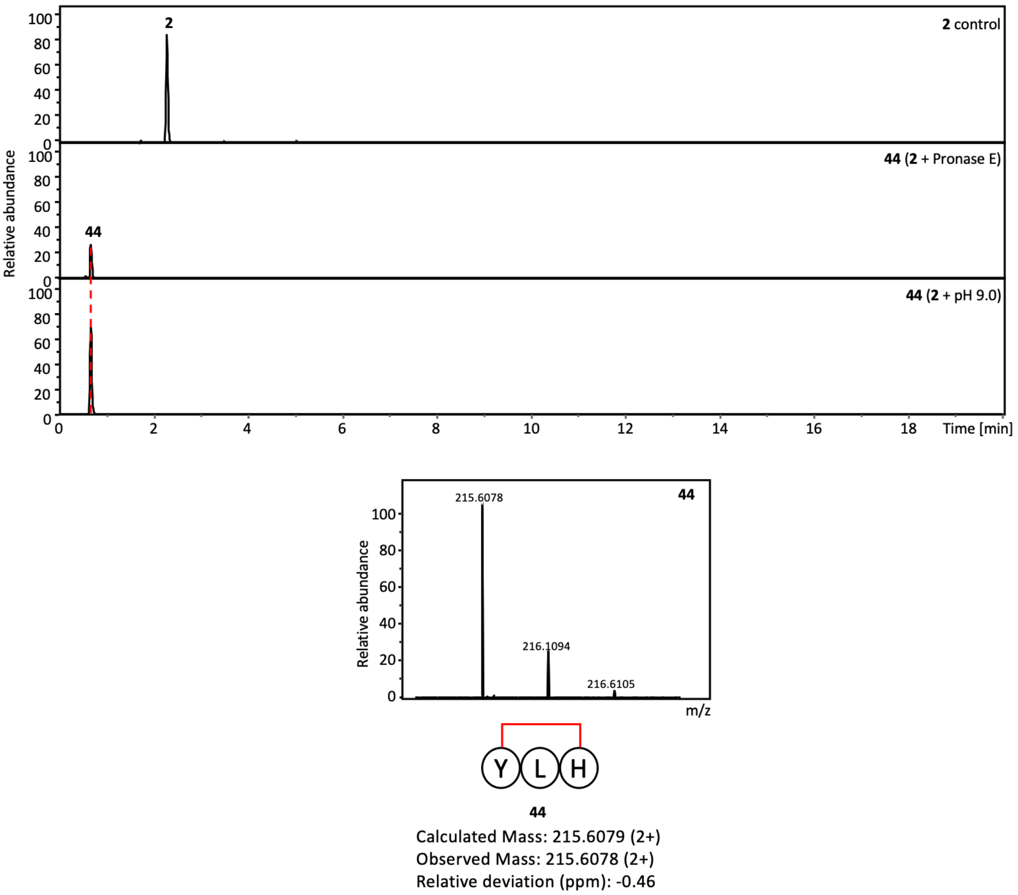

Figure S61. Characterization of **44**. EIC analyses of **2** control and samples after treatment with either pronase E or incubation under basic conditions (pH 9.0). Compared to pronase E treatment, significantly higher yields were observed following incubation at pH 9.0. HPLC-ESI-MS analysis along with the observed and calculated mass values are shown.

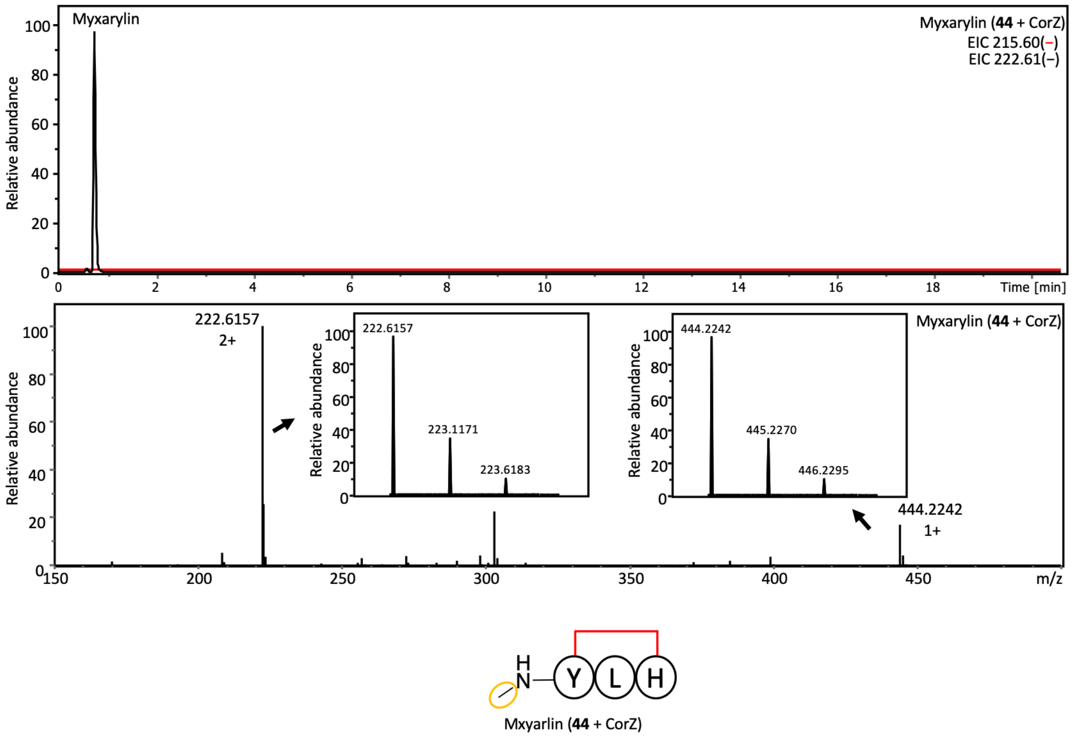

Calculated Mass: 222.6157 (2+)
Observed Mass: 222.6157 (2+)
Relative deviation (ppm): 0

Figure S62. Characterization of *in vitro* reconstituted myxarylin. EIC analysis of **44** after treatment with CorZ in the presence of excess SAM. We observed complete consumption of **44** (EIC 215.60 ± 0.1, –) and subsequently appearance of a new peak with + 14 Da (methylation; EIC 222.61 ± 0.1, –). HPLC-ESI-MS analyses along with the observed and calculated mass values are shown.

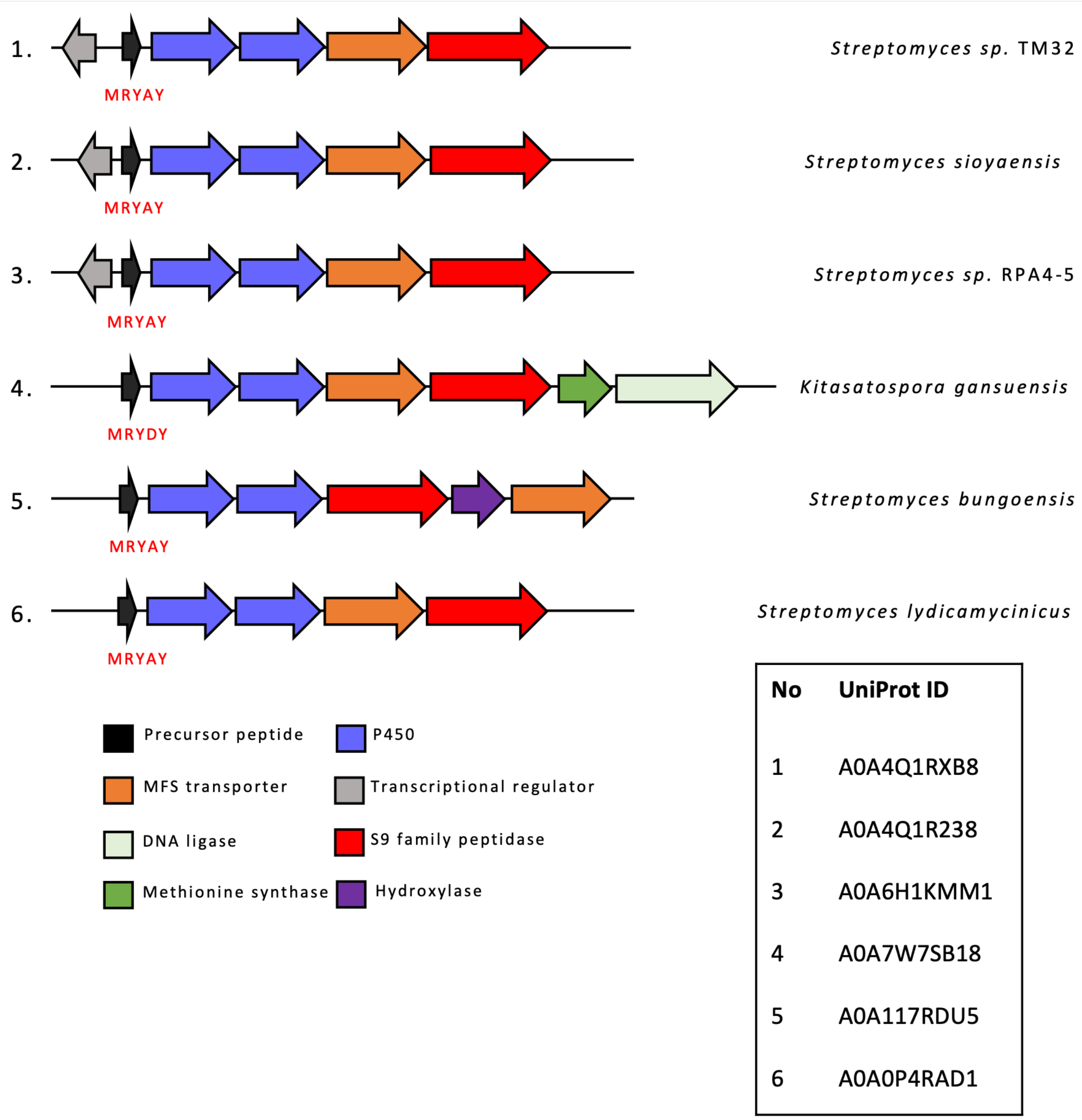

No	UniProt ID
1	A0A4Q1RXB8
2	A0A4Q1R238
3	A0A6H1KMM1
4	A0A7W7SB18
5	A0A117RDU5
6	A0A0P4RAD1

Figure S63. Biarylitide-like BGCs containing putative prolyl oligopeptidase. The identified proteases are annotated as putative prolyl oligopeptide family serine proteases (POPs; pf00326), a subset of S9 protease family. Uniport IDs of the proteases are shown. The precursor peptides associated with the BGCs were identified manually and are the corresponding sequence highlighted in red.

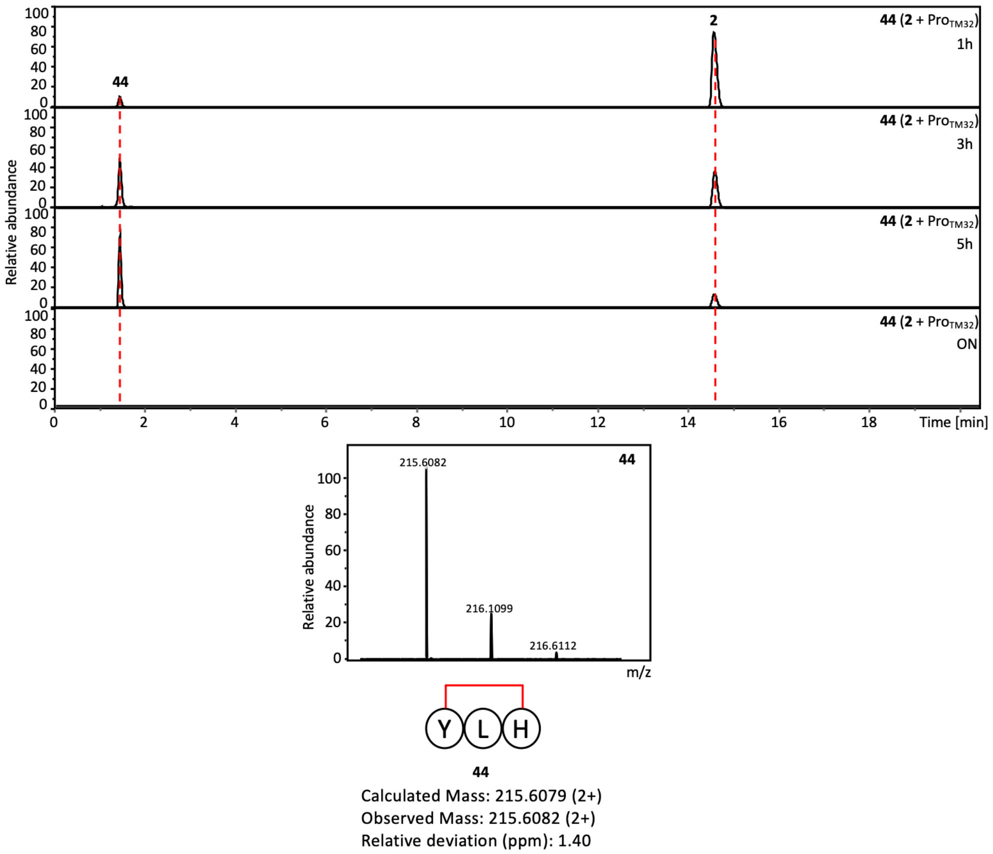

Figure S64. Time-course analysis of Pro$_{TM32}$ activity on **2**. EICs of **2** and **44** are shown with sampling points indicated. After overnight incubation, nearly all of **2** and **44** were consumed. To improve the separation, a modified LC-MS gradient was used (see LC-MS and MS/MS analysis).

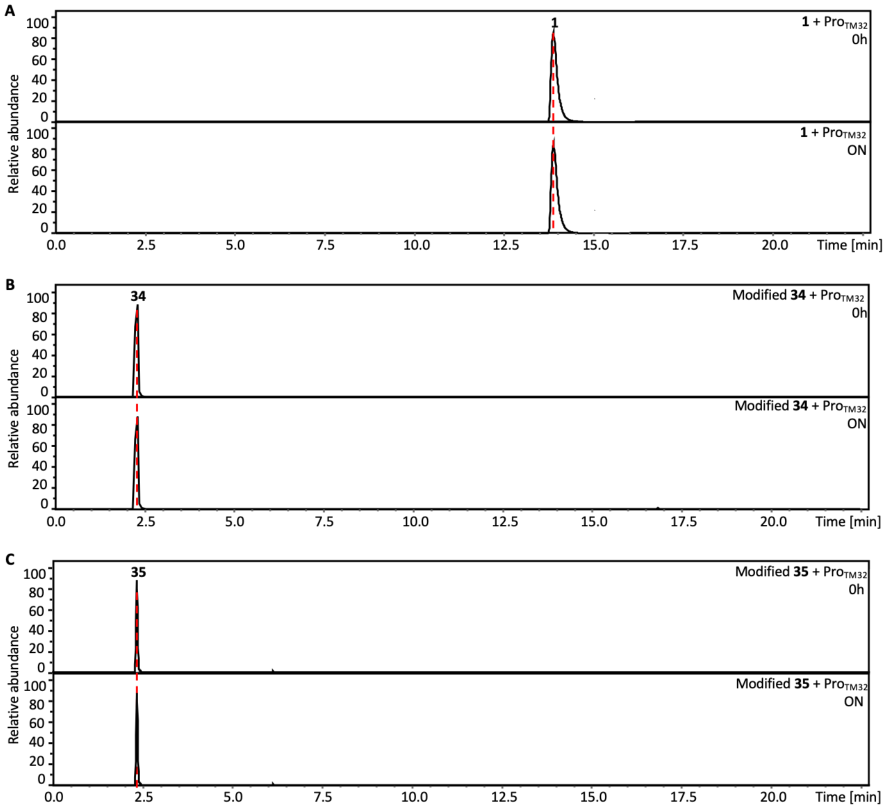

Figure S65. Time-course analysis of Pro$_{TM32}$ activity on **1**, **34** and **35**. EICs of **1** and modified **34** - **35** are shown with the corresponding sampling points indicated. No proteolytic degradation was observed following overnight incubation.

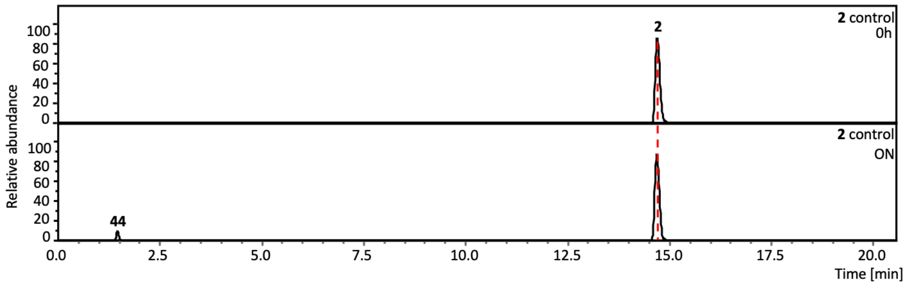

Figure S66. Time-course analysis of **2** stability. EIC of **2** is shown with the corresponding sampling points indicated. In the absence of Pro$_{TM32}$, minimal degradation was observed after overnight incubation in protease reaction conditions.

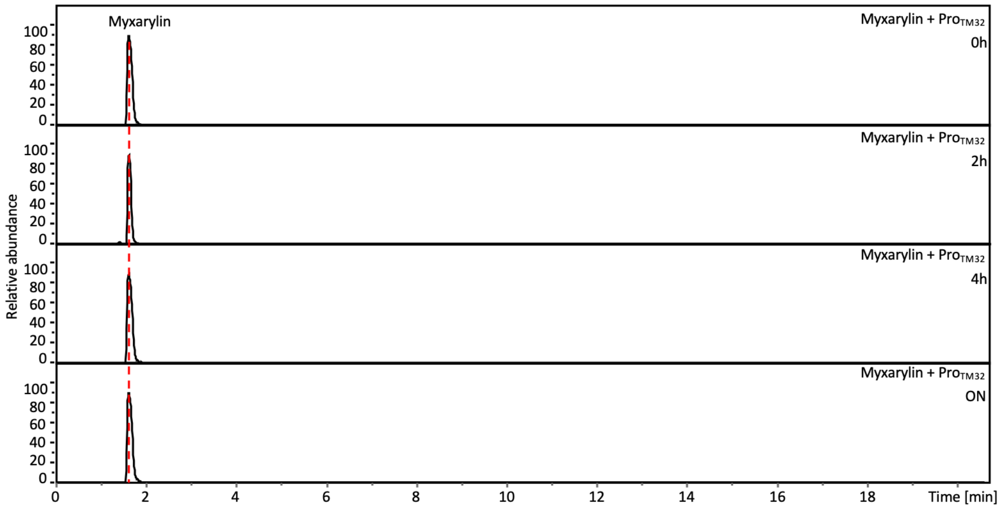

Figure S67. Time-course analysis of Pro$_{TM32}$ activity on myxarylin. EICs of myxarylin is shown with the corresponding sampling points indicated. No proteolytic degradation was observed following overnight incubation.

NMR Spectra and Figures

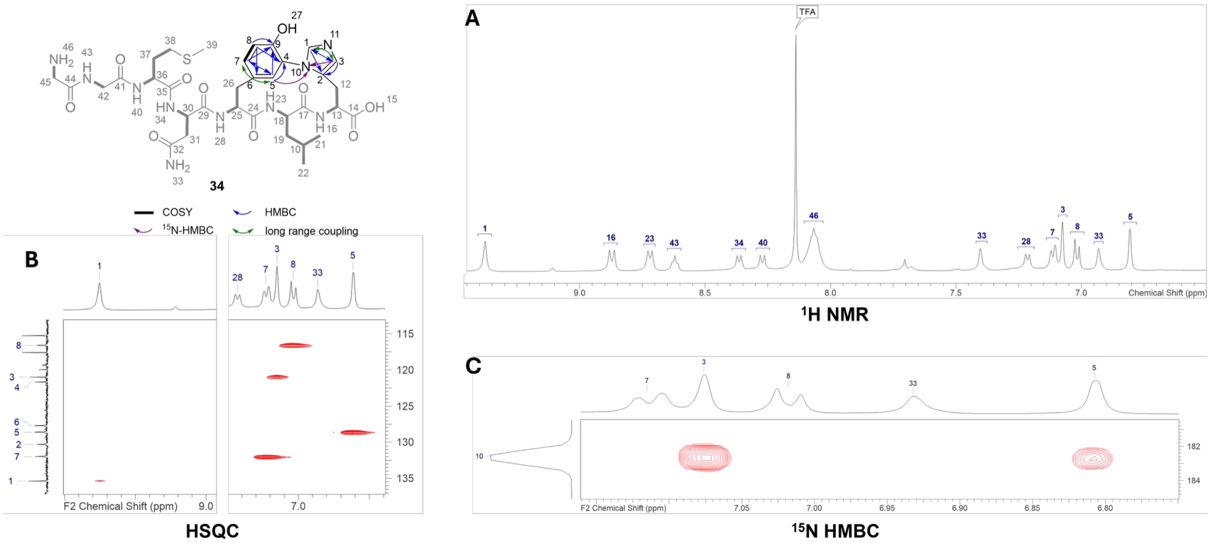

Figure S68. A) ^{1}H NMR spectrum of the aromatic signals of **34** in DMSO-d_6 (0.2% TFA). **B**) HSQC spectrum of the aromatic signals of **34** in DMSO-d_6 (0.2% TFA). (**C**) HSQC signals of the aromatic of **34** in DMSO-d_6 (0.2% TFA). (C) ^{15}N-HMBC key correlations between Tyr-C5 and His-N10 and His-N3.

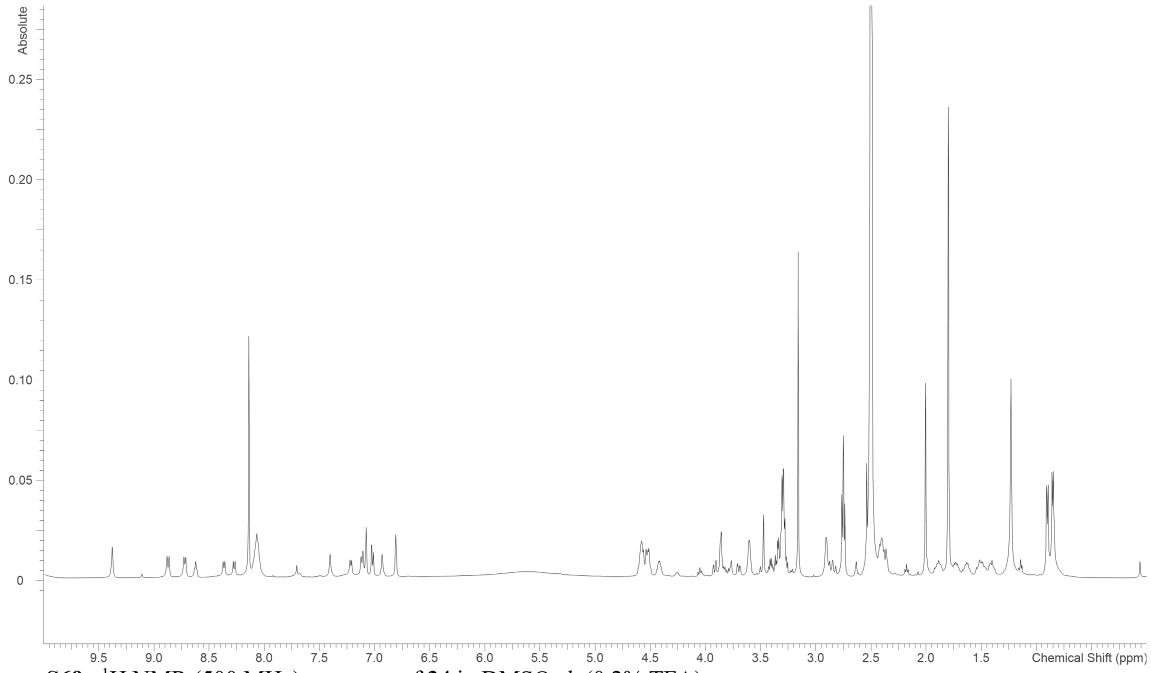

Figure S69. ^{1}H NMR (500 MHz) spectrum of **34** in DMSO-d_6 (0.2% TFA).

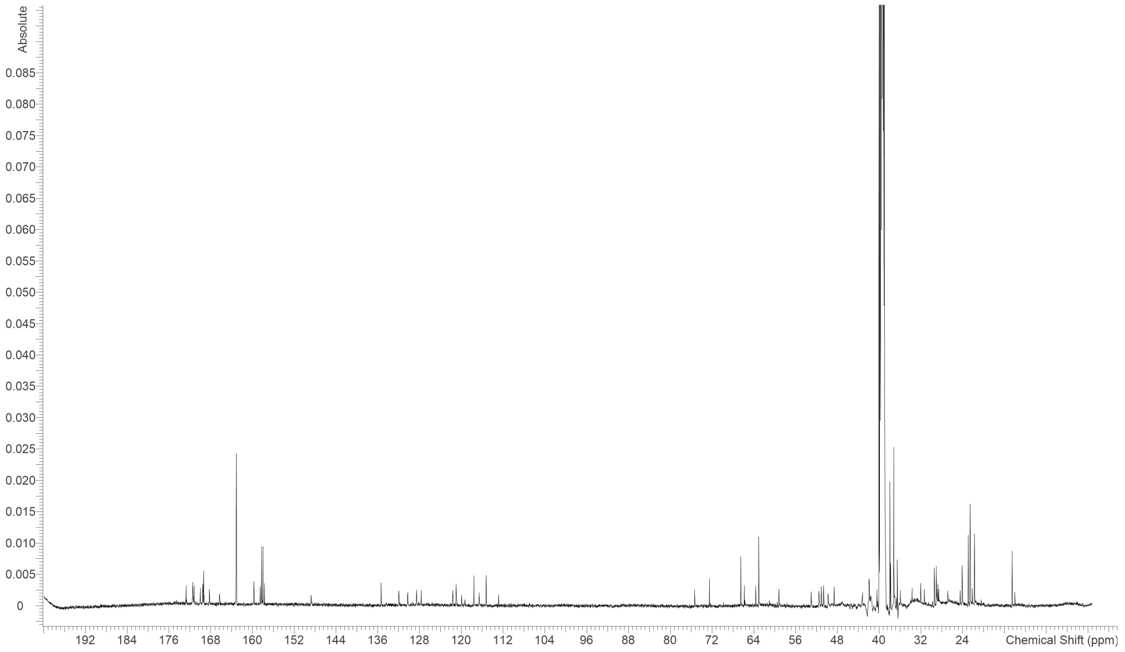

Figure S70. ^{13}C NMR (500 MHz) spectrum of **34** in DMSO-d_6 (0.2% TFA).

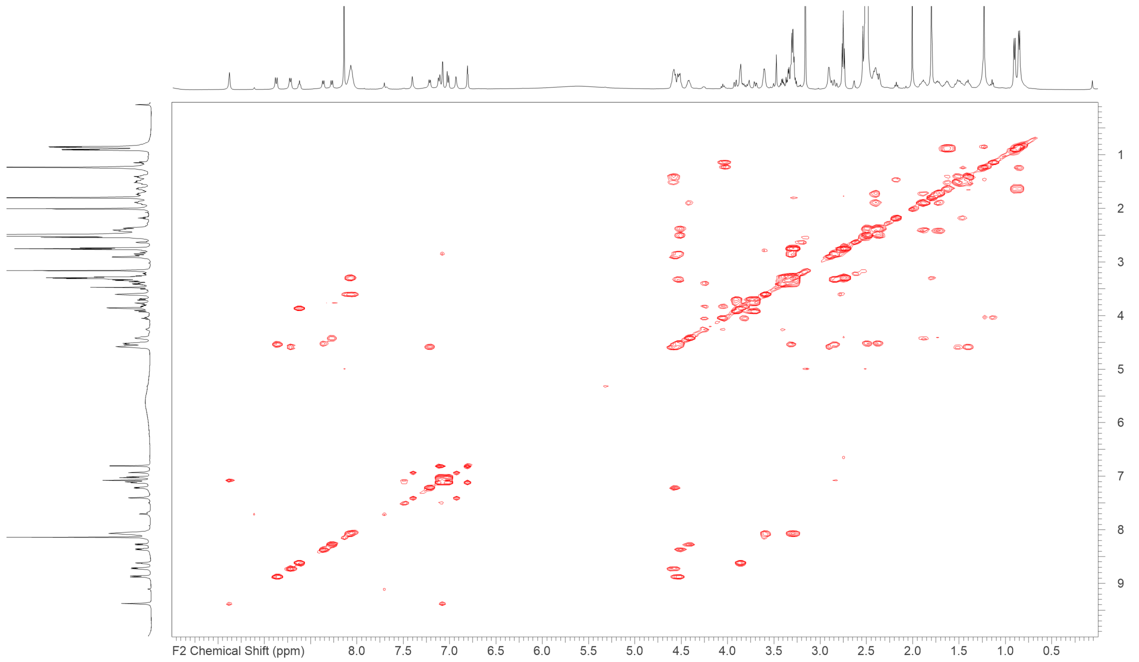

Figure S71. COSY spectrum of **34** in DMSO-d_6 (0.2% TFA).

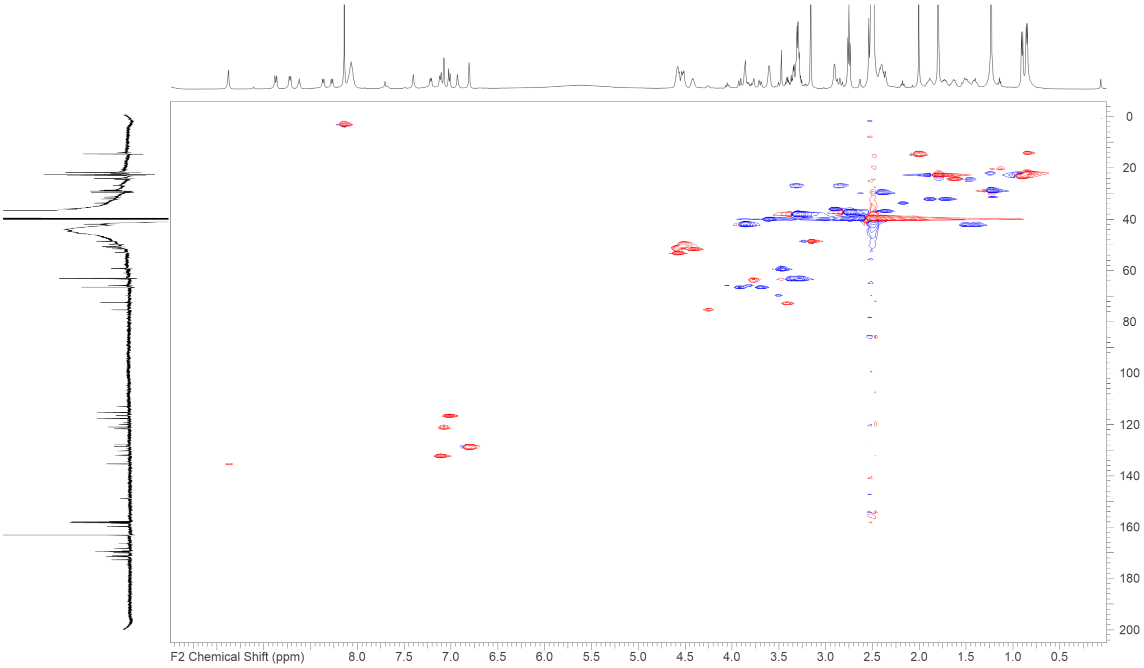

Figure S72. HSQC spectrum of **34** in DMSO-d_6 (0.2% TFA).

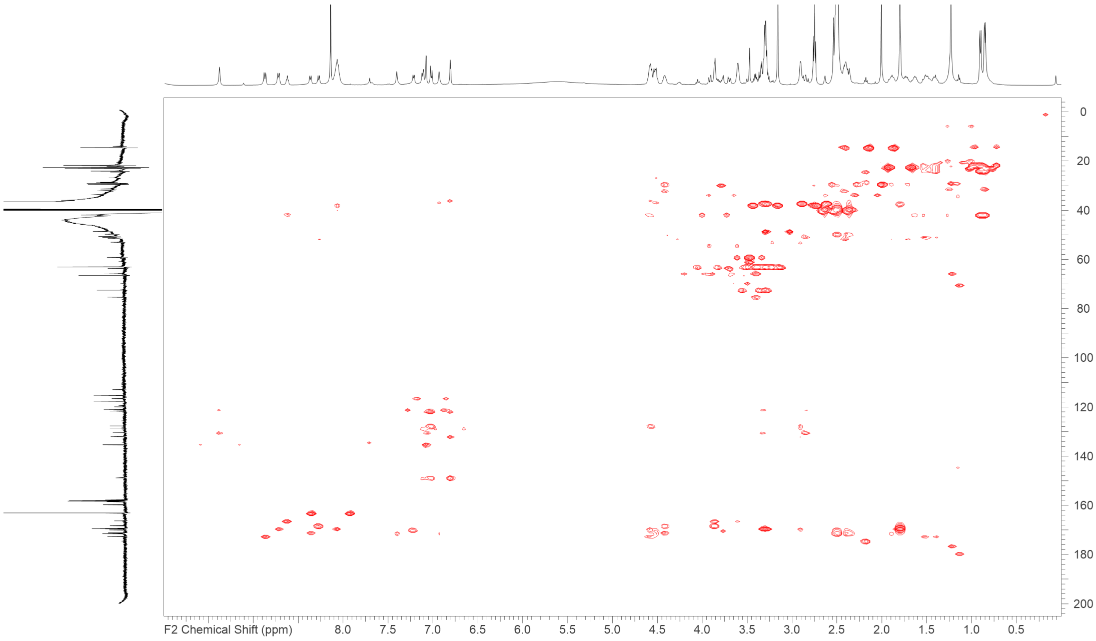

Figure S73. HMBC spectrum of **34** in DMSO-d_6 (0.2% TFA).

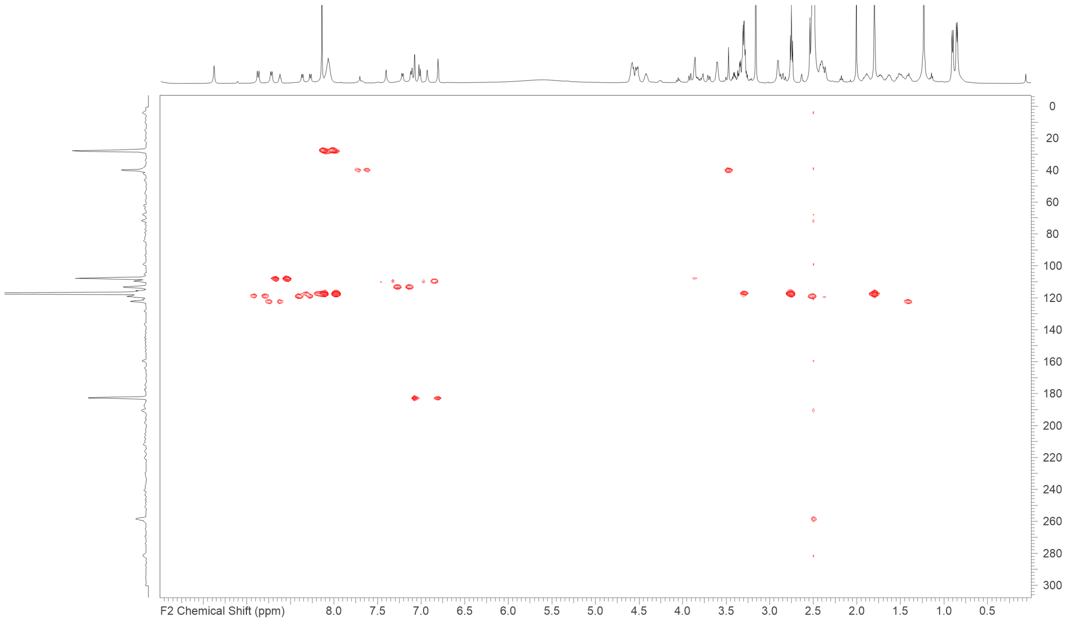

Figure S74. ^{15}N HMBC spectrum of **34** in DMSO-d_6 (0.2% TFA).

Supplementary Tables

Table S1. Sequence of genes of the *cor* BGC, hypermodified P450$_{BytO}$, and Pro$_{TM32}$.

**Gene Name**	**Sequence (5'-to-3')**
corA	ATGAACTACCGGCACTGA
P450$_{corO}$	GTGTCCACTGCTGTCGAGTTCGAGCCCCTGCACCCCGAGACGCTGCGAGACCCGTACC<br>CCGTCTATGCTCGAATGCGGGCGGTGGCGCCGGTGATGTGGCATGAGCGGCTCAACTC<br>CTGGCTGCTCACCCGACACGCGGAGTGTCTCCAGGTGCTGAAGGATTCGCGCCGCTTC<br>GCCGCCGACTGGCGCCGGGTTGGCGCGGCGGTGCCCGAGCACAGCCAGAGCATCCAA<br>TCCATGGACCCGCCCGAGCACGGCGCGCTGCGCGGCCTGCTCGTGAGCGCCTACCGCG<br>CCCAGGACCTGGTTGCCGTGGAGCGCGGGGCCTACCTGCATGCGAATCACCTGCTCGA<br>CGGGCTTCGCTCGGAGGGTGGGGGCGAGCTGATGACGCGCTTCGCGGCGCCGCTCGC<br>ACTCCATGCCGTCTGTGATTTCCTCGGCGTCCGTGCGCCGGACCCCGCCACCTTCGGGG<br>AGATGTCGGACGCCATCATCCGCGGCATGGACGCGGGCCTCGCGCCGGAGCGAGCGG<br>CCCCCGGTGCCGCCGCCGGGGCTGGCCTGAGCGCGCTGATTGCGAGCTGGTTCGAGCC<br>TCCTCCGACGGGTGGAATGCTCGGATTCCTGGCGCGCTCCGAGCCGGCCGCCGGGGTG<br>CCGCGCGCCGTGCTGATGAACAGCATGCGCGTCGTGTTCCATGCCGGCTACACGTCCG<br>TGTACAGCGCGGTGGGCAGCTCCATCATCGCCCTGCTGCGCCATGGCGTGGAGCTGTC<br>CCGGCTGGCCGACCCGGCGCTCCTGGAAACGGCGGTGGAGGAGCTGTTCCGGTATGA<br>CGGCCCGGTGCAGGCCACCGGTCGCGTGTGCACCGAGGACGTGGAGCTCGGTGGGGT<br>CCGCATCCGCCGAGGGCAGGAGCTGGTGTTGCTGCTCGGCTCCGCCAACCGGGACGCG<br>GAGGCATTCTCCCAGCCGGAGACGCTCGTCCTGGATCGCCAGCCCAACCCCCACCTGG<br>CCTTTGGTTGGGGCATTCATGCCTGCGTGGGCGGGCTGCTGGCCAAGTCGGTGGTGCG<br>GCTGGCCCTGGCCAGCCTCATCGAGCACGCACCGCGCCTGCGCCTGAGCGGTGACGTC<br>GTCCACAAGCCCCAGGCCACCCAGCGGTGCCCGGACCGCATTCCCGTCTCGTTCGGCA<br>CGTGA
corZ	GTGGACTTCGACTACACCGACGATGCGGCCCTCTATGACGCCGTTTGCGAGGACTACC<br>GGGAGGACGTCGCCTTCTACGTGGAGGAAGCCCGGGGCGCGGGTGGCCCCTGCCTGG<br>AGCTGGGCTGTGGCACCGGGCGGCTCCTCACTCCGGCGGTGGAGGCCGGCGCGCGCG<br>TCACGGGGCTGGACCGCTCGGCGGCGATGCTCGCCCGGGCCCGAGCGCGGGTCCAGG<br>CCCTGCCTGCTCCTCTTCGGGAGCGGGTCGACCTGCGCGAGGGCGACATGGTGTCGTT<br>CTCGCTCGAAGCGCGCTTCGCGCTCATCACCGTGCCCTTCCGCACCTTCCTCCACCTGC<br>TCACGGTGGAGGAGCAGCTCGCGGCGCTCACCAACATCCGCCGGCACCTGCTACCGG<br>GCGGGCGGCTCGTGCTCGACTTCTTCGAGCCGTCGCGGCTGCTGGCCGAGCTGCTCGG<br>CAATGACGGCCCGTCCCGGGGGCTGCTGAAGCAGACGGGGGTTGTCGTCTCGCATCCG<br>GTGACGGGCAACATGCTCGTCGAGTGGGCCAGCGTGACGGGGGACCCCGTGAGCCAG<br>TGCTTCACCCGCTGCCTCGTGTACGACGAGCTGGAGCGCTCCGGGCAGGTGGTGGGGC<br>GGATGTACCGGAGAATCACCAGTCGCTTCATCTTCCGCTCGGAGTTCGAGCACCTCCT<br>GCACCGCTCCGGCTTCCAGGTGGAGGCGCTCCAGGGGTCGTTCGACGGCGGCCCGGTC<br>CGCCCGGGCGGAGAGCTCATCTGGCGGGCCCGCGCCGCGCCGTGA
P450$_{BytO}$^{Hypermodified}	ATGGTGCCCACCGTCGTCGAGTTCGAGCCCCTGCACCCCGAGACGCTGCGAGACCCCT<br>ACCCCGTCTATGCCCGGATGCGGGCGGCGGCGCCGGTGATGTGGCATGAGCGGCTCA<br>ACTCCTGGCTGCTCACCCGGCACGCGGAGTGTCTCCAGGTGCTGAAGGATTCACGCCG<br>CTTCGCCAACGACCTGCGAAGGATTGGCACGGCGCCGCCCGAGCACATCGTGGGCATC<br>CAAAATATGGACCCGCCCGAGCACGGCGCGCTGCGCGGCCTGCTCGTGAGCGCCTACC<br>GCGCGCAGGACATGGCGGCGGTGGAGCGCGGGGCCTACCTGCACGCCAATCACCTGC<br>TCGCCGGGCTTCGCGCGGAGGGCGGGGGCGAGCTGATGACGCGCTTCGCGGCGCCGC<br>TCGCGCTCCATGCCGTCTGTGATTTCCTCGGTGTCCCCGCGCCGGACCCCGCCACCTTC<br>GGGGAGATGTCGGACGCCCTGGTCCGCTCTATGGACGCGGGCCTCGCGCCGGAGCGA<br>GCGGCCCCCGGCGCCGCCGCCAGGGCGGGCCTGAGCGCGATGATTGCGAGCTGGTTC<br>GAGCCCCCTCCGACAGTGGGGATGCTCGGGGCCCTGGCGCGCTCCGAGTCCGCCGCCG<br>GGATTCCGCGGGCCGTGCTGATGAACAGCATGCGCCAGGTGTTCGTGGCCGGCTACTC<br>TTCCGTATACAGCGCGGTGGGCAGCGCCATCATCTCCCTGCTGCGCCACGGCGTGGAG<br>CTGTCCCGGCTGGGAGACCCGGCGCTCCTGGAGACGGCGGTGGAGGAGCTGTTCCGGT<br>ATGACGGCGCGGTGCAGGTGGATTCTCGCGTGTGCACCGAGGACGTGGAGCTTGGCG<br>GCGTCCGCATCGCGCGGGGACAGGAACTGGTGCTGCTGCTGGGCTCGGCCAACCGGG<br>ACCCGGAGGCGTTCTCCCAGCCGGAGTCGCTCGTCCTGGACCGGCAGCCCAACTCCCA<br>CCTGGCCTTCGGCTGGGGCATCCACGCCTGCGTGGGCGGACTGCTGGCCAAGGCGGTG<br>GTGCGGCTGGCCCTGTCCAGTCTCAGGGAGAACTCACCGCGCCTGCGCCTGAGCGGTG

	ACGTCGTCCACAAGCCCCAGGTGACCCTGCGGTGCCCGGACCGCATTCCTGTCACATT<br>CGGCGCGTGA
Pro$_{TM32}$	ATGGTAGATGGTGTAGATGATGAGAGGTGGAAAGCACGTATCGGAGTGGCCCATGTG<br>ACGGTACCCCGCTGGGCACGTGATGCCCCGGACCGCTGTGTCTACCGTTCTAACGCCA<br>CAGGGACGTGGGAGTTGTATGAGTGGGATCGGGCGAGCGATGCTCGTCGCCGTCTTAC<br>GGATCGCCCTCATGGGACGGCGTTGGGGTTTATTGATCCTGCCGGTGATTGGGTGTGG<br>TGGTTTGCCGATACTGGCGGAGACGAATGCGGTGTTTGGATGCGACGTCCGTTCGGCG<br>GAGAACATGAAGAACCGGCTGTGCCTGGTCTGGACCCAGCCTACCAAGCAGGCCTGG<br>CCTTGGGTGCGAATGGCCTCGCAGTGTTAGGGACAAGTGTGACCGGCGGGACAACTGT<br>ACATGTTTGCCGCCCGGGAACCGCACCACGCACGCTGTACACCCACCCGTCGGATGCC<br>CACGTGGGGGCCATGTCCGCTGATGATACCCTGGTCGCGATTGGCCACAGCGAACACG<br>GCGATAGCAGACACAAAGCGGTGCGTGTTCTGAGCCTAGATGGTAGGGTTGTAGCGG<br>ACCTGTGGGACGGGGAAGGTCGTGGCCTTGGTGGTATTTGGGATGGCCCGGGTAAAG<br>GCTTGGGCGGAATGGCGTTCAGTCCGGTCCCGGGTGACAATCGCCTGCTCATCGAACA<br>CGAACGCCGAAGCCGGCCAGAACCGATGATTTGGGACGTCGCCACGGGTGAGCAGCG<br>CGAATTAGCGCTCGATCTGCCGGGCGAGGTCAGCGCGGCGTGGTATCAGGATGGCTCG<br>GCGTTGCTGATATGGCATACCCATCACGCTCGTGATGAGCTGTACCGGCTGGAACTGG<br>GCACCGCGGAAGCCCGCGGCCTGACACGCTTAGACACGCCGAAAGGTGTCATTCGGG<br>GCGCCGGTACCCGTCCGGATGGCTCAGTGGAATTTGTCTGGTCGAGCGCAGCGCATCC<br>CCCGGTCGTTCGATCCACCTCTGGTGGCACTGTACTGGCCCCGCCTGGTCCTGCTGCTC<br>CTCCGTCCGTTCCGGTGACCGATGCTTGGGTGGAAGGTCCCGGGGGACGCATCCATGC<br>GTTGGTATCACGTCCGCCTGGAGCAGATGGTCCGCTTCCGTGCGTTTTCGAAGTACAT<br>GGCGGTCCGATTGGTTACGACGATGATGCCTTTGACCCAGTGGTTGCGGCATGGGTTG<br>ACCATGGCTTCGCGGTCGTCCAGGTGAATTACCGTGGTTCCACGGGCTATGGCTCCCG<br>TTGGCGTGATGCGATTGAGGGTCGCCCAGGCCTGACGGAGCTCGAAGATATCGAAGC<br>CGTAAGAGCATGGGCAGTAGCCAGCGGGCTGGCGGCTCCGGGACGTTTAGTTCTGTCT<br>GGCCGTTCATGGGGTGGTTATCTTACCCTGTTAGGGCTCGGTACCCGCCCAGGGAATT<br>GGGCTGTTGGCATTTCAACTATGCCAGTGGCGGATTATTTCGCAGCCTATGAGGATGA<br>GATGGAAGCGCTGCGCGCGTACGACCGCTCGCTGTTTGGCGGCAGTCCAGATGACGTT<br>CCCGAACGGTACCGTGCGAGTAGCCCATTAACTTATGCGGACCGCGTTACCGCACCGG<br>TGCTGGTGATTGCAGGCGAAAACGATCCGCGCTGTCCGATCCGCCAAATCGACAACTA<br>TGTTAACCGCCTGGCCTCGCTGGGCAAGCGTCATGAAGTGTATCGTTATGACGCGGGG<br>CACGGCTCTCTTGTGGCTGACGAACGCATCAAACAGATCGAAGCAACCATTGCGTTTG<br>CTCGCACCCACCTACCCGCCTAG

Table S2. ^{1}H, ^{13}C and ^{15}N NMR assignments for **34** in DMSO-d_6 (0.2% TFA).

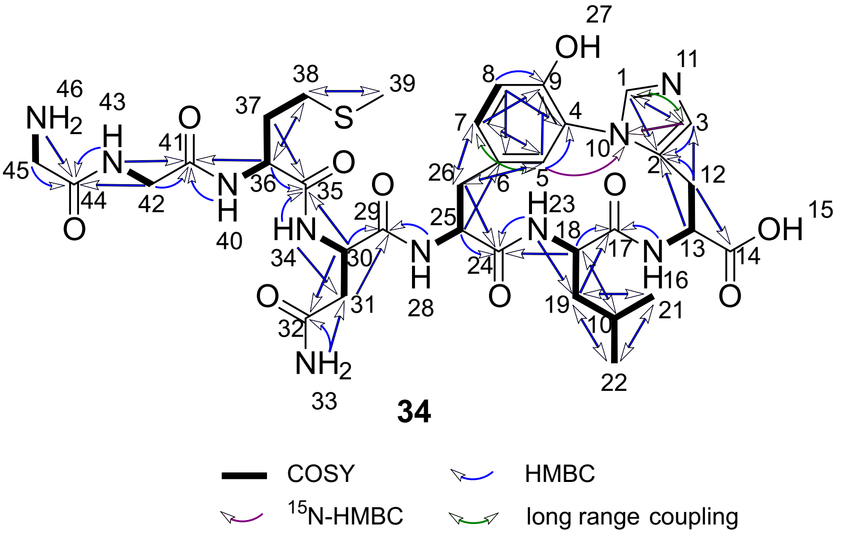

	Position	δ_C[a]	δ_N[b]	type	δ_H[c], (J in Hz)	COSY[d]	HMBC[e]
His	1	135.4	-	CH	9.38, s	3	2, 3
	2	130.4	-	C	-	-	1, 3, 12
	3	121.0	-	CH	7.08, s	1	1, 10, 12
	10	-	182.7	N			
	11	-	-	N			
	12	26.9	-	CH_2	3.31; 2.84, m[g]	13	2, 3, 13, 14
	13	50.7	-	CH	4.54, m[g]	12, 16	2, 12
	14	168.3	-	C	-	-	-
	15	-	-	OH	-[f]	-	-
	16	-	118.5	NH	8.87, d (9.5)	13	17
Tyr	4	121.7	-	C	-	-	-
	5	128.6	-	CH	6.81, s	7	4, 7, 9, 10, 26
	6	127.7	-	C	-	-	-
	7	132.0	-	CH	7.11, d (7.6)	5, 8	4, 5, 8, 26
	8	116.7	-	CH	7.02, d (7.6)	7	4, 6, 7, 9
	9	148.8	-	C	-	-	5, 7, 8
	24	169.5	-	C	-	-	-
	25	53.0	-	CH	4.58, m[g]	26, 28	6, 24, 26, 29
	26	35.9	-	CH_2	2.91; 2,74, m[g]	25	5, 6, 7, 24, 25
	27	-	-	OH	-[f]	-	-
	28	-	113.3	NH	7.21, d (7.1)	25	29
Leu	17	172.7	-	C	-	-	-
	18	50.7	-	CH	4.59, m[g]	19, 23	17, 19, 20, 24
	19	41.9	-	CH_2	1.51; 1.41, m	18, 20	17, 18, 20, 21, 22
	20	24.1	-	CH	1.63, m	19, 21, 22	18, 19, 21, 22
	21	23.0	-	CH_3	0.90, d (6.5)	20	19, 20, 22
	22	21.8	-	CH_3	0.85, d (6.3)	20	19, 20, 21
	23	-	122.3	NH	8.72, d (8.1)	18	18, 19, 24
Asn	29	170.0	-	C	-	-	-
	30	49.8	-	CH	4.52, m[g]	31, 34	29, 31, 32, 35
	31	36.7	-	CH_2	2.51; 2.37, m[g]	30	29, 30, 32
	32	171.4	-	C	-	-	-
	33	-	109.6	NH_2	7,40; 6.93, s	33	31, 32
	34	-	119.1	NH	8.36, d (7.5)	30	30, 31, 35
Met	35	171.2	-	C	-	-	-
	36	51.5	-	CH	4.42, dt (8.3, 4.4)	37, 40	35, 37, 38, 41
	37	32.1	-	CH_2	1.89; 1.73, m[g]	36, 38	35, 36, 38
	38	29.4	-	CH_2	2.41, m[g]	37	36, 37, 39
	39	14.6	-	CH_3	2.01, s	-	38
	40	-	117.2	NH	8.27, d (8.2)	36	36, 41
Gly-1	41	168.3	-	C	-	-	-
	42	41.9	-	CH_2	3.86, d (4,8)	43	41, 44
	43	-	107.8	NH	8.62, t (4.8)	42	41, 42, 44
Gly-2	44	166.3	-	C	-	-	-

45	40.0	-	CH_2	3.60, m	46	44
46	-	28.0	NH_2	8.07, m	45	44, 45

[a]Acquired at 125 MHz, adjusted to the solvent signal of DMSO-d_6 (δ_C 39.51 ppm). [b]Acquired at 71 MHz. [c]Acquired at 500 MHz, adjusted to the solvent signal of DMSO-d_6 (δ_H 2.50 ppm). [d]Proton showing COSY correlation to indicated protons. [e]Protons showing HMBC correlations to indicated carbons. [f]Signals not observed. [g]Overlapping signals.

Table S3. ^{1}H, ^{13}C and ^{15}N shift comparison for the aromatic region of **34** and Myxarylin in DMSO-d_6.[25]

	Position	δ_C[a], type (**34**)	δ_N[b], type (**34**)	δ_H[c], (J in Hz) (**34**)	δ_C[d], type (Myxarylin)	δ_N[b], type (Myxarylin)	δ_H[e], (J in Hz) (Myxarylin)
His	1	135.4, CH	-	9.38, s	135.6, CH	-	9.35, s
	2	130.3, C	-	-	130.6, C	-	-
	3	121.0, CH		7.08, s	120.4, CH	-	7.16, s
	10	-	182.6, N	-	-	174.6, N	-
	11	-	*f*	-	-	247.3, N	-
Tyr	4	121.7, C	-	-	125.3, C	-	-
	5	128.6, CH	-	6.81, s	127.8, CH	-	6.81, d (2.25)
	6	127.7, C	-	-	-	-	-
	7	132.0, CH	-	7.12, d (8.1)	131.8, CH	-	7.23, dd (8.24, 2.25)
	8	116.7, CH	-	7.02, d (8.1)	116.4, CH	-	7.08 d (8.34)
	9	148.8, C	-	-	149.3, C	-	-

[a]Acquired at 125 MHz, adjusted to the solvent signal of DMSO-d_6 (δ_C 39.51 ppm). [b]Acquired at 71 MHz. [c]Acquired at 500 MHz, adjusted to the solvent signal of DMSO-d_6 (δ_H 2.50 ppm). [d]Acquired at 175 MHz, adjusted to the solvent signal of DMSO-d_6 (δ_C 39.51 ppm). [d]Acquired at 500 MHz, adjusted to the solvent signal of DMSO-d_6 (δ_H 2.50 ppm). [e]Acquired at 700 MHz, adjusted to the solvent signal of DMSO-d_6 (δ_H 2.50 ppm). [f]Signals not observed.

Table S4. X-ray data collection and refinement statistics.

	**CorZ_SAH**	**CorZ_SAH_Myxarylin**
**PDB ID**	9SOT	9SOV
**Data collection**		
Space group	I 1 2 1	I 1 2 1
Cell dimension		
a, b, c (Å)	83.43, 117.87, 107.15	83.44, 117.84, 106.91
α, β, γ (°)	90.0, 99.6, 90.0	90.0, 100.2, 90.0
Wavelength (Å)	1.03	1.03
Resolution	45.33 – 2.50 (2.60 – 2.50) *	45.38 – 2.70 (2.83 – 2.70) *
R_{sym} or R_{merge}	0.079 (0.65)	0.122 (1.08)
R_{pim}	0.029 (0.26)	0.034 (0.30)
CC (1/2)	0.99 (0.93)	0.99 (0.84)
I / σI	20.8 (4.3)	15.2 (2.5)
Completeness (%)	98.9 (96.5)	95.7 (97.3)
Redundancy	14.1 (13.9)	14.5 (14.2)
Refinement		
Resolution (Å)	45.33 – 2.50	45.38 – 2.70
No. reflection	35002 (2572)	26730 (2694)
R_{work} / R_{free}	0.206 / 0.249	0.227 / 0.271
No. of atoms	6219	6311
Macromolecule	6185	6137
Ligands	78	174
Solvent	34	61
Protein residues	789	787
B-factors	76.90	68.60
Macromolecules	76.97	68.62
Water	62.80	65.92
R. m. s deviations		
Bond length (Å)	0.004	0.003
Bond angels (°)	0.75	0.65
MolProbity score	1.27	1.57

*Values in parentheses are for the highest-resolution shell

Table S5. Reaction conditions used in this study.

Modification (Enzyme)	Condition
	Reaction buffer: 50 mM Tris-HCl (pH 7.5), 150 mM NaCl
Biaryl-linkage ($P450_{BytO}$ or mutants)	Reaction buffer + (redox partners, 1000 U ml^{-1} catalase and 0.5 mM NADPH (or NADH with Pdx/Pdr).<br>Redox Partner:<br>YkuN/FdR: 25 µM and 2.5 µM<br>Pdx/Pdr: 25 µM and 2.5 µM<br>Spinach Fdx/FdR: 3.5 µM and. 0.1 U ml^{-1}<br>$P450_{BytO}$: 2.5 µM
Leader peptide cleavage (Pro_{TM32})	Reaction buffer or Dulbecco's Phosphate buffered saline (Sigma-Aldric)
Leader peptide cleavage (Pronase E)	Reaction buffer
Leader peptide cleavage (Non-enzymatic)	50 mM Tris 9.0
Methylation (CorZ)	Reaction buffer + (2 mM SAM)
Thioamidation (ThoH/I)	Reaction buffer + (20 mM $MgCl_2$, 5 mM ATP and 2 mM Na_2S)
Dehydration (ThoC/D)	Reaction buffer + (20 mM $MgCl_2$ and 5 mM ATP)

Table S6. Primers used in this study. All sequences are provided 5' to 3'. The suffices "F" and "R" indicate forward or reverse primer, respectively. pET-16b based YkuN and FdR templates used for cloning were provided by Prof. Vlada B. Urlacher.

Primer name	Sequence (5'-3')	Application
$P450_{BytO}$-NdeI-F	CTGGTGCCGCGCGGCAGCCATATGGTGCCCACCGTCGTCGAG	Cloning of $P450_{BytO}$ into pET-28b
$P450_{BytO}$-HindIII-R	GTGCTCGAGTGCGGCCGCAAGCTTTCACGCGCCGAATGTGAC	
$P450_{BytO}$-F-His226Val	CGCGTCGTGTTCGTGGCCGGCTACACCTCC	Cloning of $P450_{BytO}$ mutant (His226Val)
$P450_{BytO}$-R-His226Val	GTGTAGCCGGCCACGAACACGACGCGCATG	
$P450_{BytO}$-F-Ser78Asn	TGAGCATCCAAAACATGGACCCGCCCGA	Cloning of $P450_{BytO}$ mutant (Ser78Asn)
$P450_{BytO}$-R-Ser78Asn	GGCGGGTCCATGTTTTGGATGCTCACGC	
$P450_{BytO}$-F-Ile160Val	TGTCGGACGCCGTGATCCGCGGCATGGA	Cloning of $P450_{BytO}$ mutant (Ile160Val)
$P450_{BytO}$-R-Ile160Val	ATGCCGCGGATCACGGCGTCCGACATC	
$P450_{BytO}$-F-Pro272Ala	TGTTCCGGTATGACGGCGCGGTGCAGGCCACCGGCCGCGT	Cloning of $P450_{BytO}$ mutant (Pro272Ala)
$P450_{BytO}$-R-Pro272Ala	CGCGCCGTCATACCGGAACAGCT	
$P450_{BytO}$-R-Pro272Ser	TGTTCCGGTATGACGGCTCGGTGCAGGCCACCGGCCGCGT	Cloning of $P450_{BytO}$ mutant (Pro272Ser)
$P450_{BytO}$-R-Pro272Ser	CGAGCCGTCATACCGGAACAGCT	
CorZ-NdeI-F	GCCTGGTGCCGCGCGGCAGCCATATGGACTTCGACTACACCGACGATGCGGC	Cloning of CorZ into pET-28b
CorZ-HindIII-R	GGTGCTCGAGTGCGGCCGCAAGCTTTCACGGCGCGGCGCGGGCCC	
CorZ-F-Phe109Ala	ATCACCGTGCCCGCCCGCACCTTCCTC	Cloning of CorZ mutant (Phe109Ala) into pET-28b
CorZ-R-Phe109Ala	GAAGGTGCGGGCGGGCACGGTGATGAG	
CorZ-F-Phe109Gly-Arg110Gly	ATCACCGTGCCCGGCGGCACCTTCCTCCACCT	Cloning of CorZ mutant (Phe109Gly & Arg110Gly) into pET-28b
CorZ-R-Phe109Gly-Arg110Gly	GTGGAGGAAGGTGCCGCCGGGCACGGTGATGAG	
Precursor peptide-F-**33**/**36**	CTTTAATAAGGAGATATACCATGGGTAGCAGCCATCACCATCATCAT	Cloning of **33** – **36** precursor peptides into MCS-1 of pRSFDuet-1
Precursor peptide-R-**33**	CTTAAGCATTATGCGGCCGCAAGCTTTTAATGCAGATAGTTCATGCCCTGAAAATACAGGTTTTCGG	
Precursor peptide-R-**34**	CTTAAGCATTATGCGGCCGCAAGCTTTTAATGCAGATAGTTCATGCCGCCCTGAAAATACAGGTTTTCGG	
Precursor peptide-R-**35**	CTTAAGCATTATGCGGCCGCAAGCTTTTAATGCAGATAGTTCATGCCGCCGCCCTGAAAATACAGGTTTTCGG	
Precursor peptide-R-**36**	CTTAAGCATTATGCGGCCGCAAGCTTTTAATGCAGATAGTTCATGCCGCCGCCGCCCTGAAAATACAGGTTTTCGG	
$P450_{BytO}$-F-coexpress	GTATAAGAAGGAGATATACATATGGTGCCCACCGTCGTCGAGT	Cloning of $P450_{BytO}$ into MCS-2 of pRSFDuet-1
$P450_{BytO}$-R-coexpress	AGCGGTTTCTTTACCAGACTCGAGTCACGCGCCGAATGTGACAG	

FdR-F-coexpress	CTTTAAGAAGGAGATATACCATGGCTGATTGGGTAACAGGCAAAGTC	Cloning of FdR into MCS-1 of pETDuet-1
FdR-R-coexpress	CTTAAGCATTATGCGGCCGCAAGCTTTTACCAGTAATGCTCCGCTGT	
YkuN-F-coexpress	GTATAAGAAGGAGATATACATATGGCTAAAGCCTTGATTACATATGCCA	Cloning of YkuN into MCS-2 of pETDuet-1
YkuN-R-coexpress	AGCGGTTTCTTTACCAGACTCGAGTTATGAAACATGGATTTTTTCCTTGTTCAT	
Hybrid precursor peptide-F-**40**	AACTTTAATAAGGAGATATACCATGGGTAGCAGCCATCACCATCATCATCACGG	Cloning of hybrid precursor peptide (**40**) into pHis-SUMO-TEV
Hybrid precursor peptide-R-**40**	TAAGCATTATGCGGCCGCAAGCTTTTAATGCAGATAGTTCATGCCGCCGGCGGCCGCCATCACGCT	

References

1. Sikandar, A.; Lopatniuk, M.; Luzhetskyy, A.; Muller, R.; Koehnke, J., Total In Vitro Biosynthesis of the Thioamitide Thioholgamide and Investigation of the Pathway. *J Am Chem Soc* **2022,** *144* (11), 5136-5144.
2. Omura, T.; Sato, R., The Carbon Monoxide-Binding Pigment of Liver Microsomes. Ii. Solubilization, Purification, and Properties. *J Biol Chem* **1964,** *239*, 2379-85.
3. von Buhler, C.; Le-Huu, P.; Urlacher, V. B., Cluster screening: an effective approach for probing the substrate space of uncharacterized cytochrome P450s. *Chembiochem* **2013,** *14* (16), 2189-98.
4. Kuznetsov, V. Y.; Blair, E.; Farmer, P. J.; Poulos, T. L.; Pifferitti, A.; Sevrioukova, I. F., The putidaredoxin reductase-putidaredoxin electron transfer complex: theoretical and experimental studies. *J Biol Chem* **2005,** *280* (16), 16135-42.
5. Lawson, R. J.; von Wachenfeldt, C.; Haq, I.; Perkins, J.; Munro, A. W., Expression and characterization of the two flavodoxin proteins of Bacillus subtilis, YkuN and YkuP: biophysical properties and interactions with cytochrome P450 BioI. *Biochemistry* **2004,** *43* (39), 12390-409.
6. McIver, L.; Leadbeater, C.; Campopiano, D. J.; Baxter, R. L.; Daff, S. N.; Chapman, S. K.; Munro, A. W., Characterisation of flavodoxin NADP+ oxidoreductase and flavodoxin; key components of electron transfer in Escherichia coli. *Eur J Biochem* **1998,** *257* (3), 577-85.
7. Sevrioukova, I. F.; Poulos, T. L., Putidaredoxin reductase, a new function for an old protein. *J Biol Chem* **2002,** *277* (28), 25831-9.
8. Abramson, J.; Adler, J.; Dunger, J.; Evans, R.; Green, T.; Pritzel, A.; Ronneberger, O.; Willmore, L.; Ballard, A. J.; Bambrick, J.; Bodenstein, S. W.; Evans, D. A.; Hung, C. C.; O'Neill, M.; Reiman, D.; Tunyasuvunakool, K.; Wu, Z.; Zemgulyte, A.; Arvaniti, E.; Beattie, C.; Bertolli, O.; Bridgland, A.; Cherepanov, A.; Congreve, M.; Cowen-Rivers, A. I.; Cowie, A.; Figurnov, M.; Fuchs, F. B.; Gladman, H.; Jain, R.; Khan, Y. A.; Low, C. M. R.; Perlin, K.; Potapenko, A.; Savy, P.; Singh, S.; Stecula, A.; Thillaisundaram, A.; Tong, C.; Yakneen, S.; Zhong, E. D.; Zielinski, M.; Zidek, A.; Bapst, V.; Kohli, P.; Jaderberg, M.; Hassabis, D.; Jumper, J. M., Accurate structure prediction of biomolecular interactions with AlphaFold 3. *Nature* **2024,** *630* (8016), 493-500.
9. Picache, J. A.; Rose, B. S.; Balinski, A.; Leaptrot, K. L.; Sherrod, S. D.; May, J. C.; McLean, J. A., Collision cross section compendium to annotate and predict multi-omic compound identities. *Chem Sci* **2019,** *10* (4), 983-993.
10. Cianci, M.; Bourenkov, G.; Pompidor, G.; Karpics, I.; Kallio, J.; Bento, I.; Roessle, M.; Cipriani, F.; Fiedler, S.; Schneider, T. R., P13, the EMBL macromolecular crystallography beamline at the low-emittance PETRA III ring for high- and low-energy phasing with variable beam focusing. *J Synchrotron Radiat* **2017,** *24* (Pt 1), 323-332.
11. Winter, G.; Lobley, C. M.; Prince, S. M., Decision making in xia2. *Acta Crystallogr D Biol Crystallogr* **2013,** *69* (Pt 7), 1260-73.
12. Kabsch, W., Xds. *Acta Crystallogr D Biol Crystallogr* **2010,** *66* (Pt 2), 125-32.
13. Liebschner, D.; Afonine, P. V.; Baker, M. L.; Bunkoczi, G.; Chen, V. B.; Croll, T. I.; Hintze, B.; Hung, L. W.; Jain, S.; McCoy, A. J.; Moriarty, N. W.; Oeffner, R. D.; Poon, B. K.; Prisant, M. G.; Read, R. J.; Richardson, J. S.; Richardson, D. C.; Sammito, M. D.; Sobolev, O. V.; Stockwell, D. H.; Terwilliger, T. C.; Urzhumtsev, A. G.; Videau, L. L.; Williams, C. J.; Adams, P. D., Macromolecular structure determination using X-rays, neutrons and electrons: recent developments in Phenix. *Acta Crystallogr D Struct Biol* **2019,** *75* (Pt 10), 861-877.
14. Emsley, P.; Lohkamp, B.; Scott, W. G.; Cowtan, K., Features and development of Coot. *Acta Crystallogr D Biol Crystallogr* **2010,** *66* (Pt 4), 486-501.
15. Williams, C. J.; Headd, J. J.; Moriarty, N. W.; Prisant, M. G.; Videau, L. L.; Deis, L. N.; Verma, V.; Keedy, D. A.; Hintze, B. J.; Chen, V. B.; Jain, S.; Lewis, S. M.; Arendall, W. B., 3rd; Snoeyink, J.; Adams, P. D.; Lovell, S. C.; Richardson, J. S.; Richardson, D. C., MolProbity: More and better reference data for improved all-atom structure validation. *Protein Sci* **2018,** *27* (1), 293-315.
16. Wallace, A. C.; Laskowski, R. A.; Thornton, J. M., LIGPLOT: a program to generate schematic diagrams of protein-ligand interactions. *Protein Eng* **1995,** *8* (2), 127-34.
17. Frydendall, E. K.; Scott, E. E., Development of a high throughput cytochrome P450 ligand-binding assay. *J Biol Chem* **2024,** *300* (10), 107799.
18. Padva, L.; Gullick, J.; Coe, L. J.; Hansen, M. H.; De Voss, J. J.; Crusemann, M.; Cryle, M. J., The Biarylitides: Understanding the Structure and Biosynthesis of a Fascinating Class of Cytochrome P450 Modified RiPP Natural Products. *Chembiochem* **2025,** *26* (7), e202400916.
19. Zdouc, M. M.; Alanjary, M. M.; Zarazua, G. S.; Maffioli, S. I.; Crusemann, M.; Medema, M. H.; Donadio, S.; Sosio, M., A biaryl-linked tripeptide from Planomonospora reveals a widespread class of minimal RiPP gene clusters. *Cell Chem Biol* **2021,** *28* (5), 733-739 e4.
20. Zallot, R.; Oberg, N.; Gerlt, J. A., The EFI Web Resource for Genomic Enzymology Tools: Leveraging Protein, Genome, and Metagenome Databases to Discover Novel Enzymes and Metabolic Pathways. *Biochemistry* **2019,** *58* (41), 4169-4182.
21. Elfmann, C.; Stulke, J., PAE viewer: a webserver for the interactive visualization of the predicted aligned error for multimer structure predictions and crosslinks. *Nucleic Acids Res* **2023,** *51* (W1), W404-W410.

22. Mathias H. Hansen, A. K., Maxine Treisman, Vishnu Mini Sasi, Laura Coe, Yongwei Zhao, Leo Padva, Caroline Hess, Victor Leichthammer, Daniel L. Machell, Ralf B. Schittenhelm, Colin J. Jackson, Julien Tailhades, Max Crüsemann, James J. De Voss, Elizabeth H. KrenskeMax J. Cryle, Structural Insights into a Side Chain Cross-Linking Biarylitide P450 from RiPP Biosynthesis. *ACS Cent Sci* **2024,** *14* (2), 812-826.
23. Sievers, F.; Higgins, D. G., Clustal Omega for making accurate alignments of many protein sequences. *Protein Sci* **2018,** *27* (1), 135-145.
24. Waterhouse, A. M.; Procter, J. B.; Martin, D. M.; Clamp, M.; Barton, G. J., Jalview Version 2--a multiple sequence alignment editor and analysis workbench. *Bioinformatics* **2009,** *25* (9), 1189-91.
25. Hug, J. J.; Frank, N. A.; Walt, C.; Senica, P.; Panter, F.; Muller, R., Genome-Guided Discovery of the First Myxobacterial Biarylitide Myxarylin Reveals Distinct C-N Biaryl Crosslinking in RiPP Biosynthesis. *Molecules* **2021,** *26* (24).
